# Diverse phloroglucinols with *h*AChE inhibitory and anti-VRE effects from *Rhodomyrtus tomentosa* fruits

**DOI:** 10.1007/s13659-025-00557-0

**Published:** 2026-01-08

**Authors:** Ling-Yun Chen, Mu-Yuan Yu, E-E Luo, Wen-Ying Zong, Shu-Mei Lei, Yu Pan, Ai-Chun Lu, Cheng-Qin Liang, Xu-Jie Qin

**Affiliations:** 1 College of Pharmacy, Guilin Medical University, Guilin, 541199 People’s Republic of China; 2 State Key Laboratory of Phytochemistry and Natural Medicines, Kunming Institute of Botany, Chinese Academy of Sciences, Kunming, 650201 People’s Republic of China; 3 University of Chinese Academy of Sciences, Beijing, 100049 People’s Republic of China

**Keywords:** *Rhodomyrtus tomentosa* fruits, Phloroglucinols, *h*AChE inhibitory activity, Anti-VRE activity, Molecular docking

## Abstract

*Rhodomyrtus tomentosa* fruits serve as both functional food and medicinal resources due to their rich bioactive constituents and manifold pharmacological effects. Phytochemical exploration of the *R. tomentosa* fruits led to the identification of eight new polymethylated phloroglucinols, designated as rhodotomentodione F (**1**) and rhodotomentodimers H‒N (**2**‒**8**), along with six previously described congeners (**9**‒**14**). Based on the detailed inspection of comprehensive spectroscopic data, electronic circular dichroism (ECD) simulations, and nuclear magnetic resonance (NMR) calculations, and DP4+ analyses, the structures of phloroglucinols **1**‒**8** were determined. Heterodimeric phloroglucinols **3**‒**14** exhibited human acetylcholinesterase (*h*AChE) inhibitory activities, with **13** exhibiting the highest potency (IC_50_ = 1.04 μM). Moreover, molecular docking analysis clarified the potential binding interactions between the most active phloroglucinol **13** with *h*AChE. In addition, phloroglucinols **11** and **12** displayed significant anti-VRE (vancomycin-resistant *Enterococci*) activities, with MIC values reaching as low as 1 μg/mL.

**Graphical Abstract:**

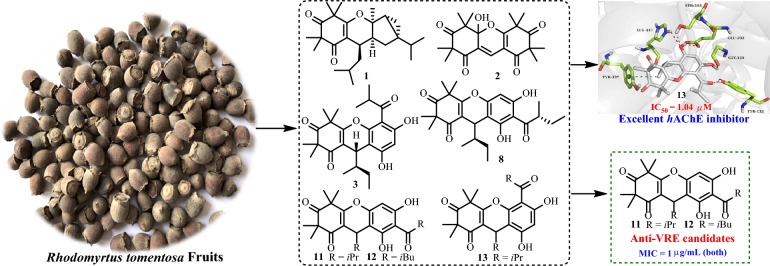

**Supplementary Information:**

The online version contains supplementary material available at 10.1007/s13659-025-00557-0.

## Introduction

**Fig. 1:**
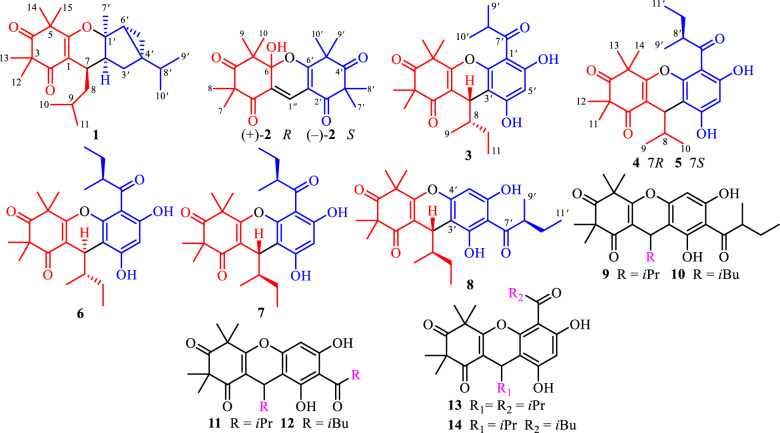
Chemical structures of phloroglucinols **1**–**14**

Phloroglucinols derived from species within the Myrtaceae family have garnered significant attention from scientific community due to their complex molecular architectures and notable biological activities [1]. *Rhodomyrtus tomentosa*, a medicinal and edible plant of this family, is extensively cultivated across Thailand, Vietnam, and southeastern China [2]. The *R. tomentosa* fruits are popularly used as ingredients of pies and jams in these regions [3]. Furthermore, local populations traditionally infuse the fruits into white spirits to prepare herbal liquors, which are valued for their enhanced flavor and health-promoting and disease-preventive properties. Historically, the fruits, have also been utilized for the management of diarrhea and dysentery [3]. Previous studies have demonstrated that crude extracts and phenolic constituents from *R. tomentosa* fruits exhibit antioxidant [4–6], anti-inflammatory [7, 8], hepatoprotective effects against nonalcoholic fatty liver disease [9], and general health-promoting benefits [10]. Despite these findings, only one phloroglucinol homodimer with anti-inflammatory activity, namely watsonianone A [11], has been identified from the fruits of title plant, leaving the broader phloroglucinol composition largely unexplored. In contrast, phloroglucinol compounds isolated from *R. tomentosa* leaves in tropical regions of China have been characterized for both structures and their bioactivities [12–16]. Therefore, further elucidation of novel bioactive phloroglucinol components from its fruits represents a valuable research direction. To obtain additional active phloroglucinol constituents, the fruits were subjected to ultraviolet-guided employing a DAD detector. Consequently, eight newly reported polymethylated phloroglucinols (**1**–**8**) and six known analogues (**9**–**14**) were obtained (Fig. 1). Selected compounds were examined for their *h*AChE inhibitory and anti-VRE activities. For the most potent *h*AChE inhibitor, molecular docking analysis was conducted to elucidate the possible interaction mechanism. This study presents the isolation, comprehensive structural characterization, and preliminary pharmacological assessment of the isolated metabolites.

## Results and discussion

The phloroglucinol containing moiety of *R. tomentosa* fruits was subjected to sequential chromatographic separation on silica gel, reversed-phase C18, Sephadex LH-20, and semi-preparative HPLC, leading to the isolation of one new polymethylated phloroglucinol meroterpenoid (PPM, **1**), one new polymethylated phloroglucinol homodimer (PPHMD, **2**), and six polymethylated phloroglucinol heterodimers (PPHTDs, **3**–**8**). In addition, six previously reported congeners were structurally identified as myrciarone B (**9**) [17, 18], rhodotomentodimer D (**10**) [13], isomyrtucommulone B (**11**) [17, 18], rhodomyrtone (**12**) [19], myrtucommulone B (**13**) [20], and callistenone A (**14**) [21], based on detailed comparison of their NMR and mass spectrometry (MS) profiles with reference data previously documented in the literature.

**Fig. 2:**
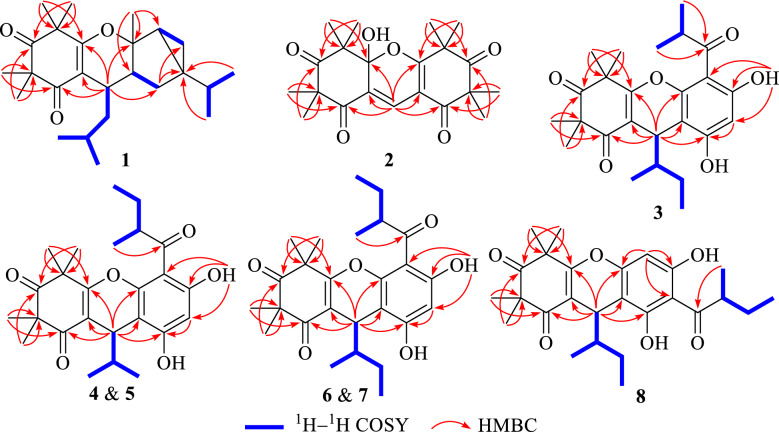
Key ^1^H–^1^H COSY, and HMBC correlations of phloroglucinols **1**‒**8**

Phloroglucinol **1** was assigned the molecular formula of C_25_H_38_O_3_, as determined by an high-resolution electrospray ionization mass spectrometry (HRESIMS) with an [M + H]^+^ ion at *m*/*z* 387.2904 (calcd for C_25_H_39_O_3_, 387.2894), indicating seven degrees of unsaturation. The ^1^H NMR spectrum (Table 1) displayed signals for nine methyl groups at *δ*_H_ 0.85 (d, *J* = 6.8 Hz, H_3_-10*'*), 0.86 (d, *J* = 6.8 Hz, H_3_-9*'*), 0.89 (d, *J* = 6.6 Hz, H_3_-11), 0.95 (d, *J* = 6.6 Hz, H_3_-10), 1.33 (s, H_3_-13), 1.34 (s, H_3_-12), 1.38 (s, H_3_-15), 1.40 (s, H_3_-14), and 1.42 (s, H_3_-7′), along with two strongly shielded methylene protons at *δ*_H_ 0.51 (2H, brd, *J* = 5.4 Hz, H_2_-5*'*), characteristic of a cyclopropane moiety. Analysis of the ^13^C NMR data of phloroglucinol **1** (Table 1), supported by DEPT and HSQC experiments, disclosed the presence of 25 distinct carbon signals, including two keto carbonyl groups at *δ*_C_ 198.3 (C-2) and 213.6 (C-4), two olefinic quaternary carbons at *δ*_C_ 109.2 (C-1) and 168.8 (C-6), and additional four quaternary carbons, one of which was oxygenated at *δ*_C_ 87.3 (C-1*'*). The remaining signals were attributed to five methines, three methylenes, and nine methyl groups. Three of the seven degrees of unsaturation were attributed to the presence of two ketone functionalities and a single olefinic bond, while the remaining unsaturation implied the existence of a tetracyclic core structure. The planar architecture of phloroglucinol **1** was determined through comprehensive analyses of ^1^H–^1^H COSY and HMBC spectra (Fig. 2). The ^1^H–^1^H COSY experiment established an isopentyl unit of *δ*_H_ 2.64 (H-7)/1.29 (H_2_-8a) and 1.23 (H_2_-8b)/1.67 (H-9)/0.95 (H_3_-10) and 0.89 (H_3_-11). Key HMBC interactions, from *δ*_H_ 2.64 (H-7) to *δ*_C_ 109.2 (C-1), 168.8 (C-6), and 198.3 (C-2); from *δ*_H_ 1.34 (H_3_-12) and 1.33 (H_3_-13) to *δ*_C_ 55.5 (C-3), 98.3 (C-2) and 213.6 (C-4); and from *δ*_H_ 1.40 (H_3_-14) and 1.38 (H_3_-15) to *δ*_C_ 47.7 (C-5), 168.8 (C-6), and 213.6 (C-4), strongly supported the presence of a *β*-triketone core. Likewise, apart from three structural fragments of *δ*_H_ 1.85 (H-2*'*)*/*1.34 and 1.74 (H_2_-3*'*), 0.51 (2H, H_2_-5*'*)/1.36 (H-6*'*), and 1.29 (H-8*'*)/0.85 (H_3_-9*'*) and 0.86 (H_3_-10*'*) as indicated by the ^1^H–^1^H COSY spectrum, HMBC correlations (Fig. 2), specifically from *δ*_H_ 1.74 (H-3*'*a) to *δ*_C_ 14.2 (C-5*'*) and 32.4 (C-4*'*), from *δ*_H_ 1.42 (H_3_-7*'*) to *δ*_C_ 34.9 (C-6*'*), 37.6 (C-2*'*), and 87.3 (C-1*'*), and from *δ*_H_ 0.86 (H_3_-9*'*) and 0.85 (H_3_-10*'*) to *δ*_C_ 32.4 (C-4*'*) confirmed the presence of a thujene unit. Additional HMBC cross-peaks from *δ*_H_ 2.64 (H-7) to *δ*_C_ 34.0 (C-3*'*) and 87.3 (C-1*'*) indicated that the *β*-triketone moiety and the thujene fragment was connected through a C-6‒*O*‒C-1*'* ether linkage and a C-7‒C-2*'* bond. ROSEY correlations between *δ*_H_ 1.85 (H-2*'*) and 0.51 (H-5*'*), 1.42 (H_3_-7*'*), and 2.64 (H-7) supported an *α* orientation of all these protons. The assignment of absolute configuration for phloroglucinol **1** was achieved by comparing its measured ECD spectrum with that simulated by TDDFT B3LYP/6–31++ G(2d,p) using Gaussian 16. The experimental curve (Fig. 4) exhibited negative Cotton effects at 205 and 271 nm and a positive signal at 311 nm, closely matching the calculated spectrum, thereby confirming the (7*R*,1*'R*,2*'S*,4*'R*,6*'R*) configuration. Based on combined spectroscopic and computational evidence, phloroglucinol **1**, was structurally identified as a novel *β*-triketone‒thujene meroterpenoid and designated rhodotomentodione F.

**Table 1:** ^13^C (150 MHz) and ^1^H (600 MHz) NMR data of phloroglucinol **1** in CDCl_3_

*β*-triketone moiety			*α*-thujene unit		
No	*δ*_C_	*δ*_H_, mult. (*J* in Hz)	No	*δ*_C_	*δ*_H_, mult. (*J* in Hz)
1	109.2, s		1*'*	87.3, s	
2	198.3, s		2*'*	37.6, d	1.85, dd (11.1, 7.5)
3	55.5, s		3*'*	34.0, t	a 1.74, dd (12.3, 7.5)
4	213.6, s				b 1.34, overlapped
5	47.7, s		4*'*	32.4, s	
6	168.8, s		5*'*	14.2, t	0.51, 2H brd (5.4)
7	28.7, d	2.64, dd (10.7, 3.9)	6*'*	34.9, d	1.36, brd (7.0)
8	42.6, t	a 1.29, m	7*'*	23.7, q	1.42, s
		b 1.23, m	8*'*	32.3, d	1.29, overlapped
9	25.7, d	1.67, m	9*'*	19.6, q	0.86, d (6.8)
10	21.1, q	0.95, d (6.6)	10*'*	20.0, q	0.85, d (6.8)
11	23.7, q	0.89, d (6.6)			
12	23.2, q	1.34, s			
13	25.7, q	1.33, s			
14	25.1, q	1.40, s			
15	25.2, q	1.38, s

Phloroglucinol **2** was determined to possess the molecular formula C_21_H_26_O_6_, as indicated by HRESIMS, which displayed a [M + H]^+^ ion at *m*/*z* 375.1802 (cacld for C_21_H_27_O_6_, 375.1802). The ^1^H NMR spectrum of phloroglucinol **2** (Table 2) featured a downfield singlet at *δ*_H_ 8.00 (1H, s, H-1*''*) and eight singlet methyl groups at *δ*_H_ 1.08 (H_3_-9), 1.38 (H_3_-8), 1.40 × 2 (6H, H_3_-7*'*/H_3_-8*'*), 1.41 (H_3_-7), 1.50 (H_3_-10), and 1.53 × 2 (6H, H_3_-9*'*/H_3_-10*'*). The ^13^C NMR data revealed four ketone carbonyl signals at *δ*_C_ 194.9 (C-2*'*), 197.1 (C-2), 210.5 (C-4*'*), and 210.7 (C-4), two double bonds at *δ*_C_ 108.3 (C-1*'*), 122.1 (C-1), 127.7 (C-1*''*), and 172.9 (C-6*'*), a hemiketal carbon at *δ*_C_ 100.8 (C-6), four quaternary carbons at *δ*_C_ 48.0 (C-5*'*), 53.9 (C-5), 54.8 (C-3), and 56.1 (C-3*'*), and eight methyl groups, indicating a tricyclic scaffold. Key HMBC correlations (Fig. 2) from *δ*_H_ 1.41 (H_3_-7) and 1.38 (H_3_-8) to *δ*_C_ 54.8 (C-3), 197.1 (C-2), and 210.7 (C-4), from *δ*_H_ 1.50 (H_3_-10) and 1.08 (H_3_-9) to *δ*_C_ 53.9 (C-5), 100.8 (C-6), and 210.7 (C-4), and from *δ*_H_ 8.00 (H-1*''*) to *δ*_C_ 100.8 (C-6), 122.1 (C-1), and 197.1 (C-2) supported a *β*-triketone motif incorporating a hemiketal at C-6 and a C-1 and C-1*''* double bond. Similarly, HMBC cross-peaks from *δ*_H_ 1.40 (6H, H_3_-7*'* and H_3_-8*'*) to *δ*_C_ 56.1 (C-3*'*), 194.9 (C-2*'*), and 210.5 (C-4*'*), from *δ*_H_ 1.53 (6H, H_3_-9*'*/ H_3_-10*'*) to *δ*_C_ 48.0 (C-5*'*), 172.9 (C-6*'*), and 210.5 (C-4*'*), and from *δ*_H_ 8.00 (H-1*''*) to *δ*_C_ 108.3 (C-1*'*), 172.9 (C-6*'*), and 194.9 (C-2*'*) indicated a second *β*-triketone fragment. Additional HMBC data indicated linkage of the two fragments via a C-6‒*O*‒C-6*'* ether bond and a C-1‒C-1*''*‒C-1*'* connection. Phloroglucinol **2**, identified as a racemic mixture, was resolved into enantiomers (+)-**2** and (−)-**2** using a Daicel CHIRALPAK IC column. ECD spectral analysis (Fig. 4) established the absolute configurations for (+)-**2** and (−)-**2** to be (6*R*) and (6*S*), respectively. Thus, phloroglucinol **2** was structurally characterized as a PPHMD and designated rhodotomentodimer H.

**Table 2:** The ^13^C (150 MHz) and ^1^H (600 MHz) NMR data of phloroglucinol **2** in CDCl_3_

No	*δ*_C_	*δ*_H_, mult. (*J* in Hz)	No	*δ*_C_	*δ*_H_, mult. (*J* in Hz)
1	122.1, s		1*'*	108.3, s	
2	197.1, s		2*'*	194.9, s	
3	54.8, s		3*'*	56.1, s	
4	210.7, s		4*'*	210.5, s	
5	53.9, s		5*'*	48.0, s	
6	100.8, s		6*'*	172.9, s	
7	24.2, q	1.41, s	7*'*	22.9, q	1.40, s
8	26.7, q	1.38, s	8*'*	23.4, q	1.40, s
9	23.4, q	1.08, s	9*'*	24.1, q	1.53, s
10	14.9, q	1.50, s	10*'*	25.4, q	1.53, s
			1*''*	127.7, d	8.00, s

**Fig. 3:**
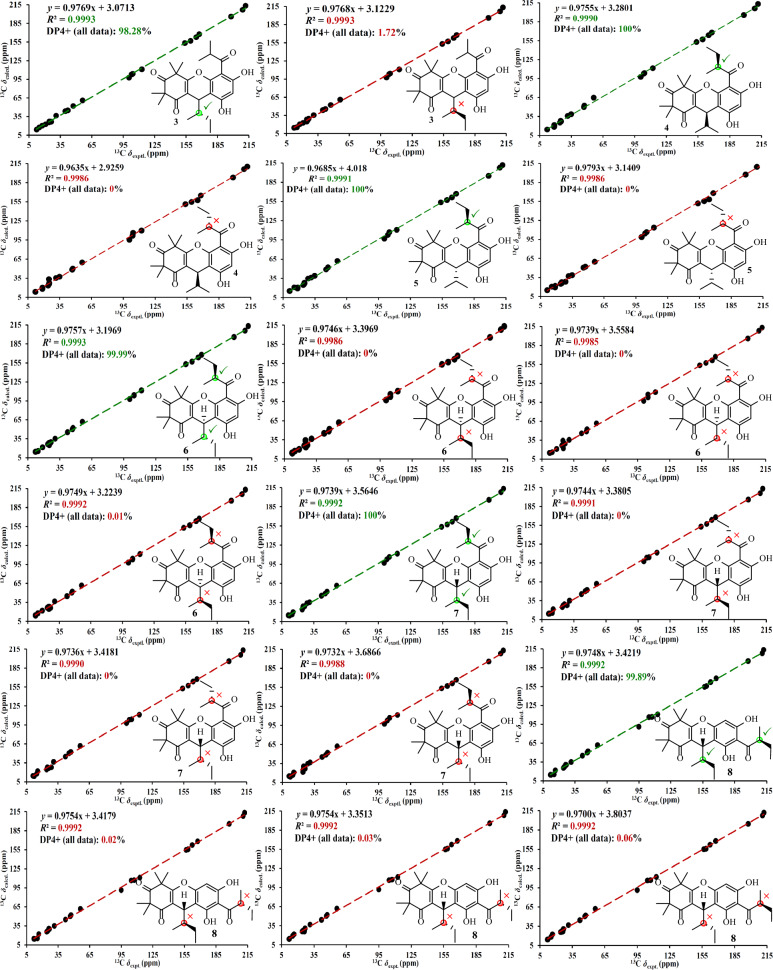
NMR calculations and DP4+ analyses for phloroglucinols **3**–**8**

**Fig. 4:**
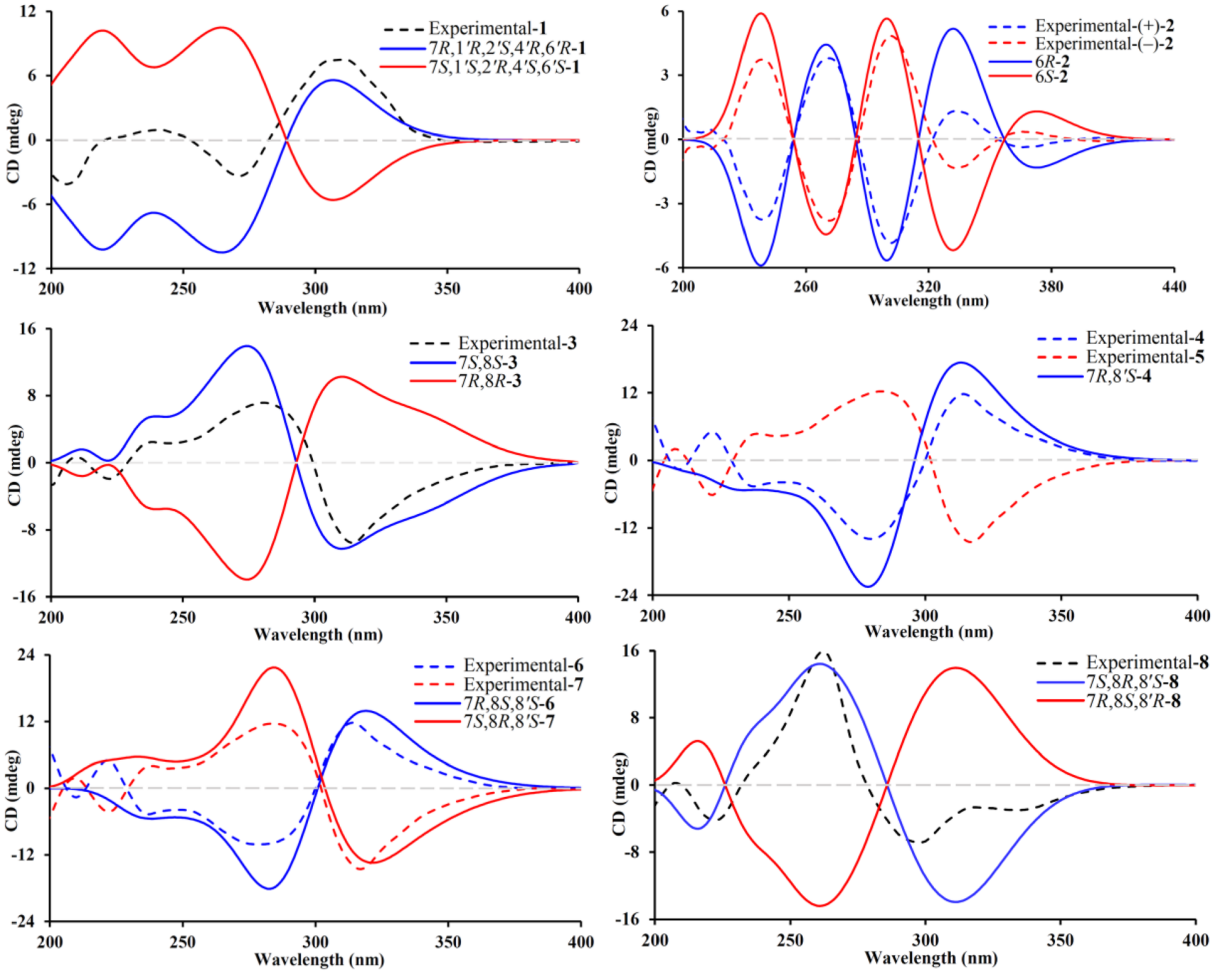
Calculated and experimental ECD spectra of phloroglucinols** 1**‒**8**

Phloroglucinol **3** was determined to possess the molecular formula C_25_H_32_O_6_, as established by its HRESIMS, which exhibited a [M + H]^+^ ion at *m*/*z* 429.2279 (calcd for C_25_H_33_O_6_, 429.2272). The ^1^H NMR spectrum of phloroglucinol **3** (Table 3) indicated four singlet methyl groups at *δ*_H_ 1.39 (H_3_-12), 1.42 (H_3_-14), 1.43 (H_3_-13), and 1.61 (H_3_-15), three doublet methyl groups at *δ*_H_ 0.74 (*J* = 6.8 Hz, H_3_-9), 1.24 (*J* = 7.3 Hz, H_3_-10*'*), and 1.25 (*J* = 6.7 Hz, H_3_-9*'*), a triplet methyl group at *δ*_H_ 0.90 (*J* = 7.3 Hz, H_3_-11). An aromatic proton at *δ*_H_ 6.26 (s, H-5*'*), and a strongly deshielded hydroxyl proton at *δ*_H_ 13.30 (s, OH-6*'*) were also observed. The ^13^C NMR spectrum (Table 3) displayed 25 carbon resonances, including eight methyl groups at *δ*_C_ 12.3 (CH_3_-11), 14.9 (CH_3_-9), 17.7 (CH_3_-9*'*), 20.8 (CH_3_-10*'*), 24.3 (CH_3_-12), 24.5 (CH_3_-13), 24.9 (CH_3_-14) and 25.1 (CH_3_-15), one methylene group at *δ*_C_ 26.1 (CH_2_-10), three methine groups at *δ*_C_ 30.9 (CH-7), 39.7 (CH-8*'*), and 42.1 (CH-8), two aliphatic quaternary carbons at *δ*_C_ 47.2 (C-5) and 56.2 (C-3), a pair of olefinic carbons at *δ*_C_ 112.7 (C-1) and 167.5 (C-6), six aromatic carbons at *δ*_C_ 100.6 (CH-5′), 103.0 (C-3′), 104.0 (C-1′), 153.8 (C-2′), 159.3 (C-4′), and 164.6 (C-6′), and three ketone carbonyl groups at *δ*_C_ 197.9 (C-2), 208.9 (C-7′), and 211.7 (C-4). Comparison of the abovementioned spectral data with those of myrtucommulone B (phloroglucinal **13**) [20] revealed close similarity, with the exception that a 2-methylbutyl unit replaced an isobutyl at C-1 in myrtucommulone B. This structural feature was supported by a 4.35 (H-7)/1.56 (H-8)/1.50 (H_2_-10a) and 1.03 (H_2_-10b) and 0.74 (H_3_-9)/0.90 (H_3_-11) spin system identified through ^1^H–^1^H COSY analysis, along with key HMBC cross-peaks (Fig. 2) from *δ*_H_ 4.35 (H-7) to *δ*_C_ 103.0 (C-2*'*), 112.7 (C-1), 153.8 (C-2*'*), 159.3 (C-4*'*), 167.5 (C-6), and 197.9 (C-2). The H-7 was stochastically assigned as a *β* configuration, and the relative configuration for the side chain at C-7 was determined by a theoretical NMR calculation method (Fig. 3) and DP4+ probability analysis. Finally, the absolute configuration of compound **3** was established as (7*S*,8*S*) based on the ECD calculations (Fig. 4). Thus, phloroglucinol **3** was identified as a PPHTD and designated rhodotomentodimer I.

**Table 3:** ^13^C (150 MHz) and ^1^H (600 MHz) data for phloroglucinols **3**–**5** in CDCl_3_

No	**3**		**4**		**5**	
	*δ*_C_	*δ*_H_, mult. (*J* in Hz)	*δ*_C_	*δ*_H_, mult. (*J* in Hz)	*δ*_C_	*δ*_H_, mult. (*J* in Hz)
1	112.7, s		112.1, s		112.1, s	
2	197.9, s		198.4, s		198.7, s	
3	56.2, s		56.2, s		56.2, s	
4	211.7, s		211.8, s		211.8, s	
5	47.2, s		47.3, s		47.3, s	
6	167.5, s		168.0, s		168.1, s	
7	30.9, d	4.35, d (3.0)	31.5, d	4.35, d (3.5)	31.5, d	4.31, d (3.6)
8	42.1, d	1.56, m	34.8, d	1.91, m	34.8, d	1.92, m
9	14.9, q	0.74, d (6.8)	18.7, q	0.83, d (6.9)	18.6, q	0.79, d (6.9)
10	26.1, t	a 1.50, m	18.8, q	0.79, d (6.9)	18.8, q	0.84, d (6.9)
		b 1.03, m				
11	12.3, q	0.90, t (7.3)	24.2, q	1.39, s	24.0, q	1.38, s
12	24.3, q	1.39, s	25.1, q	1.45, s	25.1, q	1.45, s
13	24.5, q	1.43, s	24.8, q	1.45, s	24.9, q	1.41, s
14	24.9, q	1.42, s	25.0, q	1.63, s	25.1, q	1.63, s
15	25.1, q	1.61, s				
1*'*	104.0, s		104.2, s		105.1, s	
2*'*	153.8, s		153.5, s		153.6, s	
3*'*	103.0, s		103.6, s		103.7, s	
4*'*	159.3, s		159.3, s		159.5, s	
5*'*	100.6, d	6.26, s	100.7, s	6.29, s	100.5, d	6.27, s
6*'*	164.6, s		165.0, s		164.1, s	
7*'*	208.9, s		208.7, s		209.2, s	
8*'*	39.7, d	3.90, sept. (6.8)	46.9, d	3.76, sext. (7.0)	45.5, d	3.89, sext. (6.7)
9*'*	17.7, q	1.25, d (6.7)	18.8, q	1.22, d (7.2)	15.6, q	1.26, d (6.6)
10*'*	20.8, q	1.24, d (7.3)	24.7, t	a 1.96, m	28.6, t	a 1.75, m
				b 1.50, m		b 1.57, m
11*'*			12.1, q	0.98, t (7.4)	11.2, q	0.85, t (7.4)
OH-6*'*		13.30, s		13.54, s		13.13, s

Phloroglucinols **4** and **5** were both assigned shared the molecular formula C_25_H_32_O_6_, supported by HRESIMS [M + H]^+^ peaks at *m*/*z* 429.2275 and 429.2277 (both calcd for C_25_H_33_O_6_, 429.2272), respectively. Further examination of 1D NMR data (Table 3) with that of callistenone B (**14**) [20] revealed a substitution C-1*'*, where the isovaleryl group in **14** was replaced by a 2-methylbutanoyl moiety in both compounds. This structural change was validated by HMBC correlation between Me-9*'* and C-7*'* and ^1^H–^1^H COSY cross-peaks linking H-8*'/*H_2_-10*'* and H_3_-9*'*/H_3_-11*'* (Fig. 2). The relative configurations for H-7 in compounds **4** and **5** were assigned as *α* and *β* configurations, respectively, and the relative configurations for the C-7′ side chains were determined according to further NMR calculations and DP4+ probability analysis (Fig. 3). In light of ECD calculations (Fig. 4), the absolute configuration of phloroglucinol **4** was established as (7*R*,8′*S*), and comparison of experimental ECD spectra of **5** and **4** led to the establishment of absolute configuration for **5** to be (7*S*,8′*S*). Based on spectral and computational evidence, the two structures were characterized as two PPHTDs and designated rhodotomentodimer J and rhodotomentodimer K, respectively.

Phloroglucinols **6** and **7** shared the molecular formula C_26_H_34_O_6_, as indicated by their HRESIMS [M + H]^+^ signals at *m*/*z* 443.2435 and 443.2438 (both calcd for C_26_H_35_O_6_, 443.2428), respectively. Their 1D NMR spectra (Table 4) were highly similar to those of phloroglucinol **3**, suggesting structural similarity to PPHTD congeners. A distinguishing feature was the presence of a 2-methylbutyl moiety at C-1*'*, evidenced by disclosed by HMBC correlations of H_3_-9*'* with C-7*'* and of OH-6*'* with C-1*'* as well as a COSY defined spin system of H_3_-9*'*/H-8*'*/H_2_-10*'*/H_3_-11*'* (Fig. 2). The relative configurations for H-7 in compounds **6** and **7** were randomly assigned as *α* and *β* configurations, respectively, and further NMR calculations and DP4+ probability analysis allowed the establishment of the relative configurations for the C-7′ side chains (Fig. 3). Owing to ECD calculations (Fig. 4), the absolute configurations of phloroglucinols **6** and **7** were established to be (7*R*,8*S*,8′*S*) and (7*S*,8*R*,8′*S*), respectively. Based on these spectroscopic and computational results, phloroglucinols **6** and **7** were identified as two two PPHTDs and designated rhodotomentodimer L and rhodotomentodimer M, respectively.

**Table 4:** ^13^C (150 MHz) and ^1^H (600 MHz) data for **6**–**8** in CDCl_3_

No	**6**		**7**		**8**	
	*δ*_C_	*δ*_H_, mult. (*J* in Hz)	*δ*_C_	*δ*_H_, mult. (*J* in Hz)	*δ*_C_	*δ*_H_, mult. (*J* in Hz)
1	111.6, s		112.7, s		112.6, s	
2	198.4, s		198.1, s		197.6, s	
3	56.2, s		56.2, s		56.2, s	
4	211.8, s		211.7, s		212.2, s	
5	47.4, s		47.2, s		47.2, s	
6	167.9, s		167.7, s		167.6, s	
7	30.5, d	4.42, d (3.2)	30.9, d	4.37, d (2.8)	31.4, d	4.32, d (3.0)
8	42.3, d	1.62, m	42.1, d	1.57, m	41.9, d	1.60, m
9	14.6, q	0.77, d (6.9)	14.7, q	0.75, d (6.8)	15.3, q	0.71, d (6.9)
10	26.6, t	a 1.43, m	26.1, q	a 1.51, m	26.3, t	a 1.45, m
		b 0.92, m		b 1.03, m		b 1.01, m
11	12.4, q	0.92, t (7.3)	12.3, q	0.90 t (7.3)	12.0, q	0.89 t (7.3)
12	25.1, q	1.39, s	24.2, q	1.39, s	24.1, q	1.36, s
13	25.4, q	1.45, s	24.9, q	1.44, s	24.7, q	1.41, s
14	23.7, q	1.44, s	24.6, q	1.41, s	24.6, q	1.41, s
15	25.0, q	1.63, s	25.1, q	1.62, s	25.1, q	1.57, s
1*'*	104.1, s		105.3, s		107.3, s	
2*'*	153.2, s		154.0, s		162.3, s	
3*'*	104.7, s		103.1, s		104.4, s	
4*'*	159.0, s		159.3, s		156.6, s	
5*'*	100.7, d	6.27, s	100.4, d	6.26, s	94.9, d	6.10, s
6*'*	164.9, s		164.0, s		158.5, s	
7*'*	208.7, s		209.3, s		210.8, s	
8*'*	46.8, d	3.76, sext. (6.9)	45.5, d	3.88, sext. (6.6)	46.5, d	3.74, sext. (6.7)
9*'*	18.8, q	1.23, d (7.1)	15.6, q	1.26, d (6.6)	16.6, q	1.18, d (6.8)
10*'*	24.8, t	a 1.96, m	28.6, t	a 1.75, m	26.8, t	a 1.87, m
		b 1.50, m		b 1.56, m		b 1.43, m
11*'*	12.1, q	0.98, t (7.4)	11.2, q	0.85, t (7.5)	12.4, q	0.94, t (7.4)
OH-2*'*						7.51, s
OH-6*'*		13.54, s		13.12, s		12.30, s

Phloroglucinol **8** was assigned the same molecular formula C_26_H_34_O_6_, identical to that of **7**, as indicated by its HRESIMS peak at *m*/*z* 443.2431 [M + H]^+^ (calcd for C_26_H_35_O_6_, 443.2428). Analysis of the ^13^C NMR spectrum (Table 4), supported by HSQC and DEPT data, revealed 26 carbon resonances, including three keto carbonyls, an aromatic ring, four quaternary carbons (two olefinic ones), three methine groups, two methene groups, and eight methyl groups. Comparison of phloroglucinol **8** and rhodotomentodimer D (**10**) [13] indicated the replacement of the isopentyl side chain at C-1 by a 2-methylbutyl group. This substitution was corroborated by a ^1^H–^1^H COSY spin system (Fig. 2) of *δ*_H_ 4.32 (H-7)/1.60 (H-8)/1.45 (H_2_-10a) and 1.01 (H_2_-10b) and 0.71 (H_3_-9)/0.89 (H_3_-11) and HMBC correlations of *δ*_H_ 4.32 (H-7) with *δ*_C_ 104.4 (C-3*'*), 112.6 (C-1), 156.6 (C-4*'*), 162.3 (C-2*'*), 167.6 (C-6), and 197.6 (C-2)*.* The relative configuration of H-7 in compound **8** was stochastically assigned to be *β* orientated, the *β* configuration for the C-8 and C-8*'* ethyl groups were determined by theoretical NMR calculations and DP4+ probability analysis (Fig. 3). Subsequent ECD calculations (Fig. 4) permitted the assignment of the absolute configuration for phloroglucinol** 8** to be (7*S*,8*R*,8′*S*). Hence, phloroglucinol **8** was identified as a PPHTD and named rhodotomentodimer N.

Given that rhodotomentodimer D (**10**) [16], a structurally related analogue, exhibited inhibitory activity against human acetylcholinesterase (*h*AChE), further screening was conducted to assess this activity among related compounds (Table 5). All PPHTDs (**3**‒**14**) demonstrated significant *h*AChE inhibition, with IC_50_ values between 1.04 and 3.35 μM. In contrast, phloroglucinols **1** and **2** were inactive, with IC_50_ values exceeding 40.0 μM.

**Table 5:** *h*AChE inhibitory activities of phloroglucinols **1**–**9** and **11**–**14**

Compounds	IC_50_ ± SD (μM)	Compounds	IC_50_ ± SD (μM)
1	> 40.0	**8**	3.35 ± 0.41
2	> 40.0	**9**	2.86 ± 0.30
3	2.89 ± 0.30	**11**	1.36 ± 0.16
4	2.39 ± 0.25	**12**	1.52 ± 0.14
5	2.46 ± 0.33	**13**	1.04 ± 0.03
6	2.62 ± 0.21	**14**	1.23 ± 0.11
7	2.41 ± 0.22	Galanthamine	1.06 ± 0.05

**Fig. 5:**
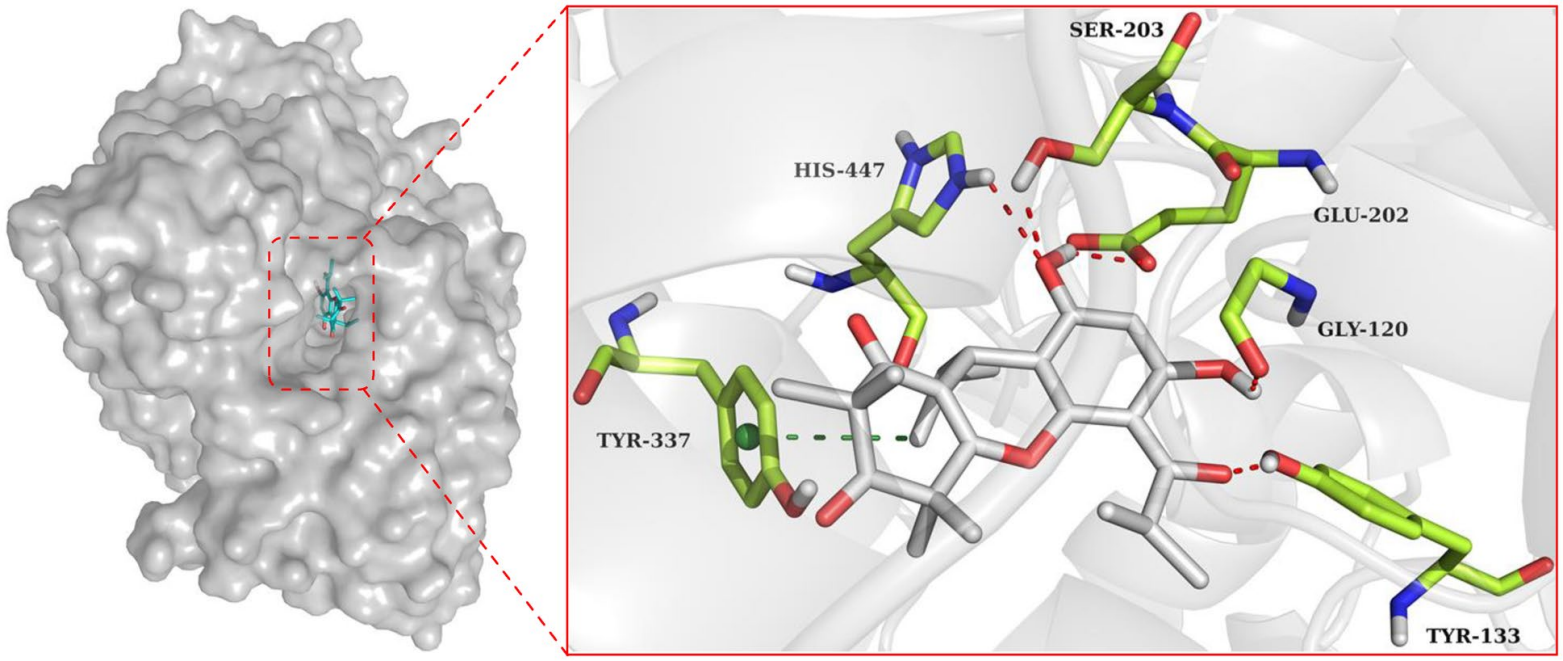
Molecular docking of phloroglucinol **13** with *h*AChE

To gain insights into the binding modes of excellent *h*AChE inhibitor **13**, molecular docking was carried out using the crystal structure of recombinant *h*AChE complex with (‒)-galantamine (PDB code: 4EY6, 2.40 Å resolution) [22]. The docking results indicated that phloroglucinol **13** fits well within the active site pocket of *h*AChE (Fig. 5). Apart from a *π*‒*σ* stacking interaction with Tyr337 (3.61 Å), five hydrogen bonds were observed between phloroglucinol **13** and the residues Gly120 (2.46 Å), Tyr133 (1.67 Å), Glu202 (2.13 Å), Ser203 (2.24 Å), and His447 (2.39 Å).

In light of well-documented antibacterial activity of rhodomyrtone (**12**) [23–26], other phloroglucinol compounds were also assessed for their inhibitory effects on vancomycin-resistant *Enterococci* (VRE). Phloroglucinols **11** and **12** exhibited strong activity (MIC = 1 μg/mL), outperforming ampicillin (AMP, MIC = 2 μg/mL). Similarly, phloroglucinols **8**‒**10** showed comparable potency (MIC = 2 μg/mL). In contrast, phloroglucinols **1**‒**7**, **13**, and **14** were inactive (MIC > 32 μg/mL). These findings suggested that the different connection patterns of PPHTDs can significantly affect their antibacterial properties, thereby offering valuable insights for the design and synthesis of novel antibacterial agents.

## Conclusions

A total of 14 diverse phloroglucinol derivatives (**1**‒**14**), including eight newly identified isolates (**1**‒**8**), were obtained from *R. tomentosa* fruits. The absolute configurations of the side chains in phloroglucinols **3**‒**8** were unambiguously determined through a combination of ECD computations, NMR calculations, and DP4+ probability assessments. In addition, the previously reported phloroglucinol dimers (**9**–**14**) were obtained from *R. tomentosa* fruits for the first time, and several of them represent characteristic and major bioactive constituents of both *R. tomentosa* and *Myrtus communis*. Notably, many of the isolated compounds demonstrated potent *h*AChE inhibitory activities, highlighting their potential as lead candidates for the treatment of neurological disorders, including Alzheimer*'*s disease [27]. Furthermore, several isolates exhibited significant anti-VRE effects, underscoring the therapeutic promise of *R. tomentosa* fruits for combating drug-resistant bacterial infections [28, 29]. Given the limited prior knowledge of the phloroglucinol constituents of *R. tomentosa* fruits, the present findings significantly expand current understanding and provide a scientific basis for the future development of nutraceutical and medicinal food products.

## Experimental procedures

### General experimental procedures

The experimental procedures followed those previously reported in our recent publication [30].

### Plant material

The mature fruits of *R. tomentosa* were collected in Wanning, Hainan province, China, in September 2023. The plant material was authenticated by Xin-Quan Yang (Hainan Branch Institute of Medicinal Plant Development, Chinese Academy of Medical Sciences). A voucher specimen (KIB-Q-202301) has been deposited at the State Key Laboratory of Phytochemistry and Natural Medicines, Kunming Institute of Botany, Chinese Academy of Sciences.

### Extraction and isolation

The air-dried and powdered fruits of *R. tomentosa* (19.5 kg) were extracted thrice with ethanol at room temperature (48 h). The ethanol extracts were concentrated under reduced pressure, to afford a crude residue (1.56 kg), which was partitioned with petroleum ether (PE) and ethyl acetate (EtOAc), yielding PE (331 g) and EtOAc-soluble (282 g) fractions. To eliminate lipoid components, the PE extract was further partitioned with MeOH‒H_2_O (8:2, *v*/*v*), yielding a phloroglucinol enriched moiety (58.0 g). This enriched extract was subjected to silica gel column chromatography with a PE–EtOAc (100:1 → 1:1, *v*/*v*) gradient, yielding seven main fractions (Fr. A‒Fr. G). Fr. A (28.6 g) was further separated on silica gel (PE‒EtOAc, 100:0 → 0:100, *v*/*v*), producing three subfractions (Fr. A_1_–Fr. A_3_). Fr. A_1_ (6.7 g) was purified by an RP-C18 column (MeOH–H_2_O, 70:30 → 100:0, *v*/*v*), producing six subfractions (Fr. A_1.1_–Fr. A_1.6_). Compound **12** (4 mg) was obtained from Fr. A_1.1_ via recrystallization d (Acetone–MeOH, 10:1, *v*/*v*), while **10** (2.8 mg,* t*_R_ = 36 min) was isolated from Fr. A_1.2_ (0.05 g) by semi-preparative HPLC (MeOH–H_2_O, 83:17, *v*/*v*). Fr. B (13 g) was separated by silica gel chromatography (PE–EtOAc, 15:1 → 0:16, *v*/*v*), resulting in seven subfractions (Fr. B_1_–Fr. B_7_). Fr. B_1_ (0.3 g) was further purified via semi-preparative HPLC (MeCN‒H_2_O, 52:48, *v*/*v*) to afford compounds **9** (3.1 mg, *t*_R_ = 33 min) and **13** (10.5 mg, *t*_R_ = 49 min). Fr. C (12 g) underwent RP-C_18_ chromatography (MeOH–H_2_O, 70:30 → 100:0,* v*/*v*), yielding five fractions (Fr. C_1_‒Fr. C_5_). Fr. C_2_ (0.18 g) was further purified by a semi-preparative HPLC (MeCN–H_2_O, 80:20, *v*/*v*), affording compounds **3** (1.4 mg, *t*_R_ = 18 min) and **5** (3.0 mg, *t*_R_ = 15 min). Fr. D (16 g) was fractionated by an RP-C_18_ column (MeOH–H_2_O, 75:25 → 100:0, *v*/*v*), resulting in six subfractions (Fr. D_1_‒Fr. D_6_). Fr. D_2_ (0.92 g) afforded compounds **1** (2.0 mg, *t*_R_ = 35 min) and **8** (2.2 mg, *t*_R_ = 40 min) by semi-preparative HPLC (MeCN–H_2_O, 73:27, *v*/*v*). Likewise, compound **2** (3.2 mg,* t*_R_ = 42 min) was isolated from Fr. D_4_ (0.65 g) by a semi-prep. HPLC (MeCN–H_2_O, 65:35, *v*/*v*). Fr. E (2 g) was separated on Sephadex LH-20 (CHCl_3_–MeOH, 1:1, *v*/*v*), giving three fractions (Fr. E_1_‒Fr. E_3_). Fr. E_1_ (0.39 g) was subjected to semi-preparative HPLC (MeCN–H_2_O, 60:40, *v*/*v*), yielding compounds **4** (3 mg, *t*_R_ = 32 min), **11** (7.2 mg, *t*_R_ = 40 min), **6** (2.6 mg, *t*_R_ = 44 min), **7** (3 mg, *t*_R_ = 42 min), and **14** (7.0 mg, *t*_R_ = 37 min). Chiral separation of compound **2** was achieved using a Daicel CHIRALPAK IC column (*n*-hexane–isopropanol, 95:5 → 85:15, *v*/*v*), furnishing enantiomers (+)-**2** (1.0 mg, *t*_R_ = 12 min) and (−)-**2** (1.0 mg, *t*_R_ = 13 min).

Rhodotomentodione F (**1**): Colorless gum; [*α*] + 28.1 (*c* 0.1, MeOH); UV (MeOH) *λ*_max_ (log *ε*) 268 (3.68) nm; ECD (MeOH) *λ*_max_ (Δ*ε*) 205(–5.36), 241 (+ 1.25), 271 (–4.41), 311 (+ 9.76) nm; ^1^H (600 MHz, CDCl_3_) and ^13^C (150 MHz, CDCl_3_) NMR data, see Table 1; (+)-HRESIMS *m*/*z* 387.2904 [M + H]^+^ (calcd for C_25_H_38_O_3_, 387.2894).

Rhodotomentodimer H (**2**): Colorless gum; [*α*] + 49.0 (*c* 0.10, MeOH) for (+)-**2**; [*α*] –49.0 (*c* 0.10, MeOH) for (–)-**2**; UV (MeOH) *λ*_max_ (log *ε*) 217 (2.68), 272 (2.52) nm; ECD (MeOH) *λ*_max_ (Δ*ε*) 239 (‒4.49), 271 (+4.56), 301 (−5.83), 333 (+ 1.61) nm for (+)-**2**; ECD (MeOH) *λ*_max_ (Δ*ε*) 239 (+4.49), 271 (−4.56), 301 (+ 5.83), 333 (−1.61) nm for (–)-**2**; ^1^H (600 MHz, CDCl_3_) and ^13^C (150 MHz, CDCl_3_) NMR data, see Table 2; ( +)-HRESIMS *m*/*z* 375.1802 [M + H]^+^ (calcd for C_21_H_27_O_6_, 375.1802).

Rhodotomentodimer I (**3**): Colorless gum; [*α*] − 85.4 (*c* 0.10, MeOH); UV (MeOH) *λ*_max_ (log *ε*) 203 (4.08), 216 (4.06), 295 (3.87) nm; ECD (MeOH) *λ*_max_ (Δ*ε*) 209 (+0.34), 222 (‒1.01), 238 (+1.26), 280 (+3.70), 314 (‒4.94) nm; ^1^H (600 MHz, CDCl_3_) and ^13^C (150 MHz, CDCl_3_) NMR data, see Table 3; ( +)-HRESIMS *m*/*z* 429.2279 [M + H]^+^ (calcd for C_25_H_33_O_6_, 429.2272).

Rhodotomentodimer J (**4**): Colorless gum; [*α*] + 149.4 (*c* 0.10, MeOH); UV (MeOH) *λ*_max_ (log *ε*) 203 (4.27), 217 (4.26), 295 (4.10) nm; ECD (MeOH) *λ*_max_ (Δ*ε*) 209 (‒1.19), 221 (+3.65), 237 (−3.38), 280 (−10.08), 313 (+8.51) nm; ^1^H (600 MHz, CDCl_3_) and ^13^C (150 MHz, CDCl_3_) NMR data, see Table 3; (+)-HRESIMS *m*/*z* 429.2275 [M + H]^+^ (calcd for C_25_H_33_O_6_, 429.2272).

Rhodotomentodimer K (**5**): Colorless gum; [*α*] –186.6 (*c* 0.10, MeOH); UV (MeOH) *λ*_max_ (log *ε*) 203 (4.26), 216 (4.27), 294 (4.16) nm; ECD (MeOH) *λ*_max_ (Δ*ε*) 208 (+ 1.49), 221 (‒4.50), 238 (‒3.40), 283 (+ 8.83), 316 (‒10.50) nm; ^1^H (600 MHz, CDCl_3_) and ^13^C (150 MHz, CDCl_3_) NMR data, see Table 3; ( +)-HRESIMS *m*/*z* 429.2277 [M + H]^+^ (calcd for C_25_H_33_O_6_, 429.2272).

Rhodotomentodimer L (**6**): Colorless gum; [*α*] + 83.7 (*c* 0.10, MeOH); UV (MeOH) *λ*_max_ (log *ε*) 203 (4.10), 217 (4.10), 295 (3.98) nm; ECD (MeOH) *λ*_max_ (Δ*ε*) 209 (‒0.79), 221 (+ 2.42), 237 (‒2.24), 278 (‒4.85), 313 (+ 5.65) nm; ^1^H (600 MHz, CDCl_3_) and ^13^C (150 MHz, CDCl_3_) NMR data, see Table 4; (+)-HRESIMS *m*/*z* 443.2435 [M + H]^+^ (calcd for C_26_H_35_O_6_, 443.2428).

Rhodotomentodimer M (**7**): Colorless gum; [*α*] ‒85.4 (*c* 0.10, MeOH); UV (MeOH) *λ*_max_ (log *ε*) 205 (4.25), 218 (4.27), 297 (4.15) nm; ECD (MeOH) *λ*_max_ (Δ*ε*) 209 (+ 1.22), 222 (–2.88), 238 (+ 2.69), 283 (+ 7.78), 316 (–9.79) nm; ^1^H (600 MHz, CDCl_3_) and ^13^C (150 MHz, CDCl_3_) NMR data, see Table 4; ( +)-HRESIMS *m*/*z* 443.2438 [M + H]^+^ (calcd for C_26_H_35_O_6_, 443.2428).

Rhodotomentodimer N (**8**): Colorless gum; [*α*] ‒60.2 (*c* 0.10, MeOH); UV (MeOH) *λ*_max_ (log *ε*) 202 (4.02), 225 (3.99), 263 (3.73), 301 (3.88) nm; ECD (MeOH) *λ*_max_ (Δ*ε*) 207 (+ 0.20), 222 (‒2.94), 262 (+ 11.13), 297 (‒4.81) nm; ^1^H (600 MHz, CDCl_3_) and ^13^C (150 MHz, CDCl_3_) NMR data, see Table 4; ( +)-HRESIMS *m*/*z* 443.2431 [M + H]^+^ (calcd for C_26_H_35_O_6_, 443.2428).

### ECD and NMR calculations

ECD and NMR computations were carried out based on previously reported methods using the Gaussian 16 software package [14]. Three-dimensional molecular structures were constructed based on ROESY spectra (for compound **1)** or randomly generated (for compounds **2–8**), followed by conformational analysis using CONFLEX with the MMFF94S force field. ECD and NMR chemical shift calculations were conducted at the B3LYP/6–311++ G(2d,p) and mPW1PW91/6–31+ G(d,p) levels, respectively. The DP4+ probability analyses were accomplished utilizing a published computational template [31].

### Human AChE inhibitory assay

*h*AChE inhibition assays were performed according to a reported protocol, with galantamine as the positive control [32]. All assays were performed in triplicate. Compounds with IC_50_ values exceeding 40 μM were considered inactive.

### Anti-VRE assay

As described in earlier studies, the antibacterial activities against vancomycin-resistant *Enterococci* (VRE) were achieved by a standard broth microdilution method in 96-well plates [33]. Ampicillin (AMP) was a positive control, and all tests were performed three times.

### Molecular docking

Molecular docking simulations were performed utilizing the AutoDock software suit [34], following established protocols. The docking complexes of phloroglucinol **13** with *h*AChE (PDB ID: 4EY6) were generated, and the resulting binding poses were visualized utilizing the PyMOL Molecular Graphics System.

## Supplementary Information

Supplementary material 1.

## Data Availability

The datasets used or analyzed during the current study are available from the corresponding author on reasonable request.
