# Supplementary material for: Diverse phloroglucinols with hAChE inhibitory and anti-VRE effects from Rhodomyrtus tomentosa fruits
Source: Nat Prod Bioprospect. 2026 Jan 8;16(1):4. doi: 10.1007/s13659-025-00557-0 (PMC12779811; doi:10.1007/s13659-025-00557-0)
Supplement: Supplementary file 1 — Supplementary material 1. [file 13659_2025_557_MOESM1_ESM.pdf]

Supporting Information for

Diverse phloroglucinols with *h*AChE inhibitory and anti-VRE effects from *Rhodomyrtus tomentosa* fruits

Ling-Yun Chen [1,2,#], Mu-Yuan Yu [2,#], E-E Luo [2,3,#], Wen-Ying Zong [2,3], Shu-Mei Lei [2,3], Yu Pan [2,3], Ai-Chun Lu [2,3], Cheng-Qin Liang [1,*], Xu-Jie Qin [2,3,*]

[1] *College of Pharmacy, Guilin Medical University, Guilin, 541199, P.R. China*

[2] *State Key Laboratory of Phytochemistry and Natural Medicines, Kunming Institute of Botany, Chinese Academy of Sciences, Kunming, 650201, P.R. China*

[3] *University of Chinese Academy of Sciences, Beijing, 100049, P.R. China*

Corresponding authors: qinxujie@mail.kib.ac.cn (X.-J. Qin), cqliang@glmc.edu.cn (C.-Q. Liang).

[#] These authors contributed equally.

Contents of Supporting Information

No.	Contents	Page
1.	Figures S1–S6. NMR spectra of **1** in $CDCl_3$	1–3
2	Figure S7. HRESIMS spectrum of **1**	4
3	Figures S8–S11. NMR spectra of **2** in $CDCl_3$	6
4	Figure S12. HRESIMS spectrum of **2**	6
5	Figures S13–S17. NMR spectra of **3** in $CDCl_3$	7–9
6	Figure S18. HRESIMS spectrum of **3**	9
7	Figures S19–S23. NMR spectra of **4** in $CDCl_3$	10–12
8	Figure S24. HRESIMS spectrum of **4**	12
9	Figures S25–S29. NMR spectra of **5** in $CDCl_3$	13–15
10	Figure S30. HRESIMS spectrum of **5**	15
11	Figures S31–S35. NMR spectra of **6** in $CDCl_3$	16–18
12	Figure S36. HRESIMS spectrum of of **6**	18
13	Figures S37–S41. NMR spectra of **7** in $CDCl_3$	19–21
14	Figure S42. HREIMS spectrum of **7**	21
15	Figures S43–S47. NMR spectra of **8** in $CDCl_3$	22–24
16	Figure S48. HREIMS spectrum of **8**	24
17	ECD calculated data for **1**	25
18	ECD calculated data for **2**	26
19	ECD calculated data for **3**	27
20	ECD calculated data for **4**	28–28
21	ECD calculated data for **6**	30–31
22	ECD calculated data for **7**	32
23	ECD calculated data for **8**	33
24	^{13}C NMR calculated data for **3**	34–35
25	^{13}C NMR calculated data for **4**	36–37
26	^{13}C NMR calculated data for **5**	38–39
27	^{13}C NMR calculated data for **6**	40–43
28	^{13}C NMR calculated data for **7**	44–47
29	^{13}C NMR calculated data for **8**	48–51
30	DP4+ analyses of **4–8**	52–57

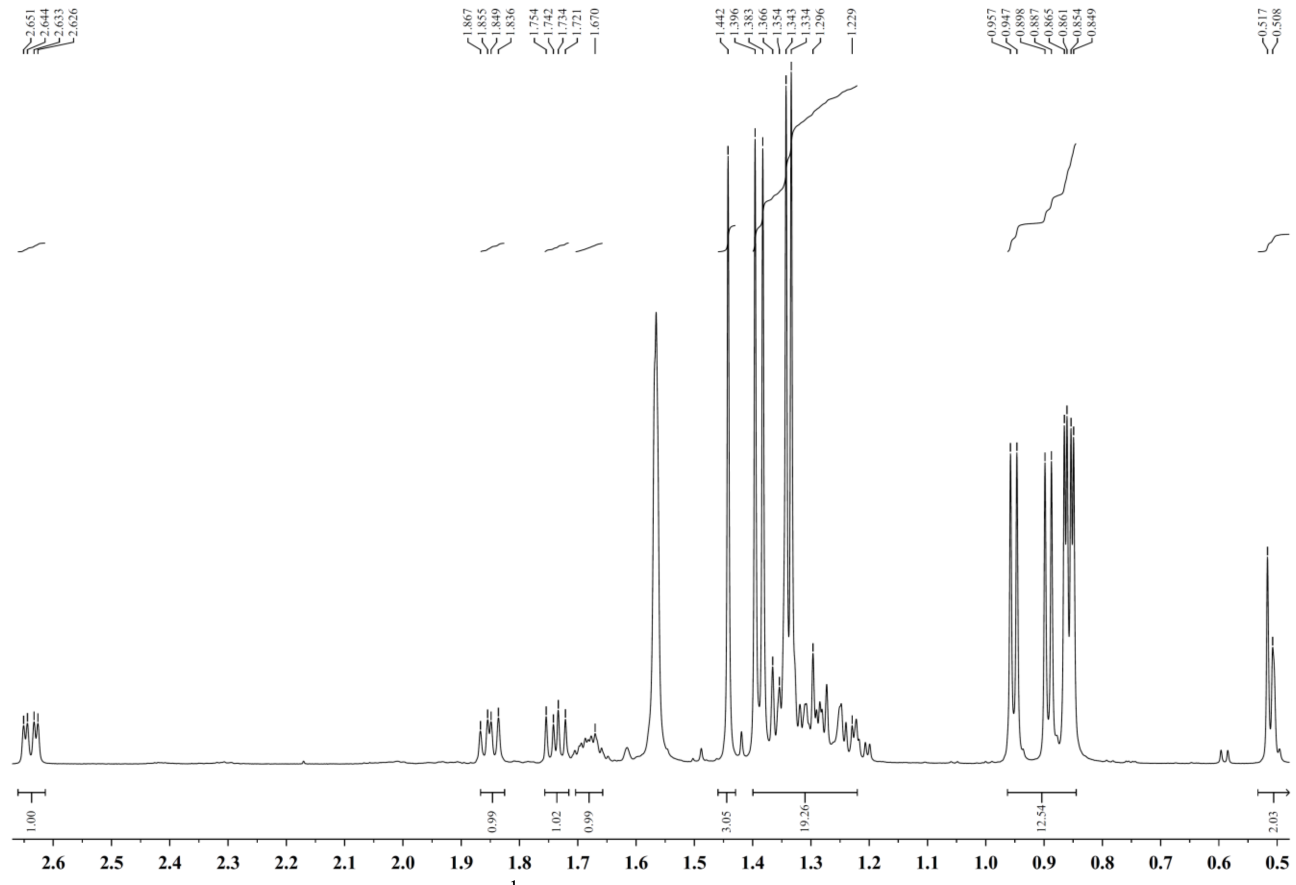

Figure S1. ^{1}H NMR spectrum of **1** in $CDCl_3$

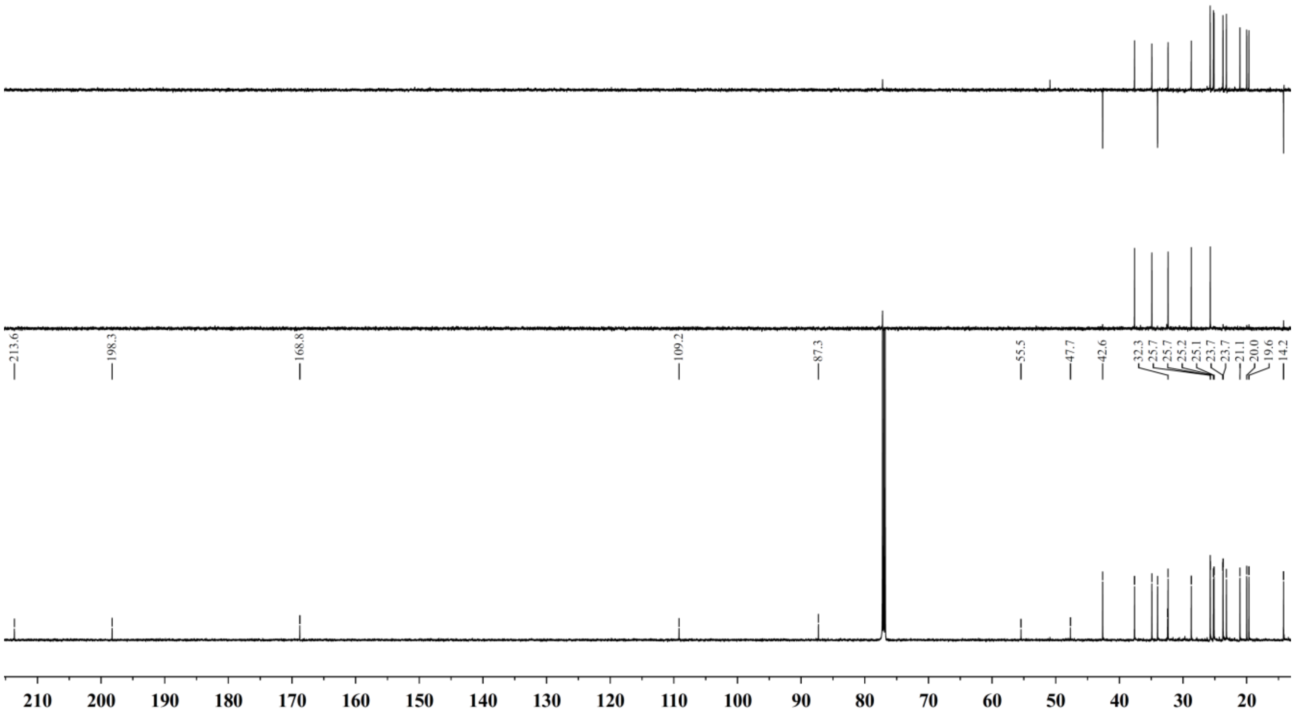

Figure S2. ^{13}C NMR spectrum of **1** in $CDCl_3$

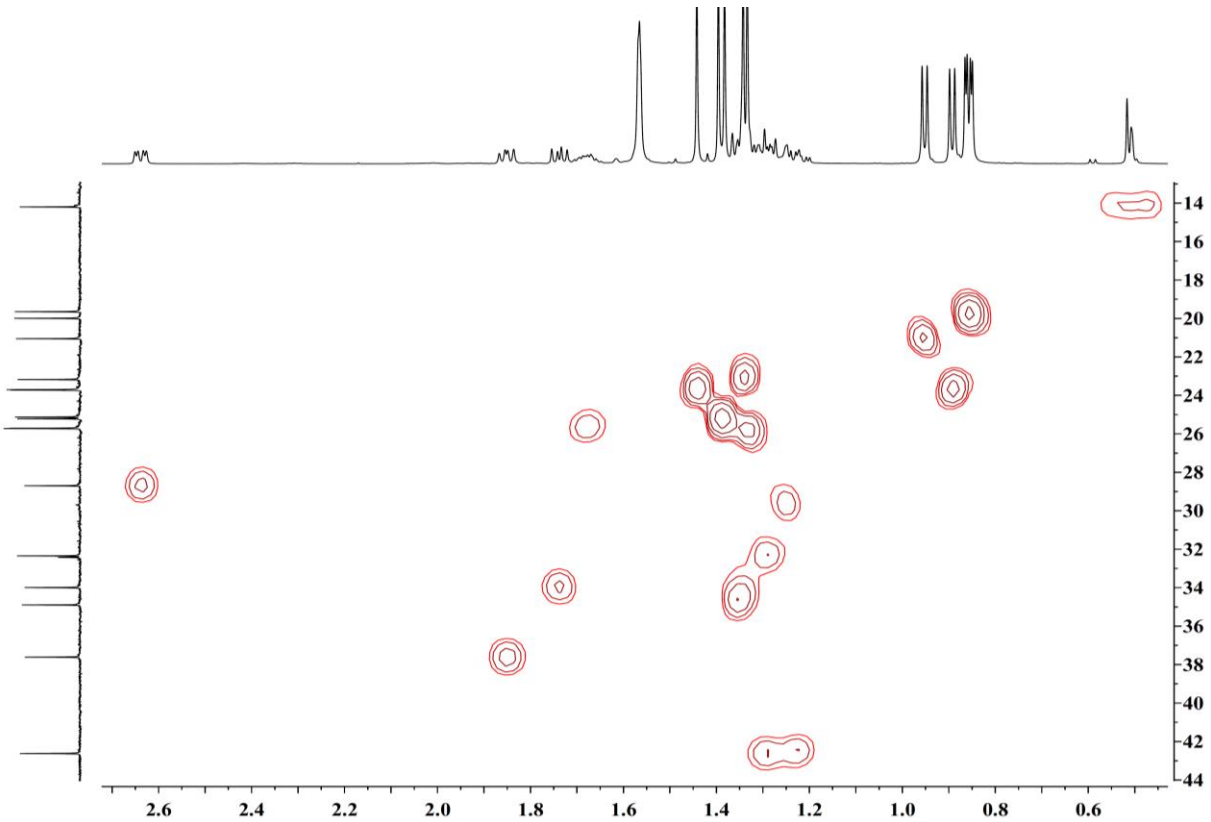

Figure S3. HSQC spectrum of **1**

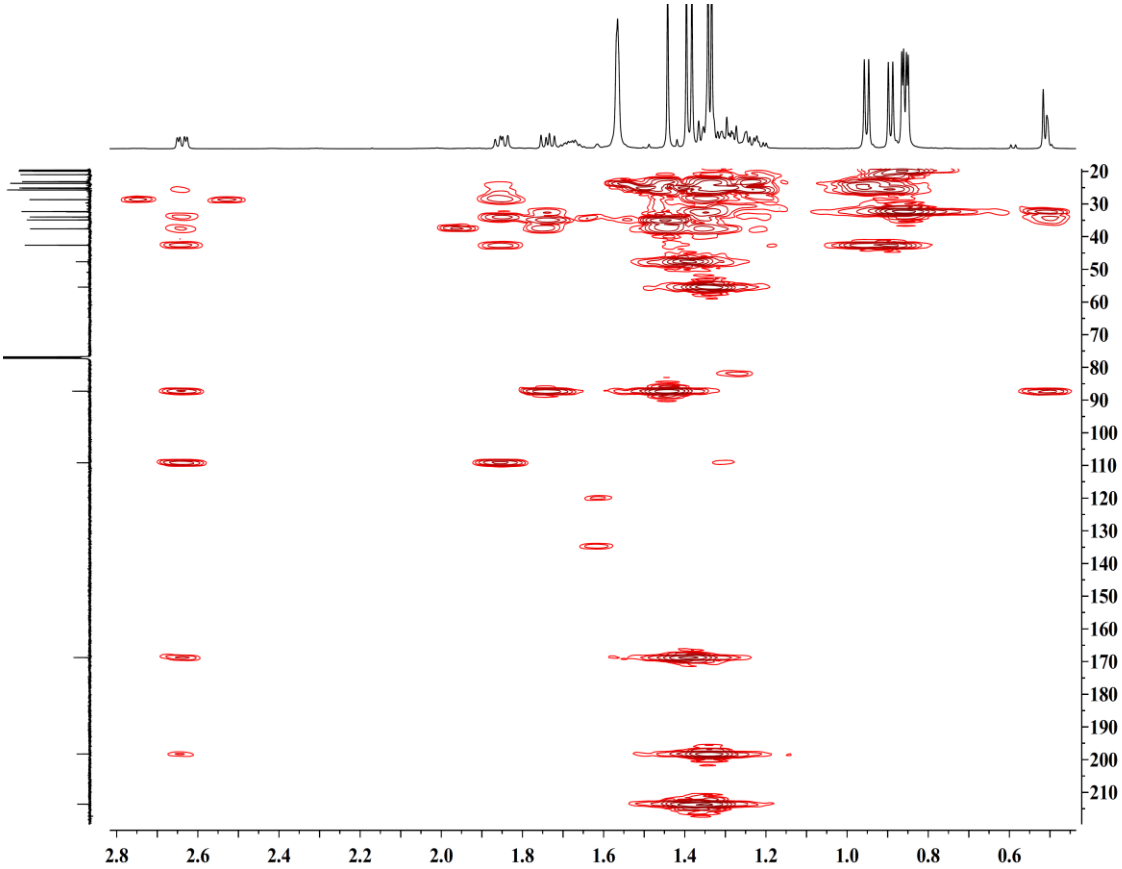

Figure S4. HMBC spectrum of **1**

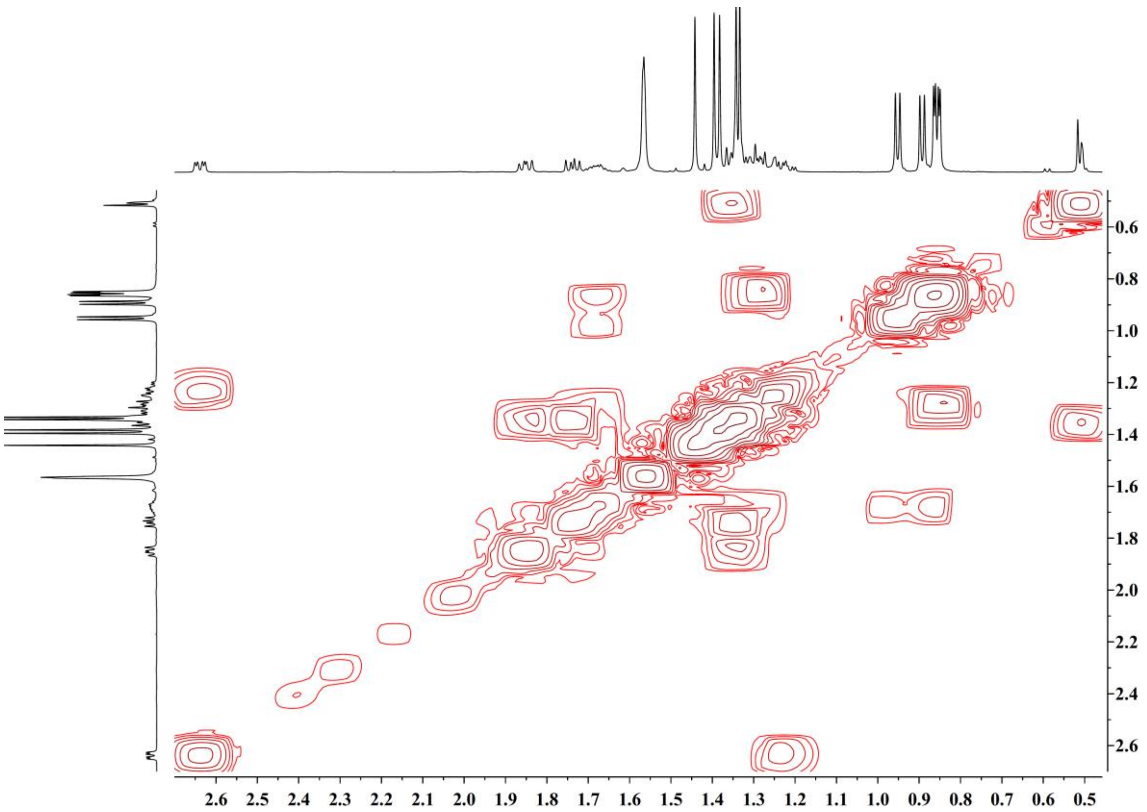

Figure S5. ^{1}H–^{1}H COSY spectrum of **1**

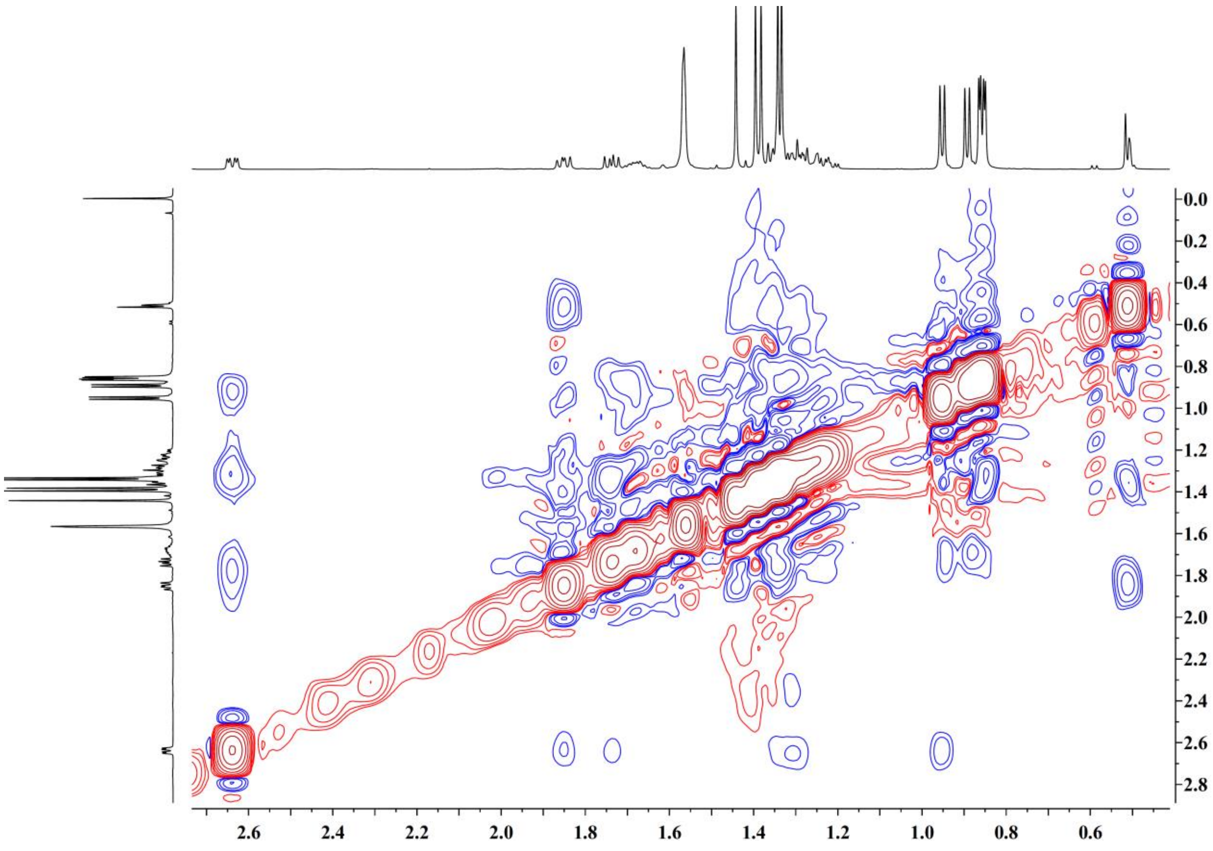

Figure S6. ROESY spectrum of **1**

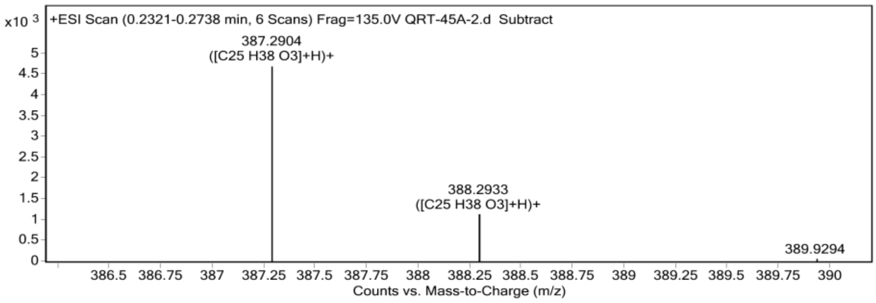

Peak List

m/z	z	Abund	Formula	Ion
274.2748	1	1492.13		
387.2904	1	4698.35	C25 H38 O3	(M+H)+
388.2933	1	1148.71	C25 H38 O3	(M+H)+
409.2724	1	2564.75		
410.2759	1	732.66		
450.2984	1	892.05		
501.3208	1	690.33		
685.4414	1	879.06		
795.5563	1	2963.04		
796.5584	1	1358.58		

Formula Calculator Element Limits

Element	Min	Max
C	3	60
H	0	100
O	0	20

Formula Calculator Results

Formula	CalculatedMass	CalculatedMz	Mz	Diff. (mDa)	Diff. (ppm)	DBE
C25 H38 O3	386.2821	387.2894	387.2904	-1.00	-2.58	7.0000

Figure S7. HREIMS spectrum of **1**

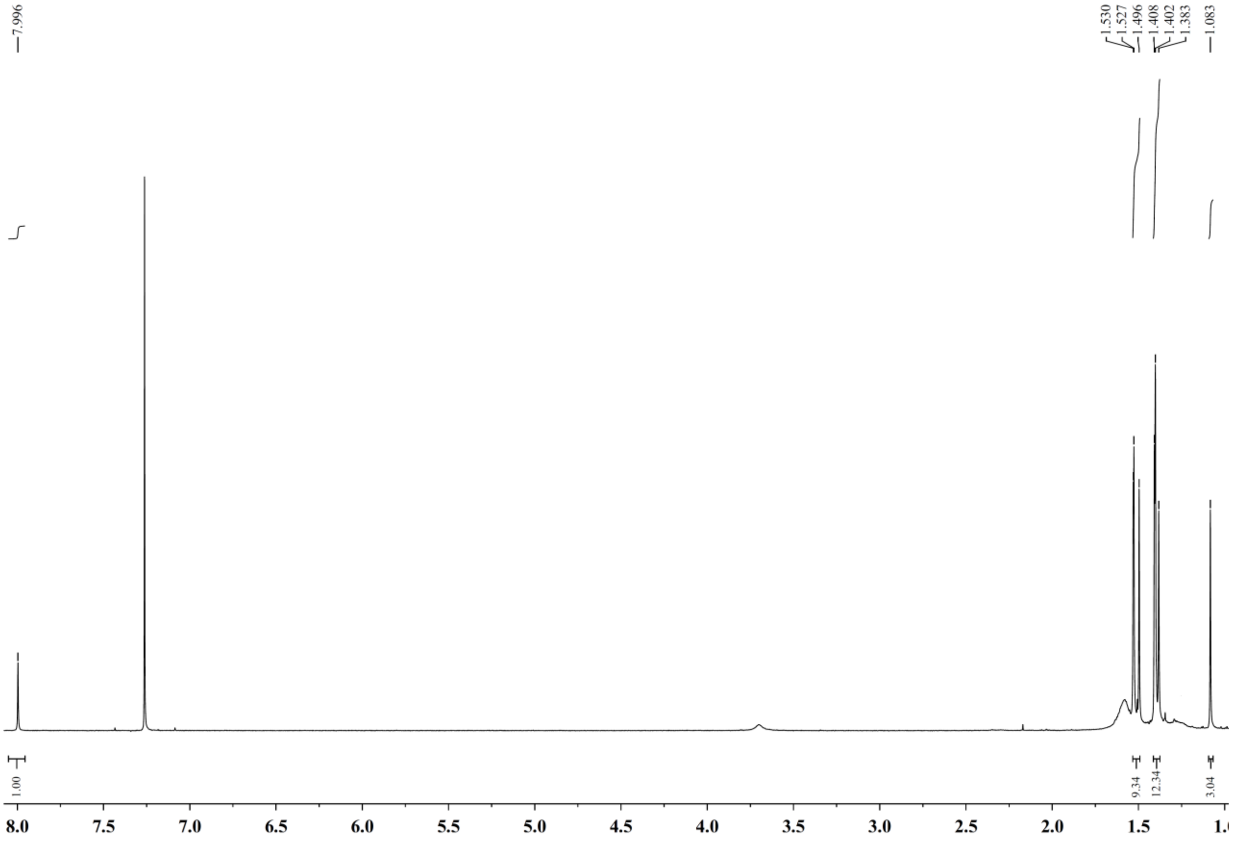

Figure S8. ^{1}H NMR spectrum of **2** in $CDCl_3$

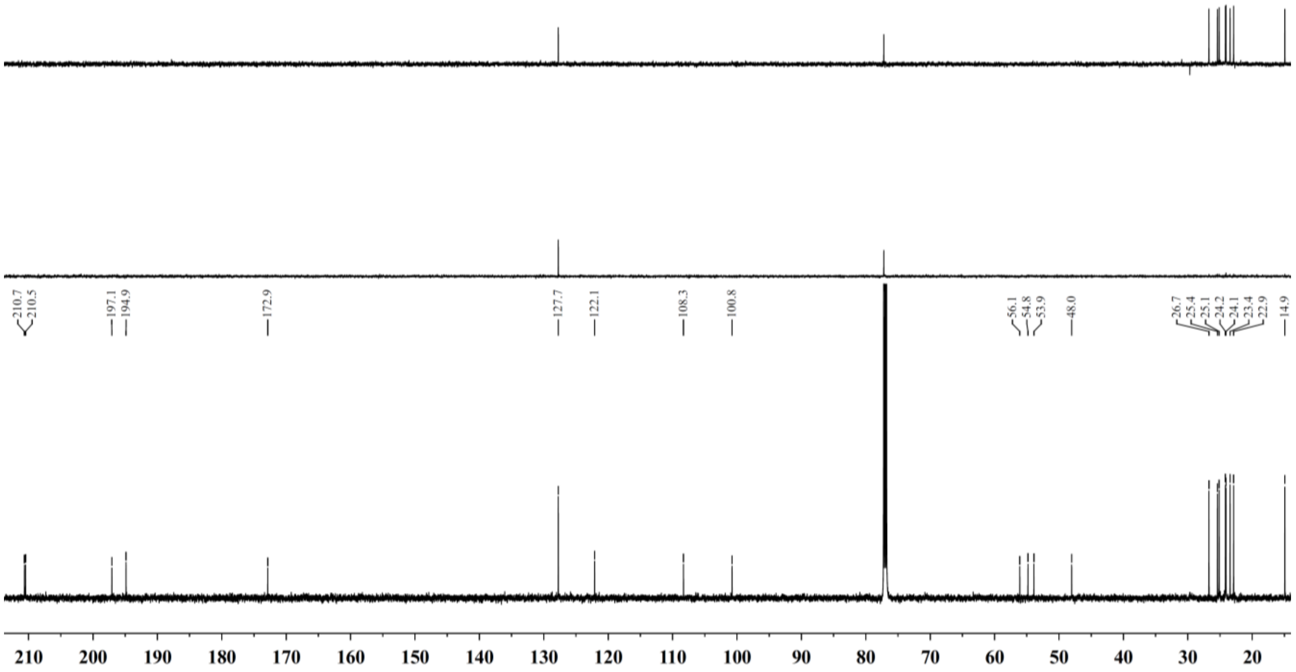

Figure S9. ^{13}C NMR spectrum of **2** in $CDCl_3$

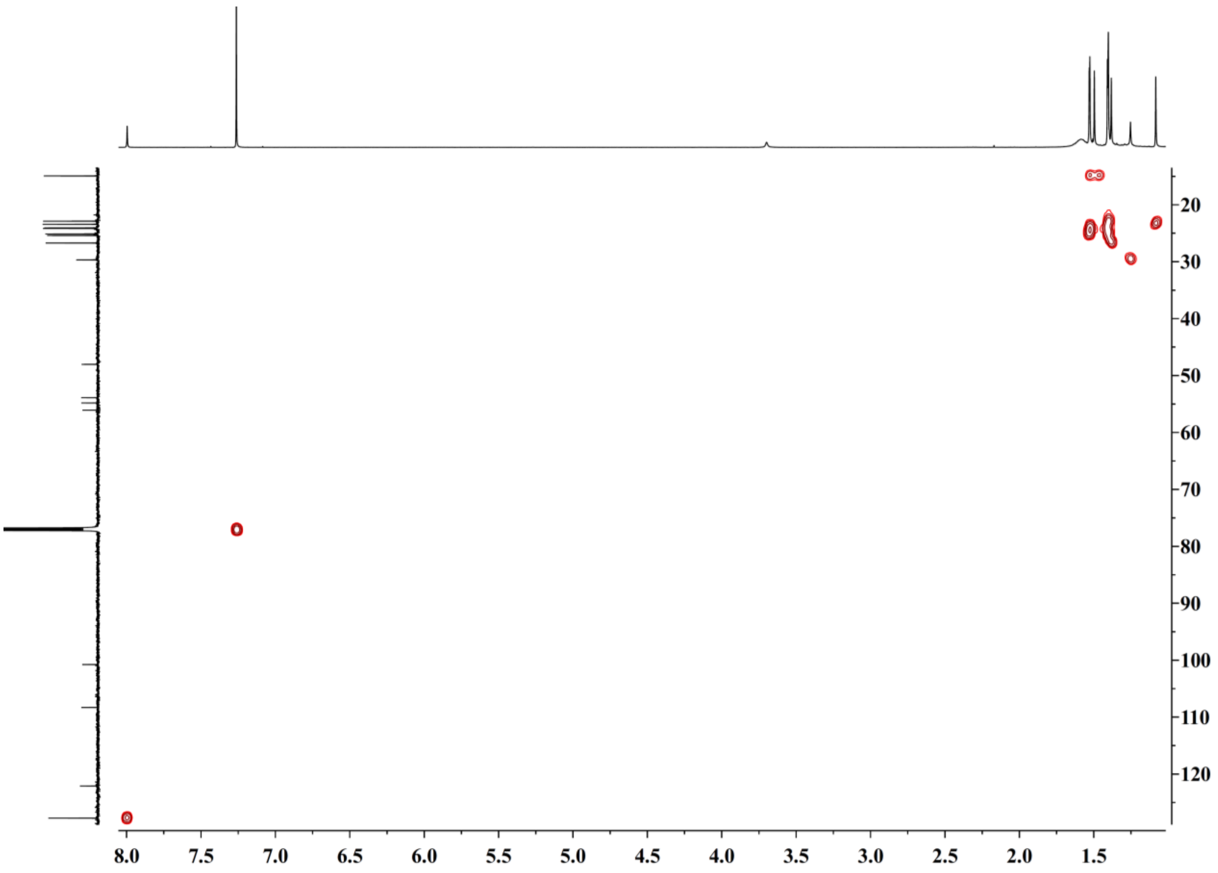

Figure S10. HSQC spectrum of **2**

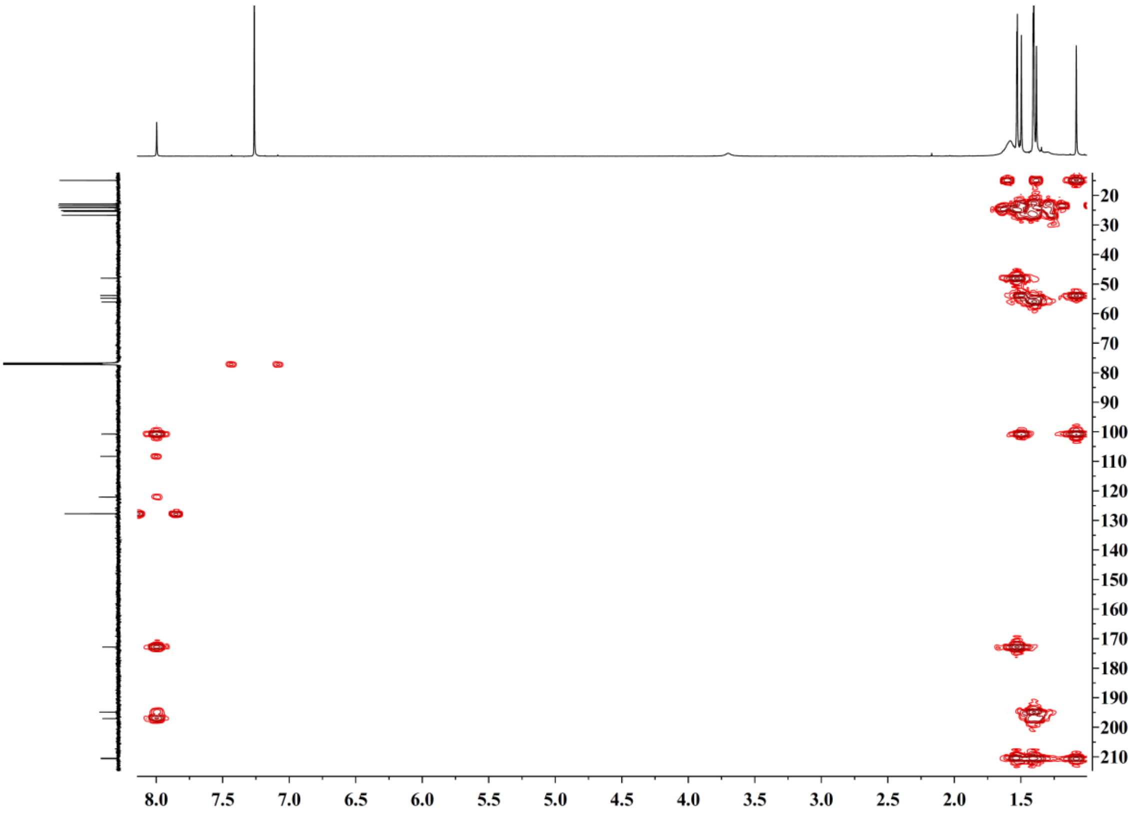

Figure S11. HMBC spectrum of **2**

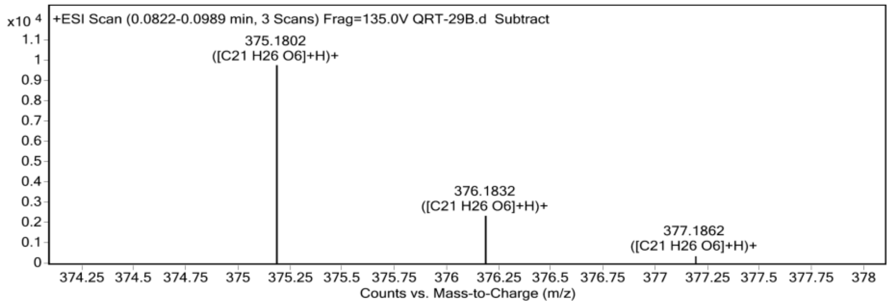

Peak List

m/z	z	Abund	Formula	Ion
274.2746	1	4317.24		
357.1697	1	1966.88		
375.1802	1	9750.97	C21 H26 O6	(M+H)+
376.1832	1	2365.27	C21 H26 O6	(M+H)+
429.2276	1	3849.88		
443.243	1	4294.64		
502.3165	1	1961.75		
507.2731	1	1667.35		
510.2489	1	8310.66		
511.2513	1	2762.57		

Formula Calculator Element Limits

Element	Min	Max
C	3	60
H	0	100
O	0	20

Formula Calculator Results

Formula	CalculatedMass	CalculatedMz	Mz	Diff. (mDa)	Diff. (ppm)	DBE
C21 H26 O6	374.1729	375.1802	375.1802	0.00	0.00	9.0000

Figure S12. HREIMS spectrum of **2**

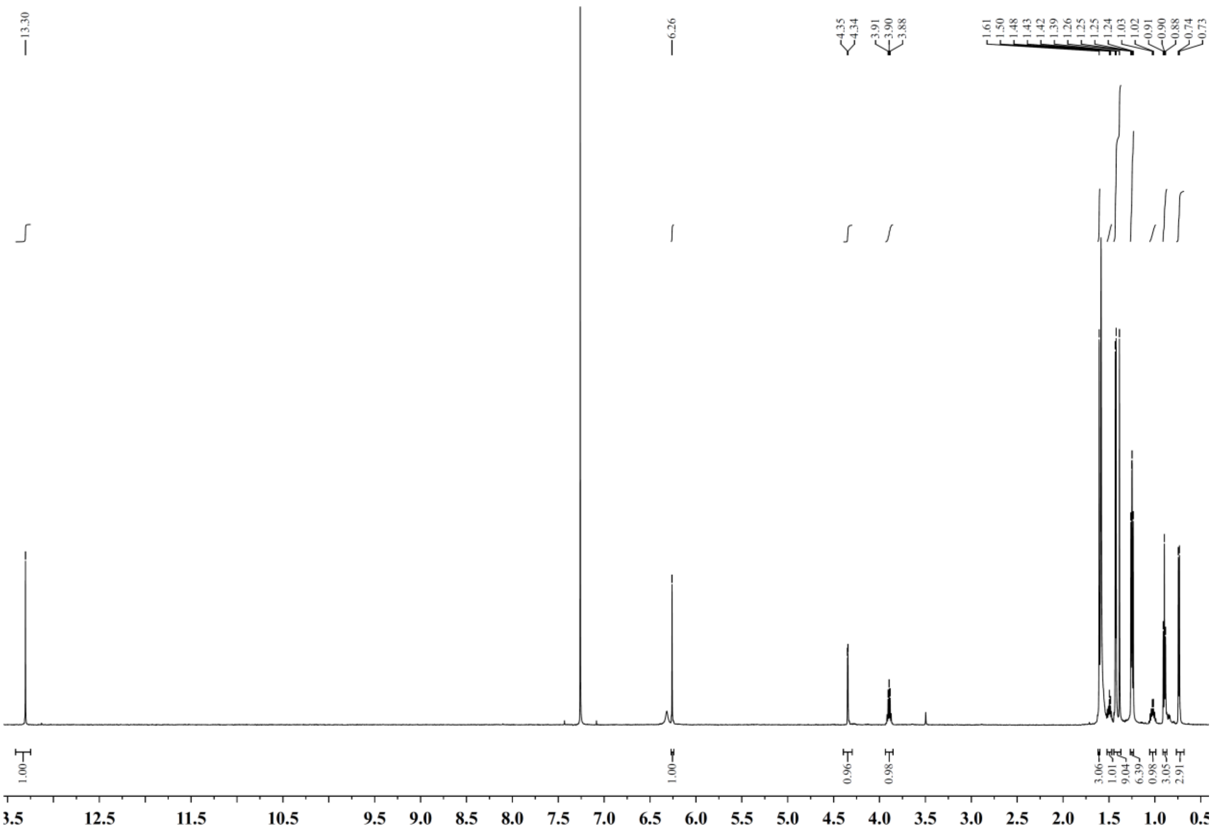

Figure S13. ^{1}H NMR spectrum of **3**

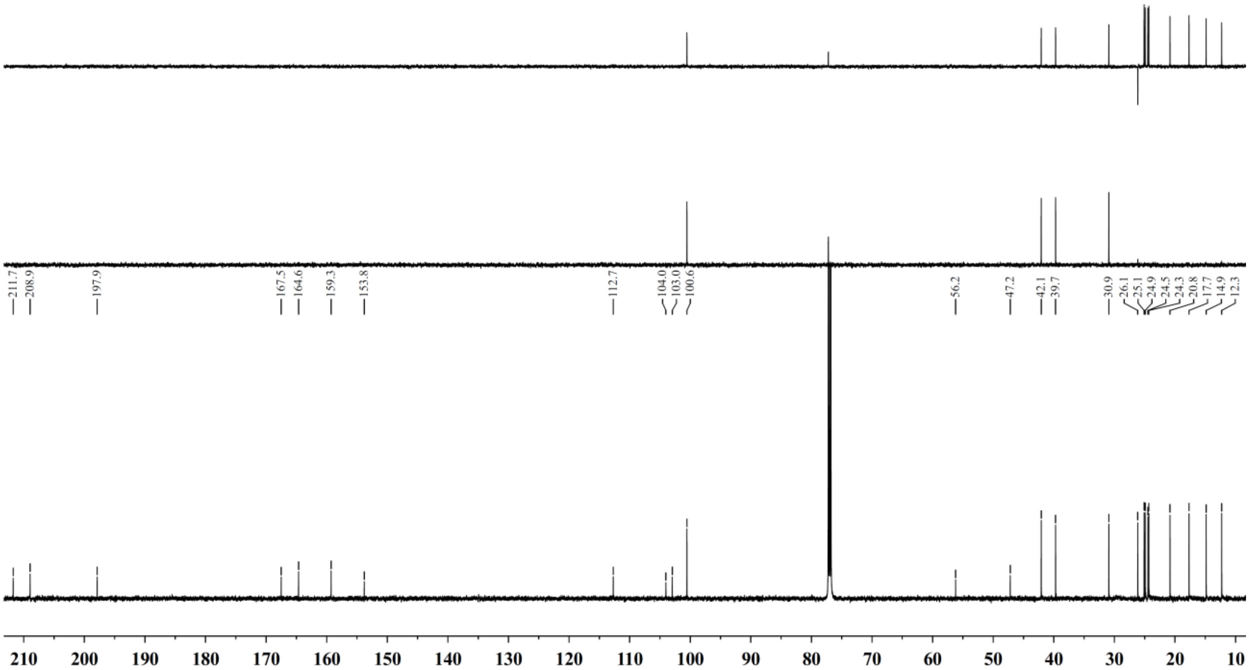

Figure S14. ^{13}C NMR spectrum of **3**

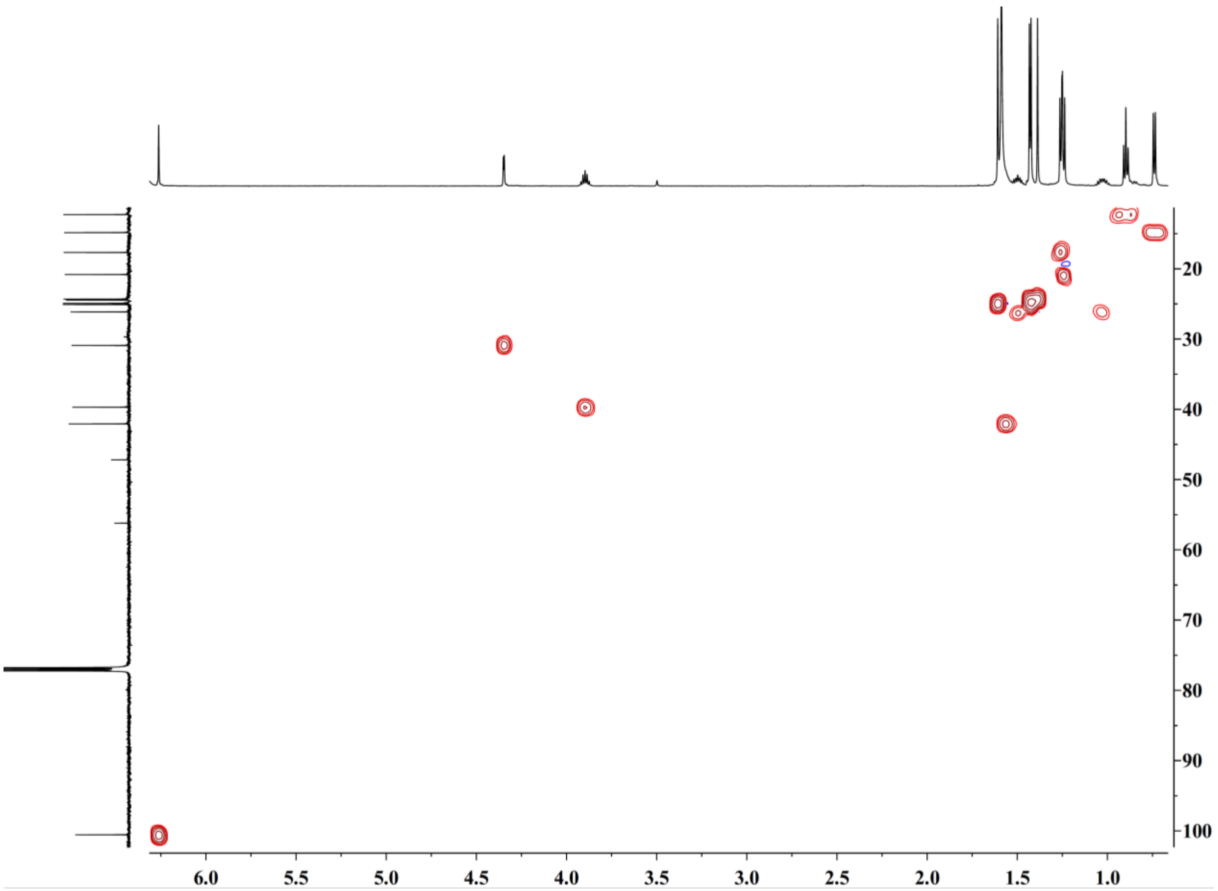

Figure S15. HSQC spectrum of **3**

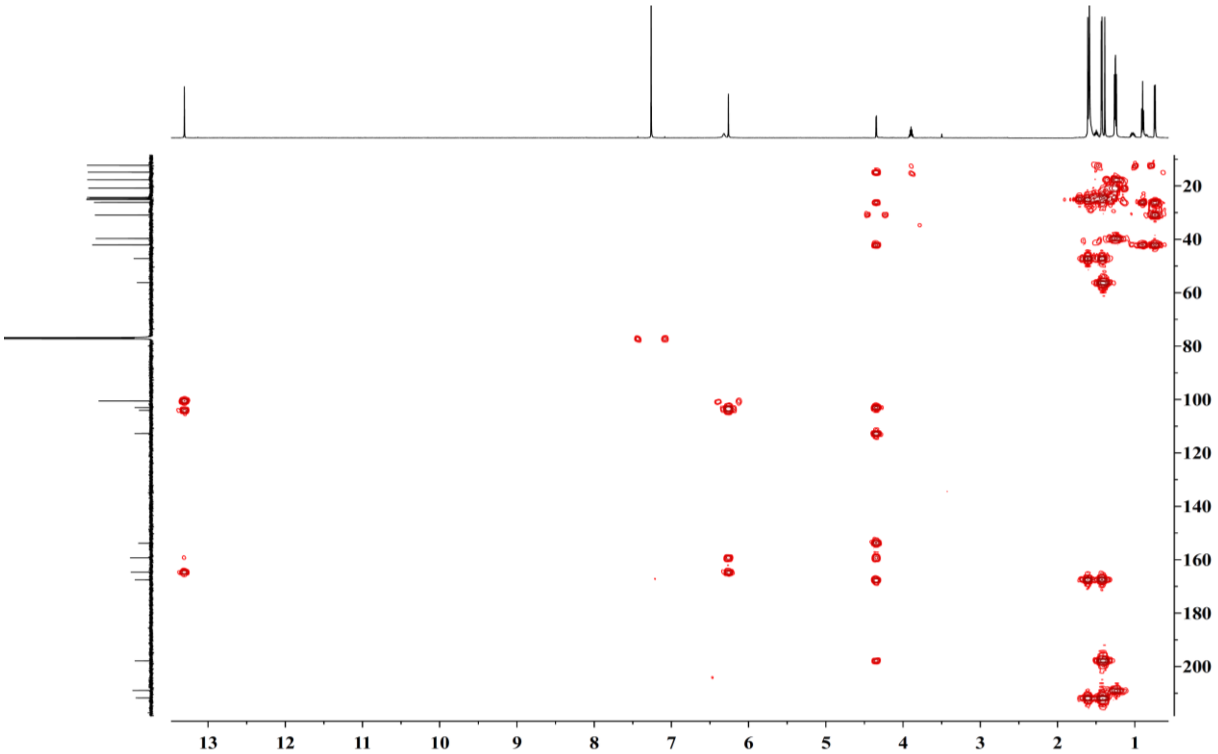

Figure S16. HMBC spectrum of **3**

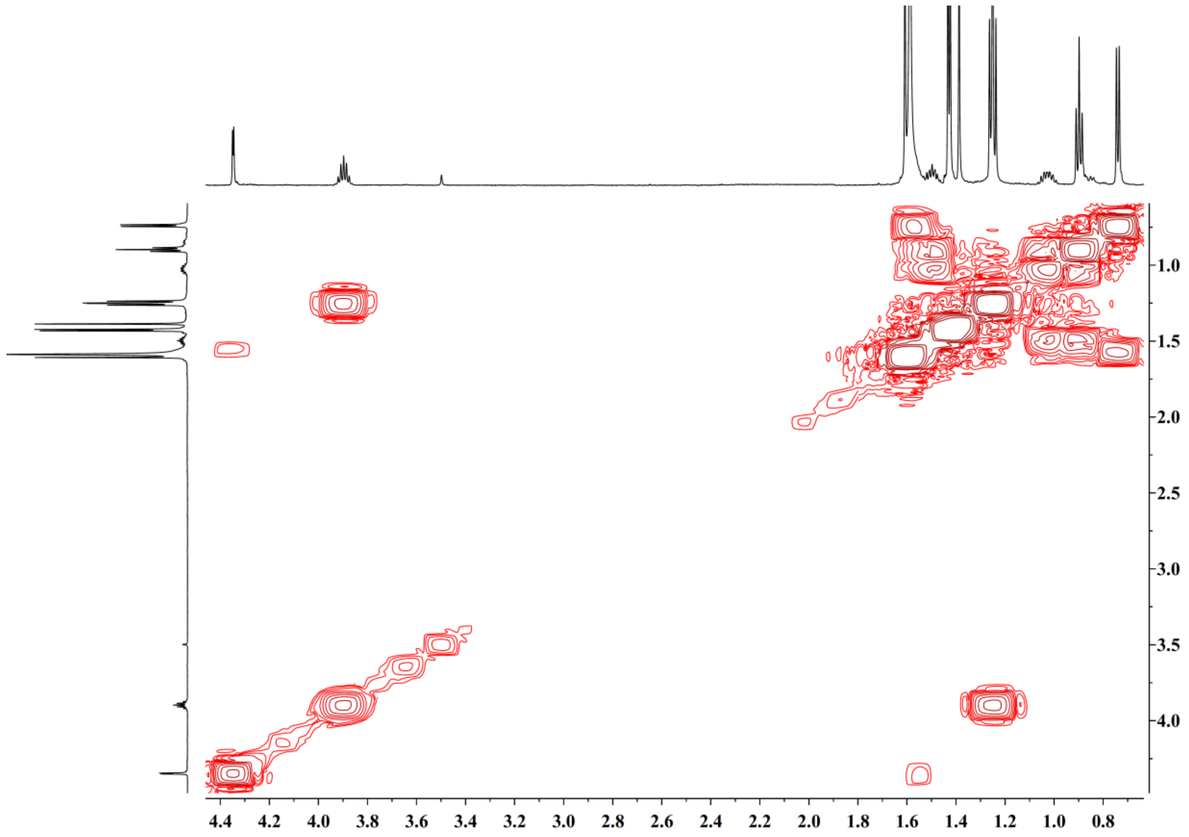

Figure S17. ^{1}H–^{1}H COSY spectrum of **3**

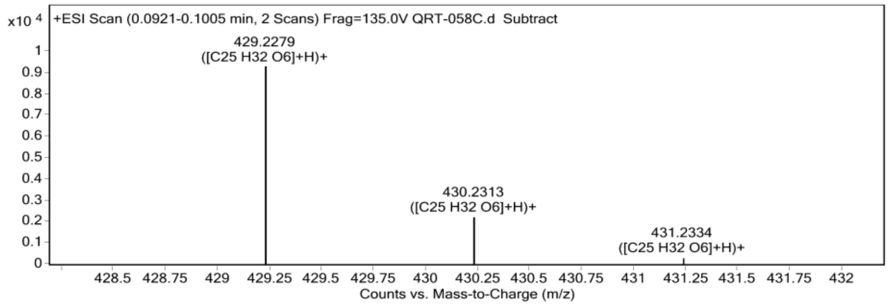

Peak List

m/z	z	Abund	Formula	Ion
81.9378	1	616.18		
102.1279	1	1032		
116.0709	1	599.01		
122.9641	1	1834.35		
141.9586	1	1181.76		
219.1882	1	628.51		
274.2758	1	848.58		
279.0929	1	778.97		
429.2279	1	9311.14	C25 H32 O6	(M+H)+
430.2313	1	2243.55	C25 H32 O6	(M+H)+

Formula Calculator Element Limits

Element	Min	Max
C	3	60
H	0	100
O	0	20
N	0	5

Formula Calculator Results

Formula	CalculatedMass	CalculatedMz	Mz	Diff. (mDa)	Diff. (ppm)	DBE
C25 H32 O6	428.2199	429.2272	429.2279	-0.70	-1.63	10.0000

Figure S18. HRESIMS spectrum of **3**

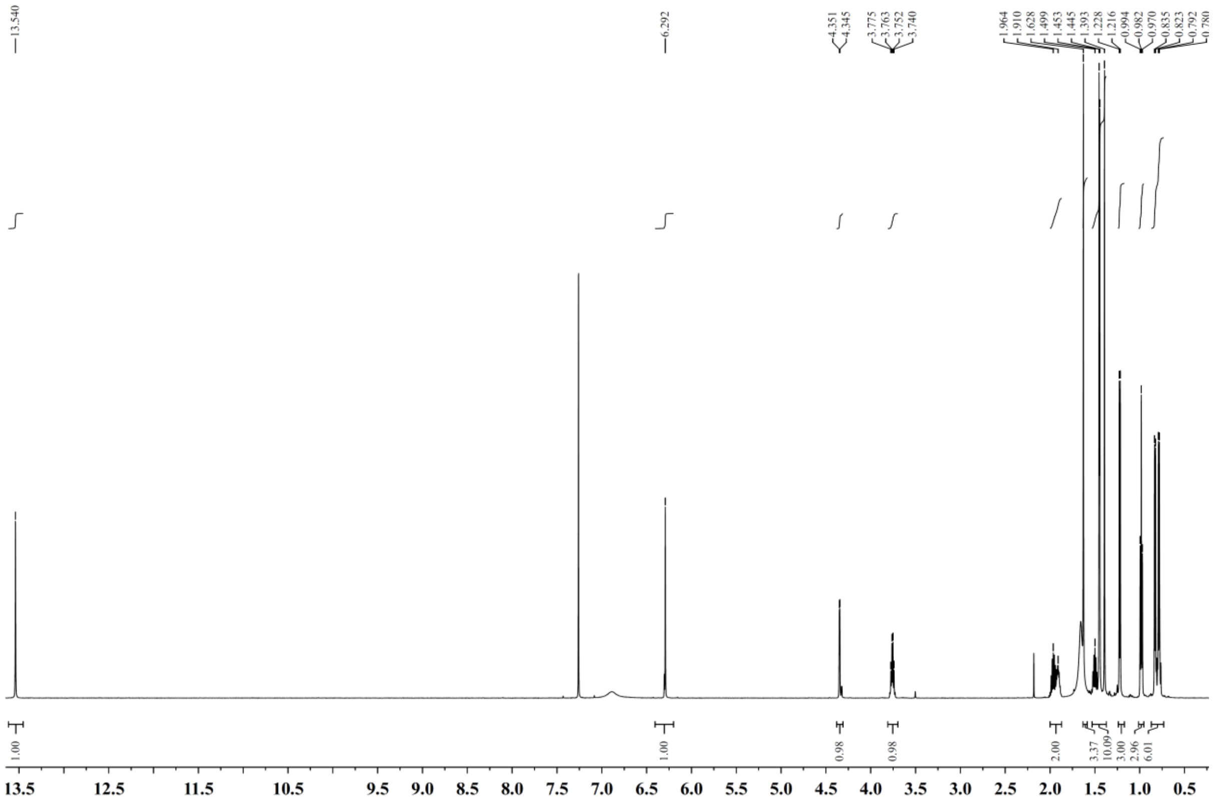

Figure S19. ^{1}H NMR spectrum of **4**

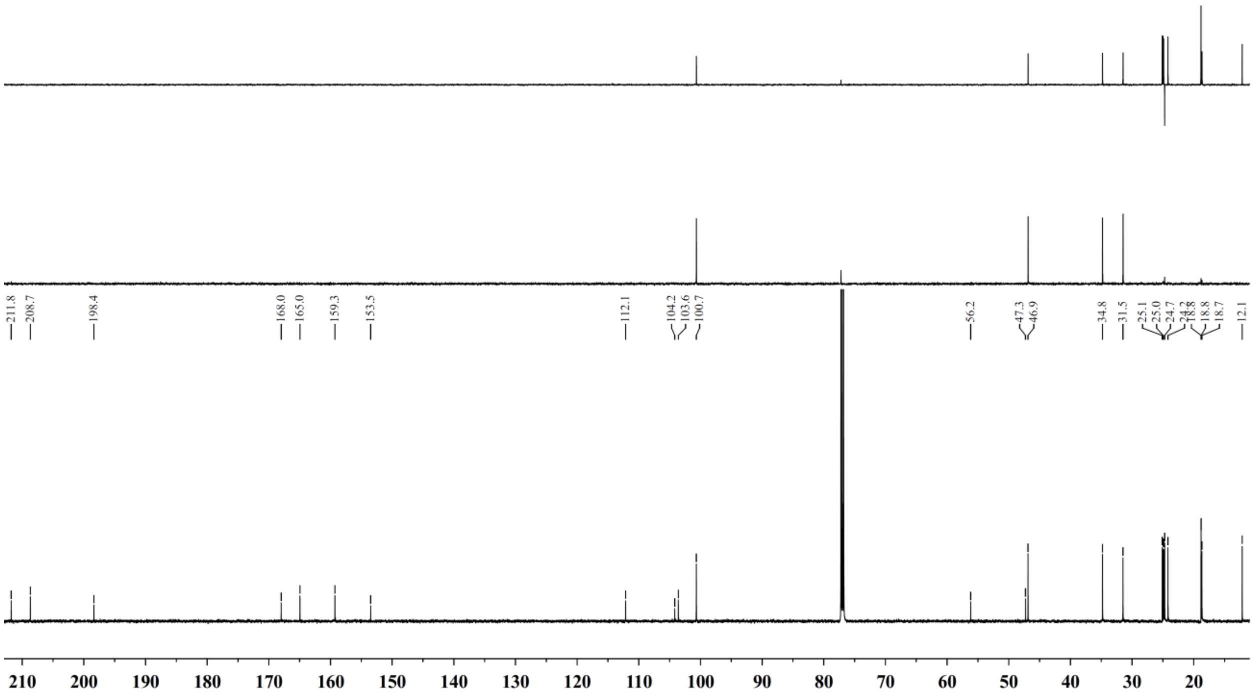

Figure S20. ^{13}C NMR spectrum of **4**

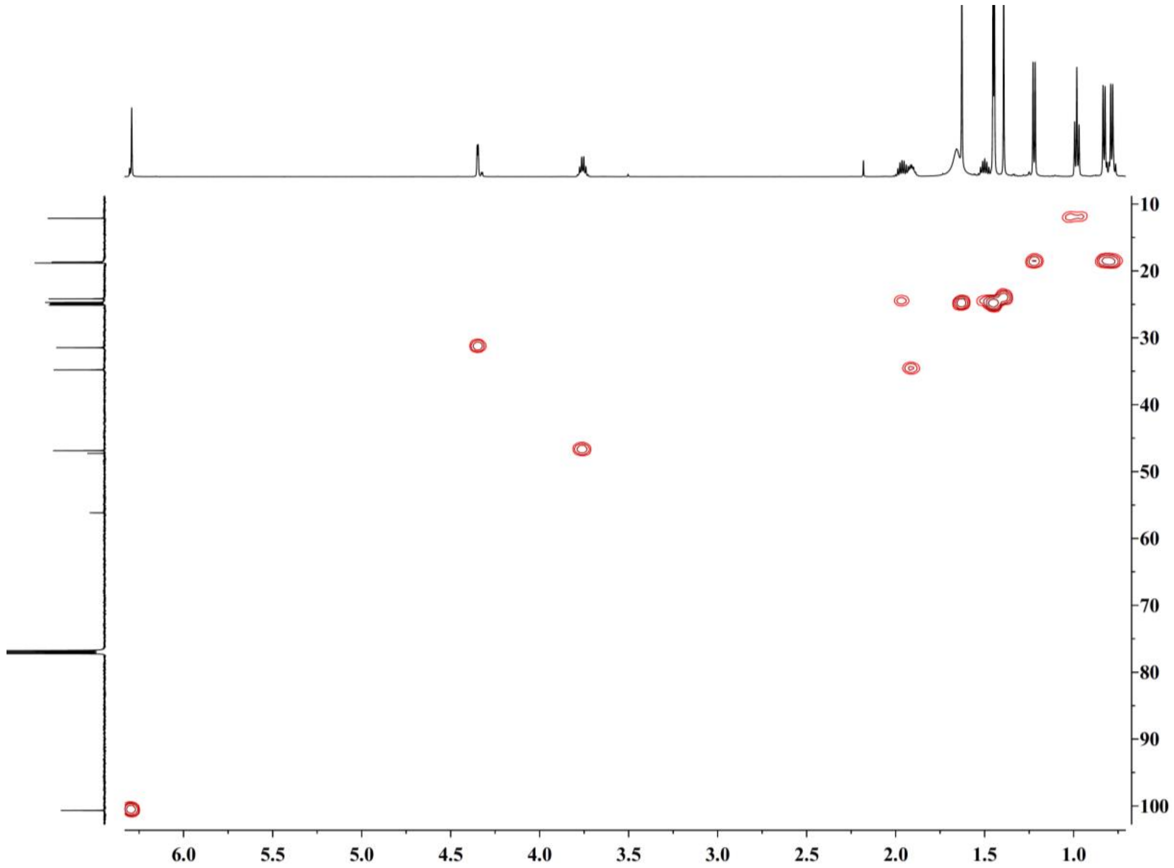

Figure S21. HSQC spectrum of **4**

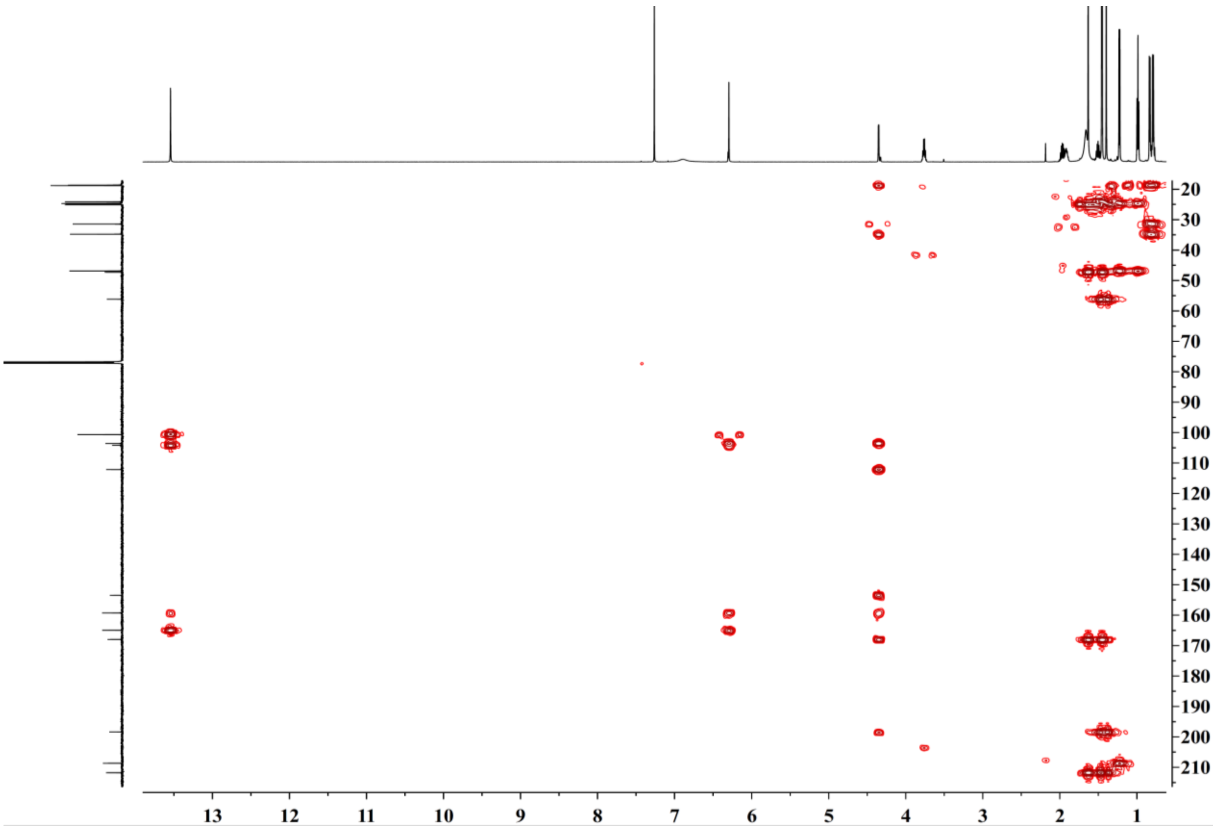

Figure S22. HMBC spectrum of **4**

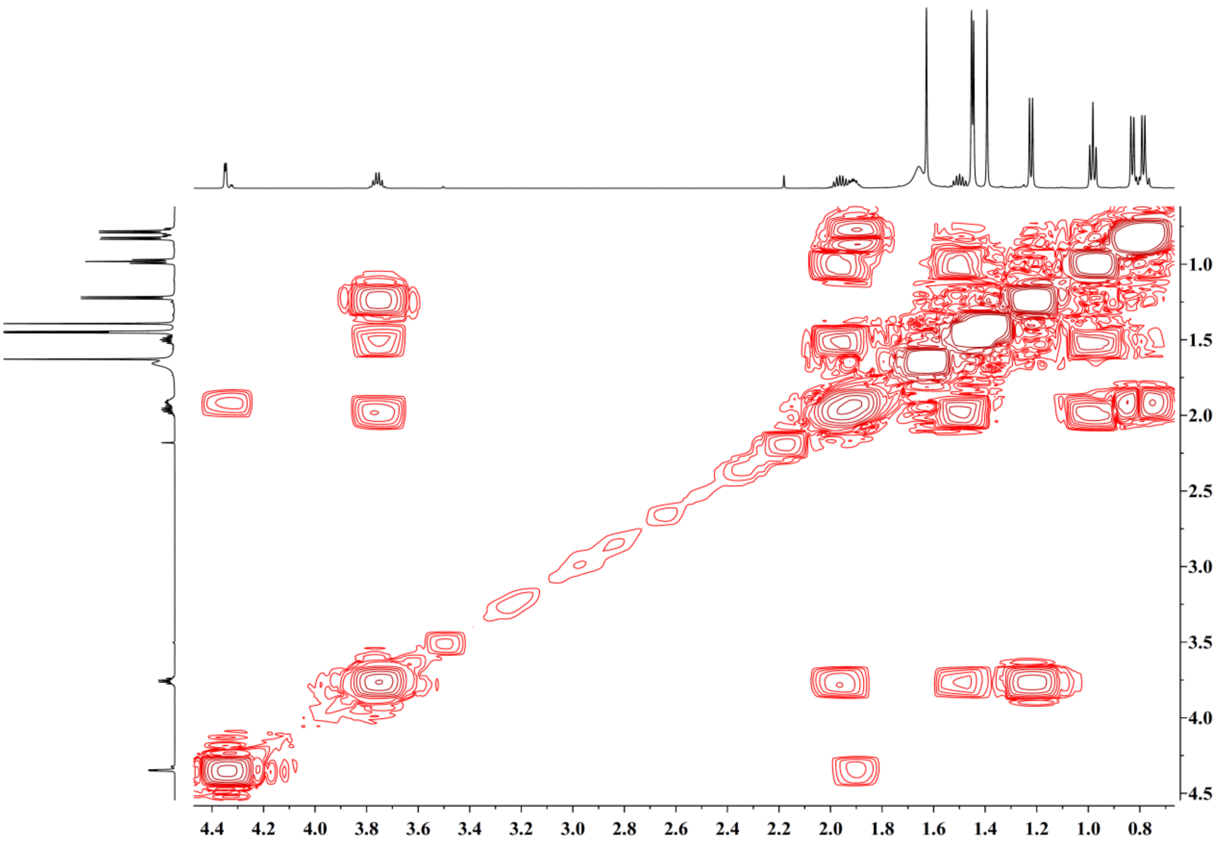

Figure S23 $^{1}H–^{1}H$ COSY spectrum of **4**

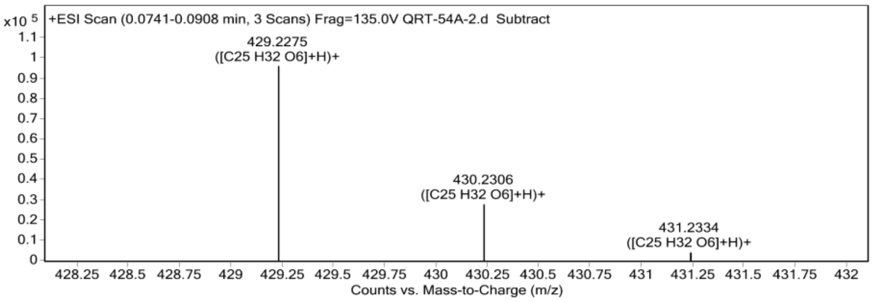

Peak List

m/z	z	Abund	Formula	Ion
274.2742	1	14689.51		
429.2275	1	96297.59	C25 H32 O6	(M+H)+
430.2306	1	28150.73	C25 H32 O6	(M+H)+
431.2334	1	4449.43	C25 H32 O6	(M+H)+
663.4531	1	6362.3		
680.4805	1	11399.35		
681.4847	1	4703		
764.5747	1	8454.27		
765.5783	1	4342.27		
879.4296	1	4681.16		

Formula Calculator Element Limits

Element	Min	Max
C	3	60
H	0	100
O	0	20

Formula Calculator Results

Formula	CalculatedMass	CalculatedMz	Mz	Diff. (mDa)	Diff. (ppm)	DBE
C25 H32 O6	428.2199	429.2272	429.2275	-0.30	-0.70	10.0000

Figure S24. HRESIMS spectrum of **4**

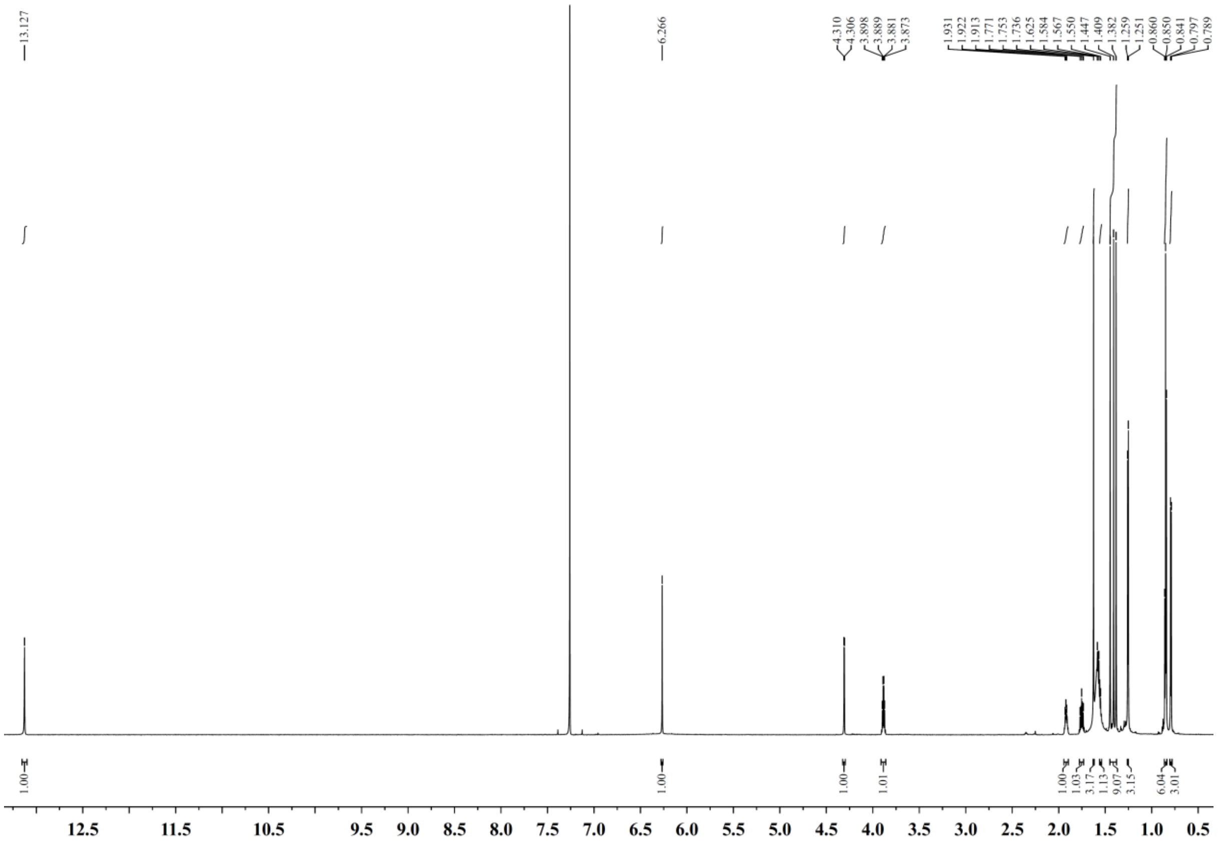

Figure S25. ^{1}H NMR spectrum of **5**

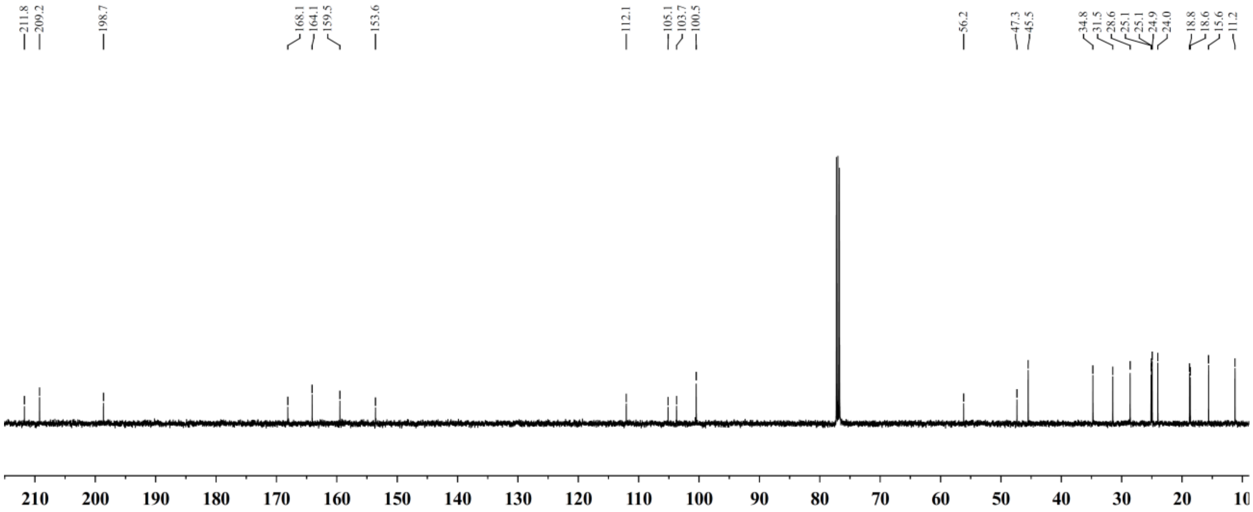

Figure S26. ^{13}C NMR spectrum of **5**

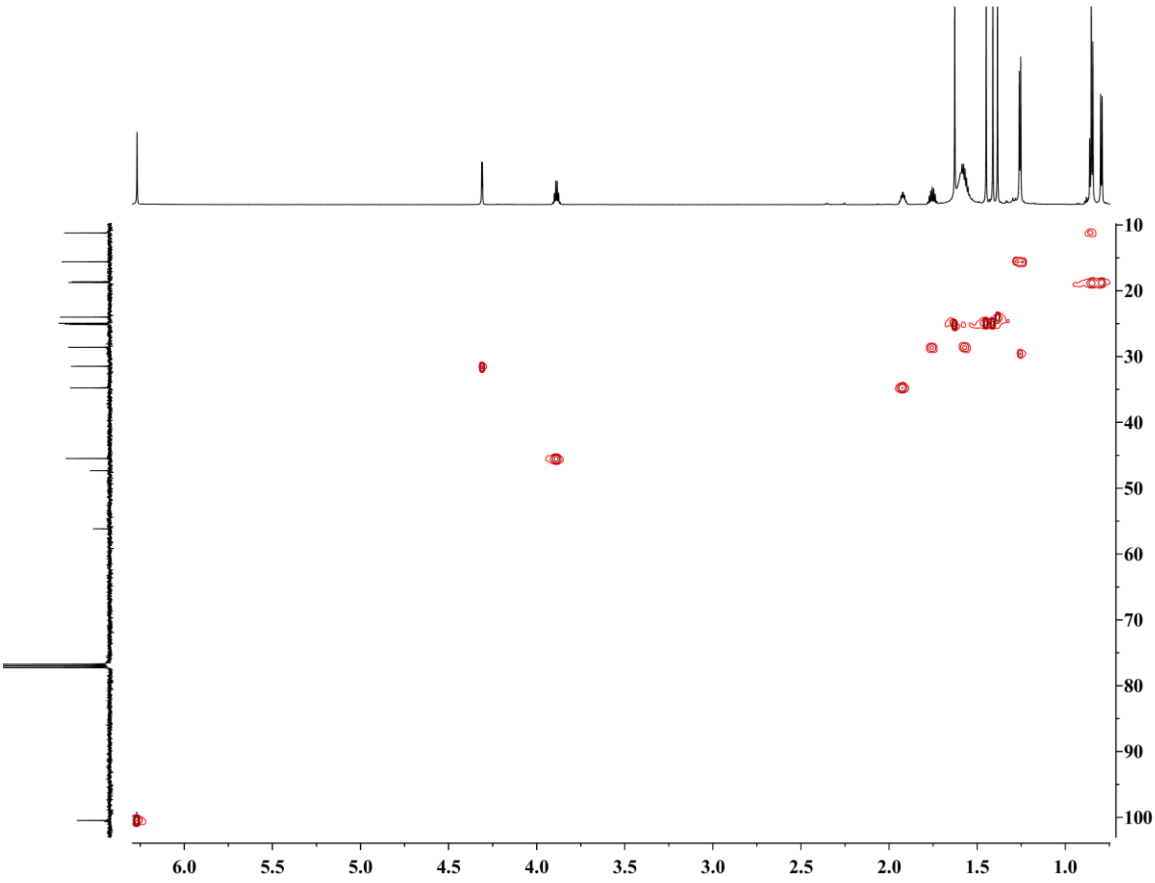

Figure S27. HSQC spectrum of **5**

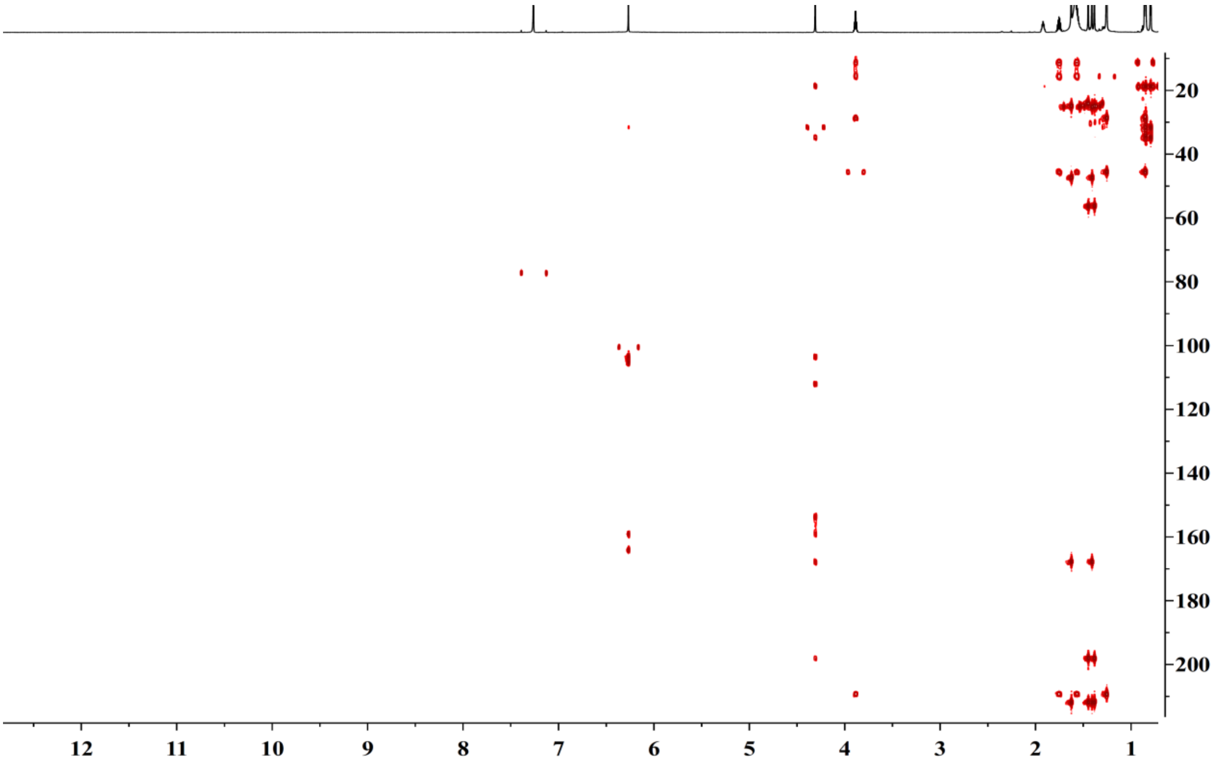

Figure S28. HMBC spectrum of **5**

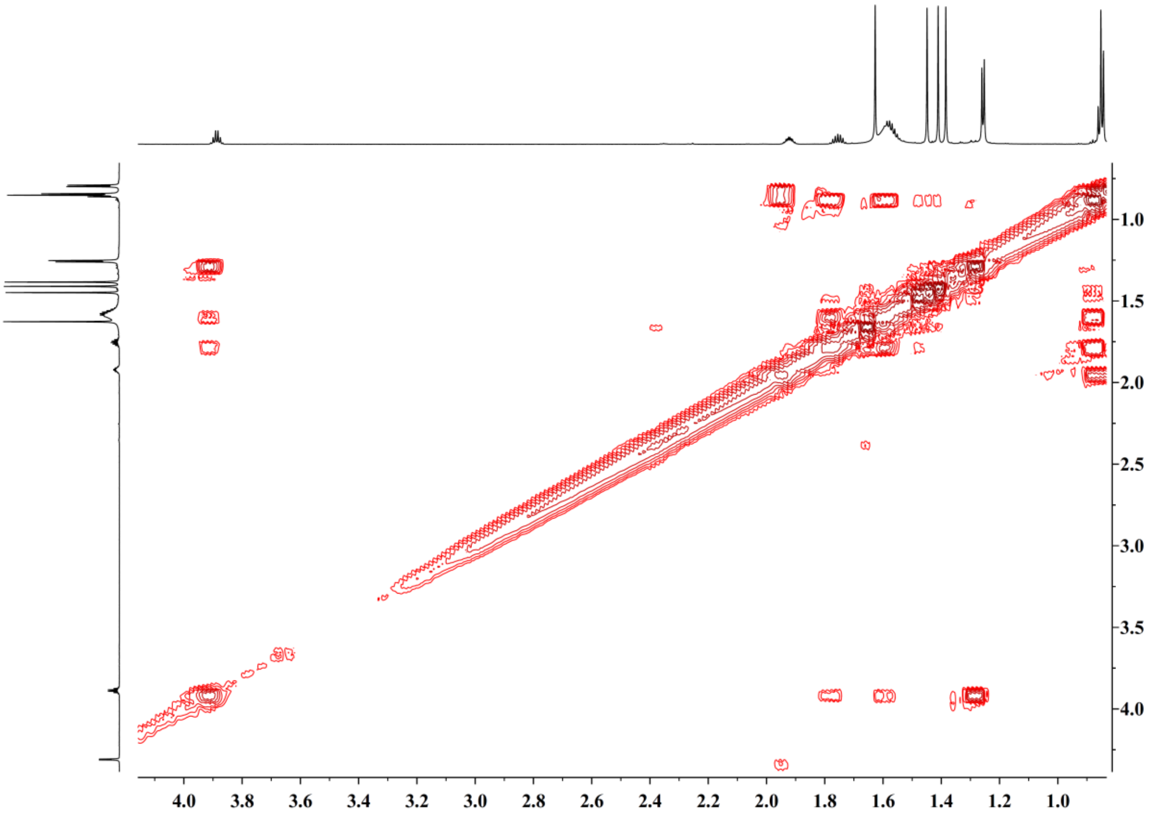

Figure S29. ^{1}H–^{1}H COSY spectrum of **5**

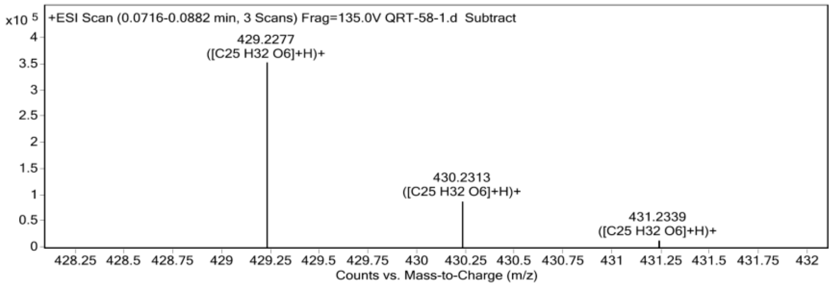

Peak List

m/z	z	Abund	Formula	Ion
429.2277	1	353997.84	C25 H32 O6	(M+H)+
430.2313	1	89932.82	C25 H32 O6	(M+H)+
431.2339	1	14577.02	C25 H32 O6	(M+H)+
443.2428	1	11954.44		
857.4488	1	76322.45		
858.4525	1	41885.35		
859.4551	1	12377.44		
879.4314	1	101477.54		
880.4341	1	55522.69		
881.4364	1	15727.57		

Formula Calculator Element Limits

Element	Min	Max
C	3	60
H	0	100
O	0	20

Formula Calculator Results

Formula	CalculatedMass	CalculatedMz	Mz	Diff. (mDa)	Diff. (ppm)	DBE
C25 H32 O6	428.2199	429.2272	429.2277	-0.50	-1.16	10.0000

Figure S30. HRESIMS spectrum of **5**

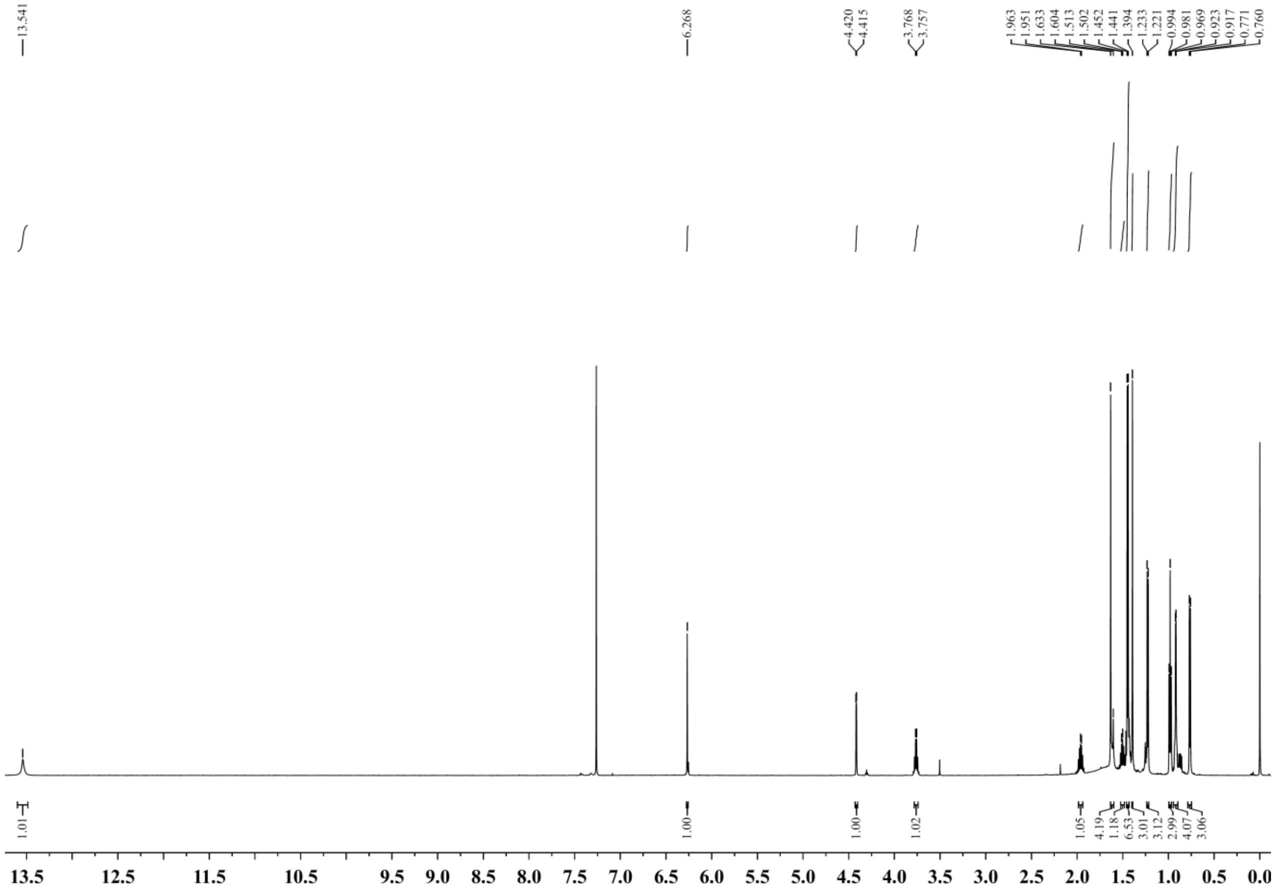

Figure S31. ^{1}H NMR spectrum of **6**

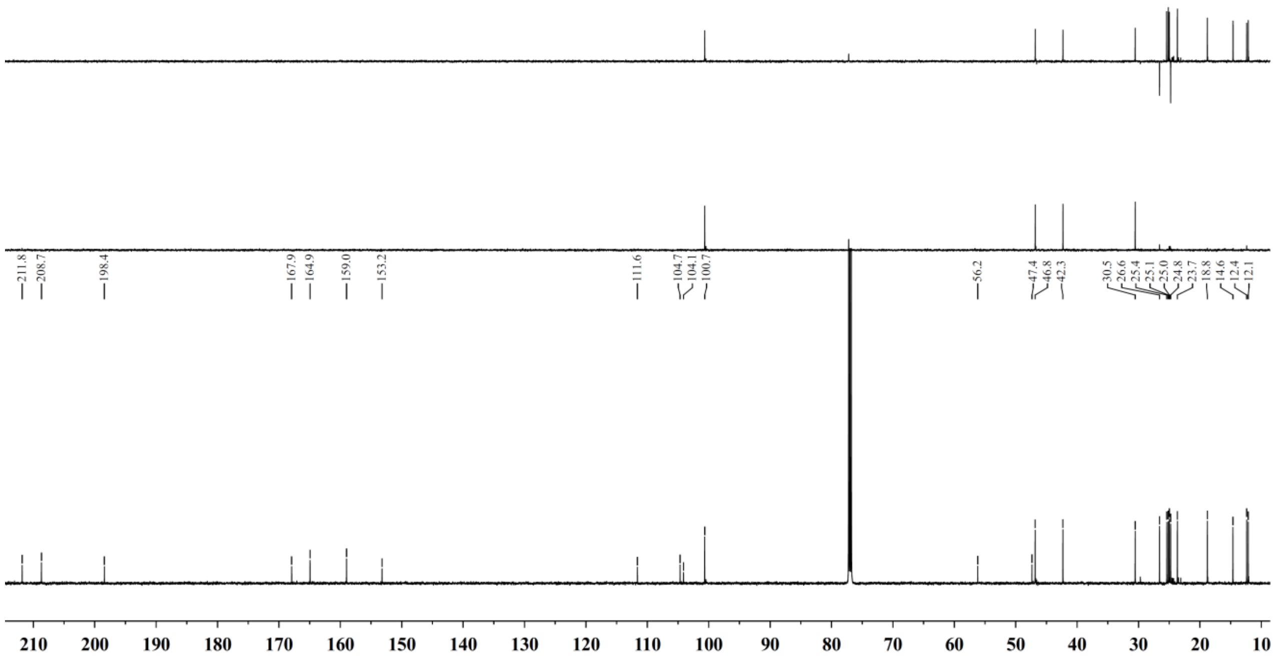

Figure S32. ^{13}C NMR spectrum of **6**

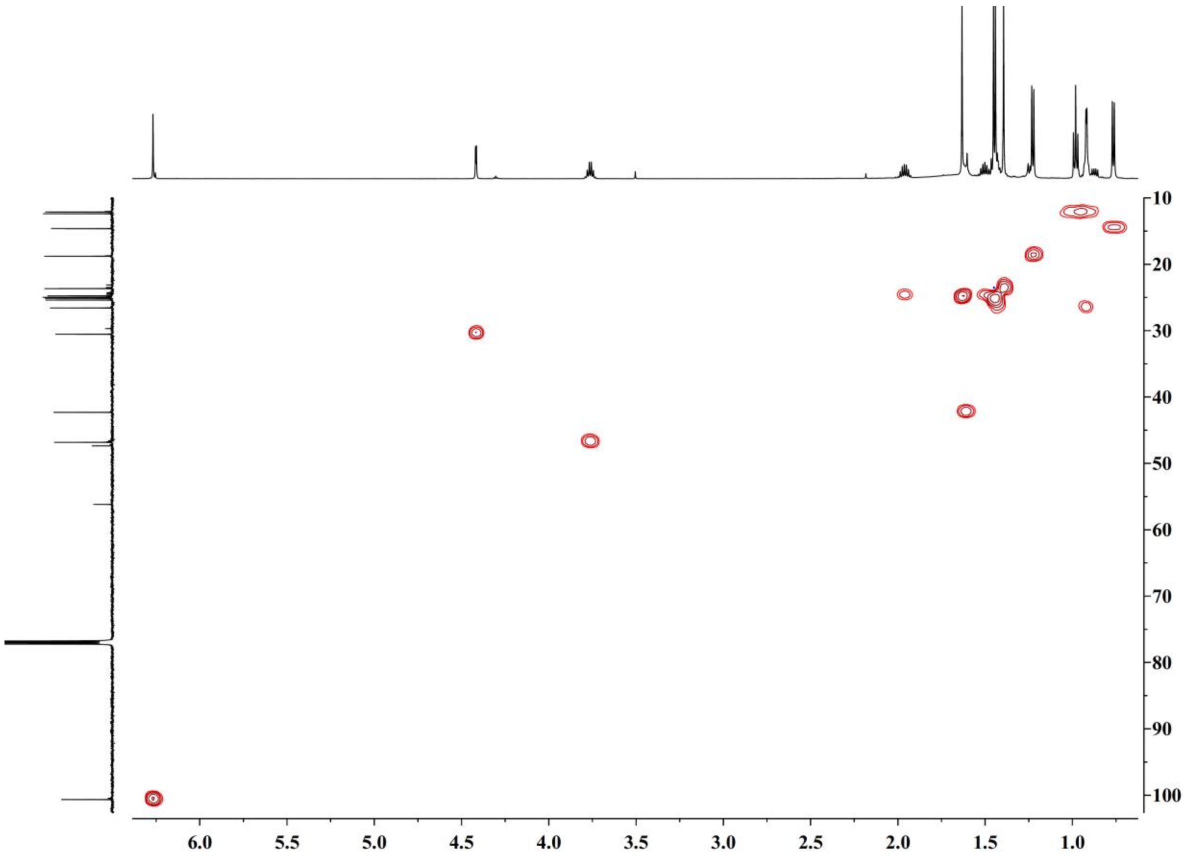

Figure S33. HSQC spectrum of **6**

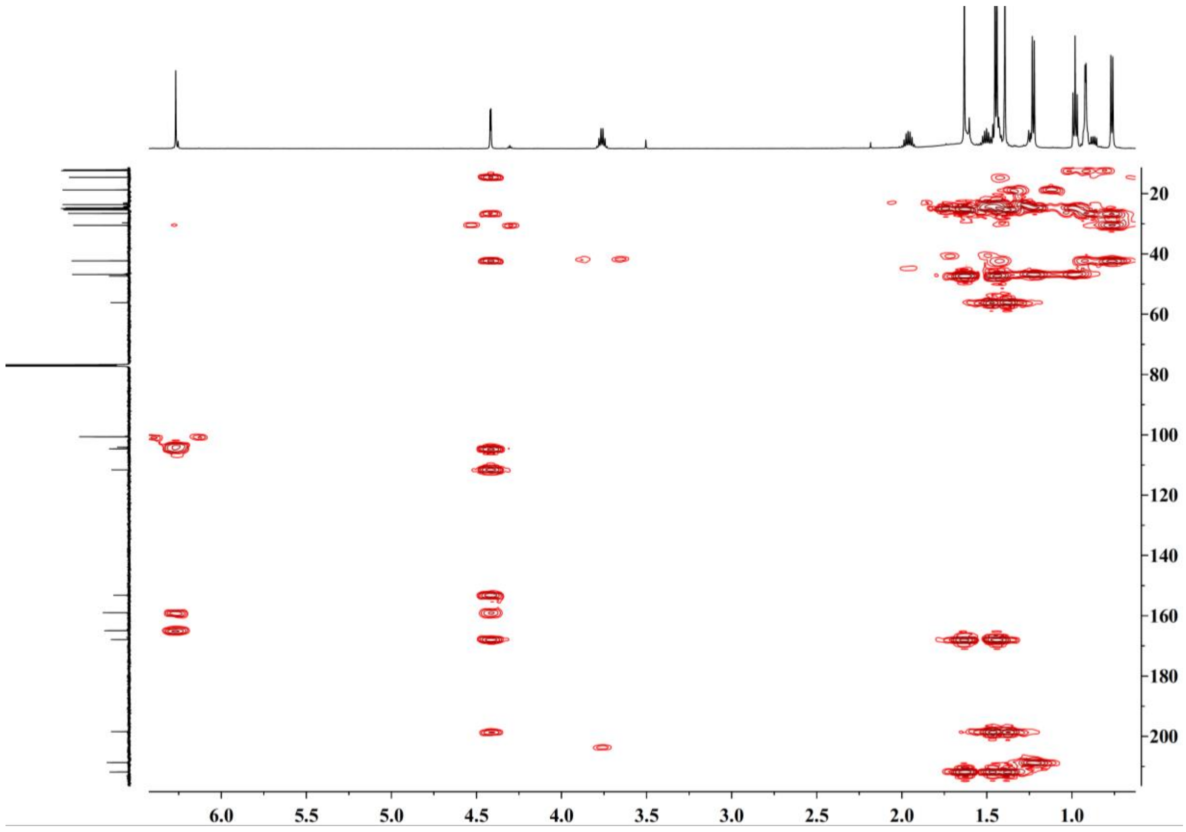

Figure S34. HMBC spectrum of **6**

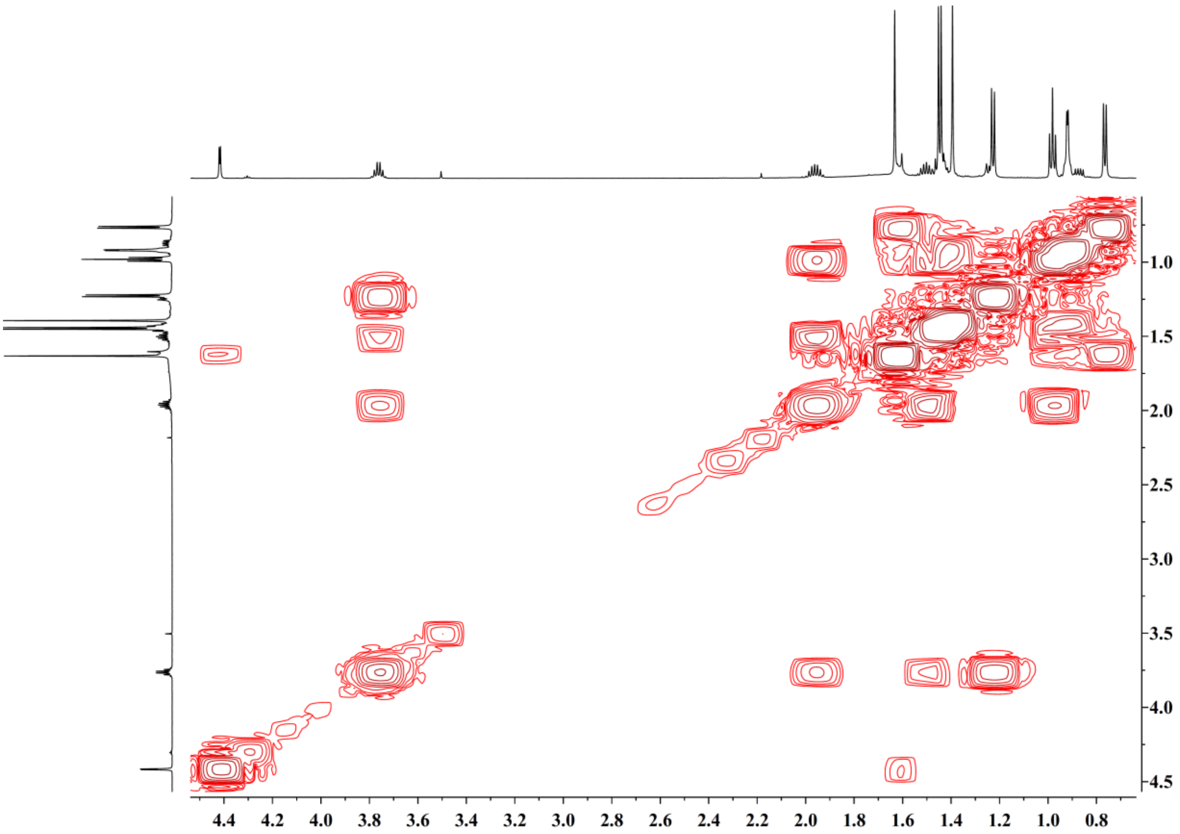

Figure S35. ^{1}H–^{1}H COSY spectrum of **6**

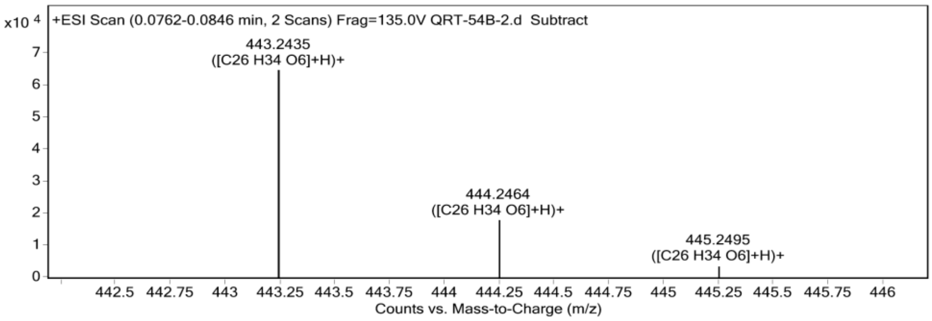

Peak List

m/z	z	Abund	Formula	Ion
102.128	1	2614.57		
274.2748	1	4971.24		
429.2271	1	6632.3		
443.2435	1	64868.54	C26 H34 O6	(M+H)+
444.2464	1	18021.44	C26 H34 O6	(M+H)+
445.2495	1	3768.46	C26 H34 O6	(M+H)+
510.2491	1	7478.01		
511.2514	1	2360.72		
579.2139	1	2275.6		
907.4619	1	2339.49		

Formula Calculator Element Limits

Element	Min	Max
C	3	60
H	0	100
O	0	20

Formula Calculator Results

Formula	CalculatedMass	CalculatedMz	Mz	Diff. (mDa)	Diff. (ppm)	DBE
C26 H34 O6	442.2355	443.2428	443.2435	-0.70	-1.58	10.0000

Figure S36. HRESIMS spectrum of **6**

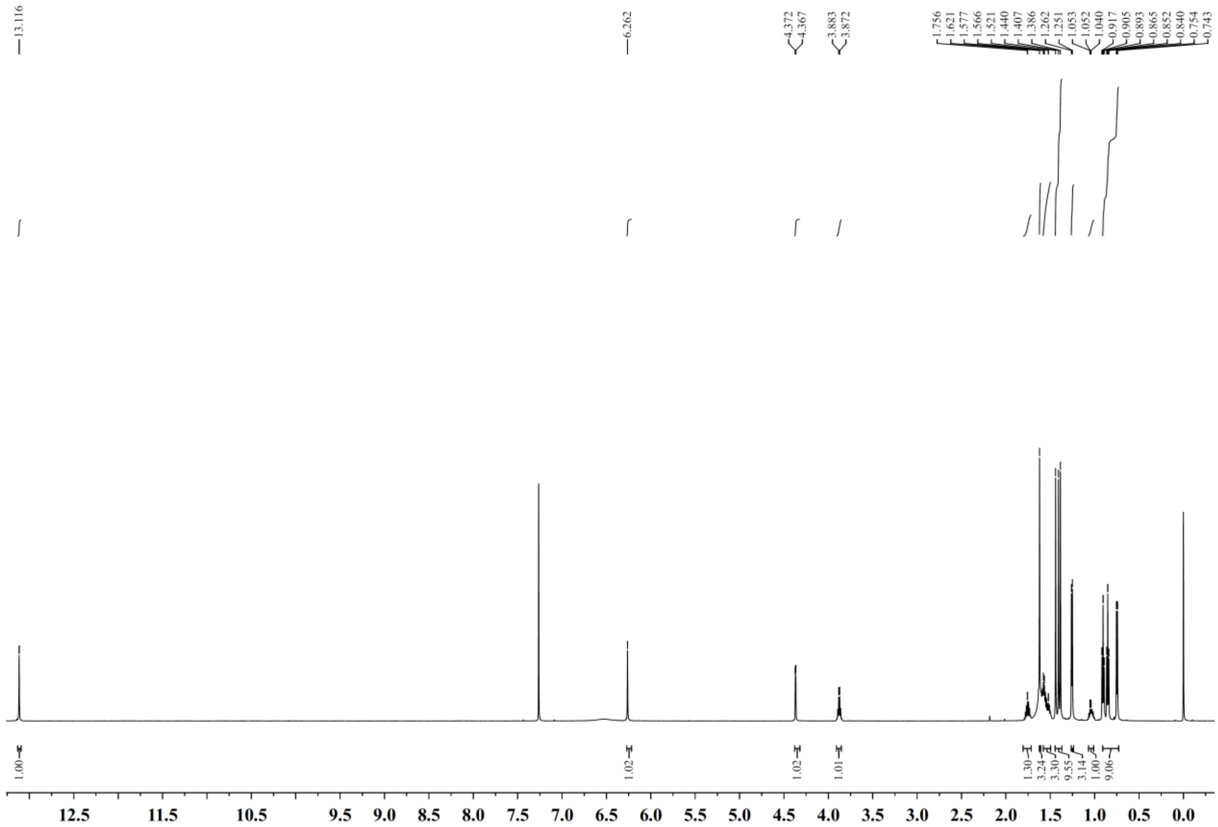

Figure S37. ^{1}H NMR spectrum of **7**

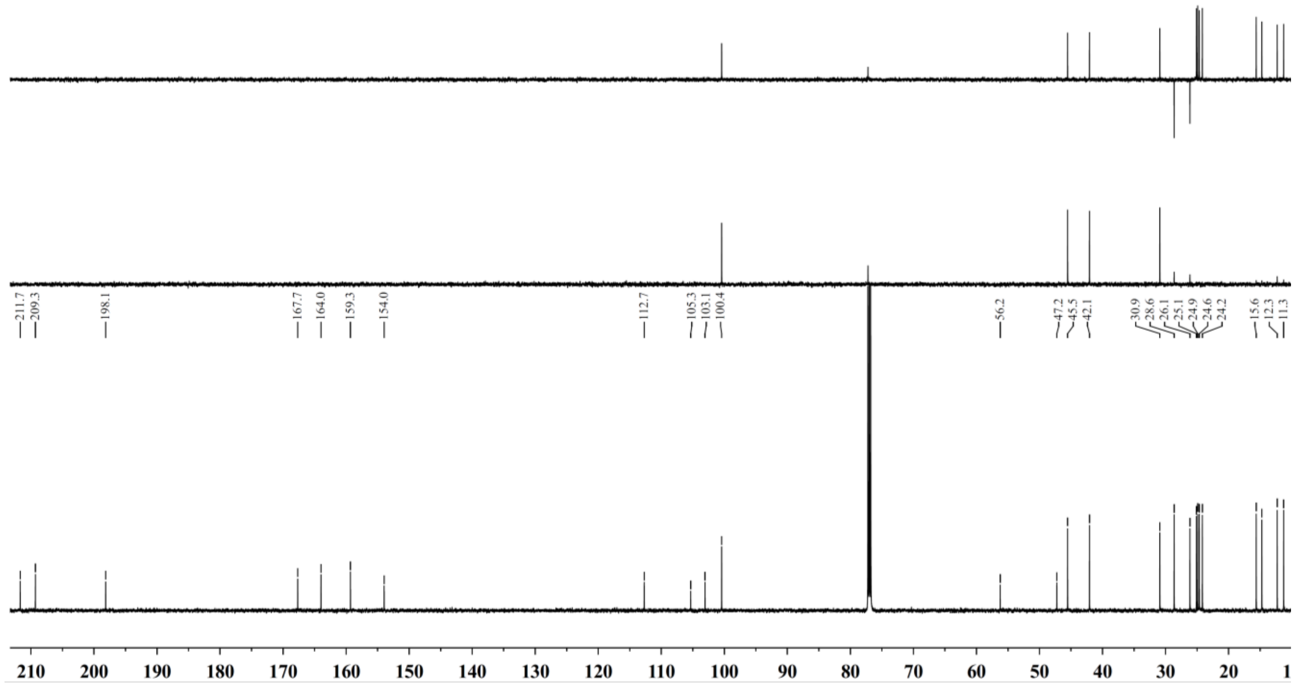

Figure S38. ^{13}C NMR spectrum of **7**

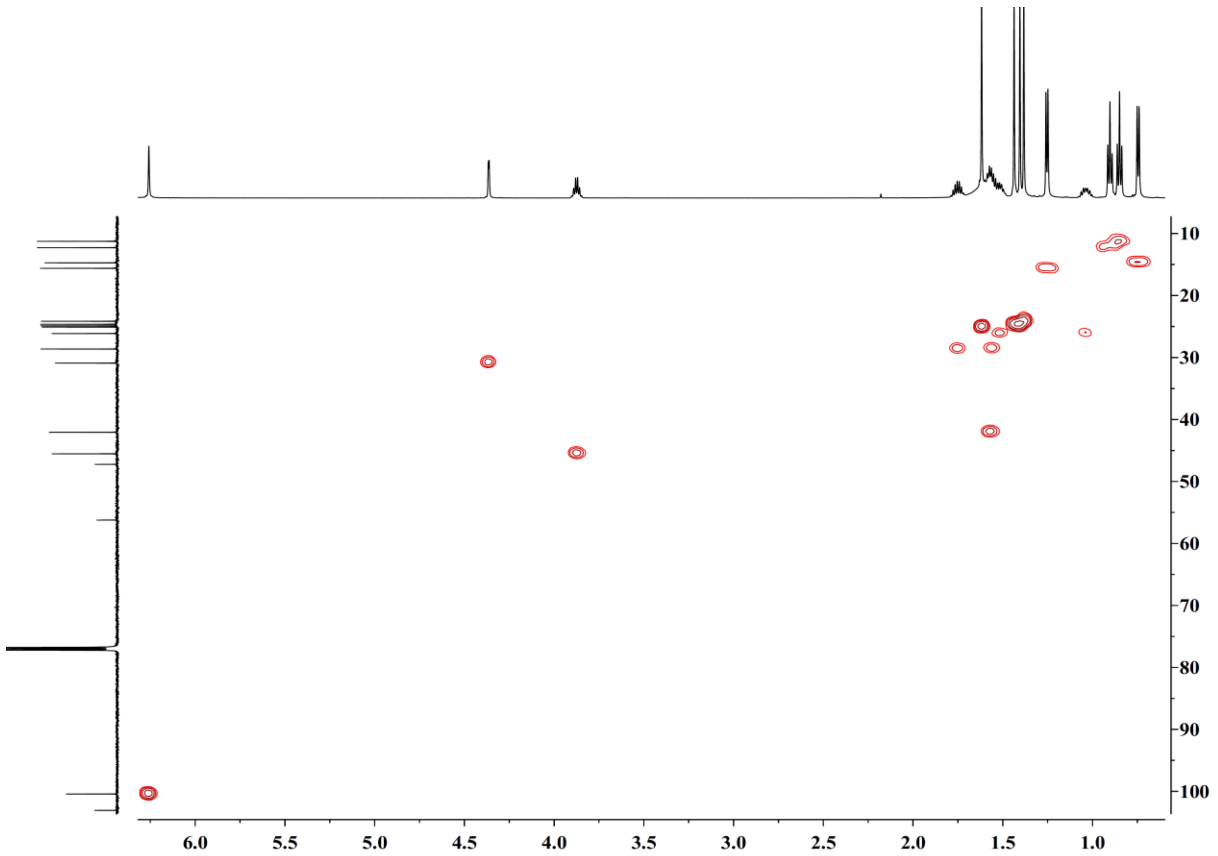

Figure S39. HSQC spectrum of **7**

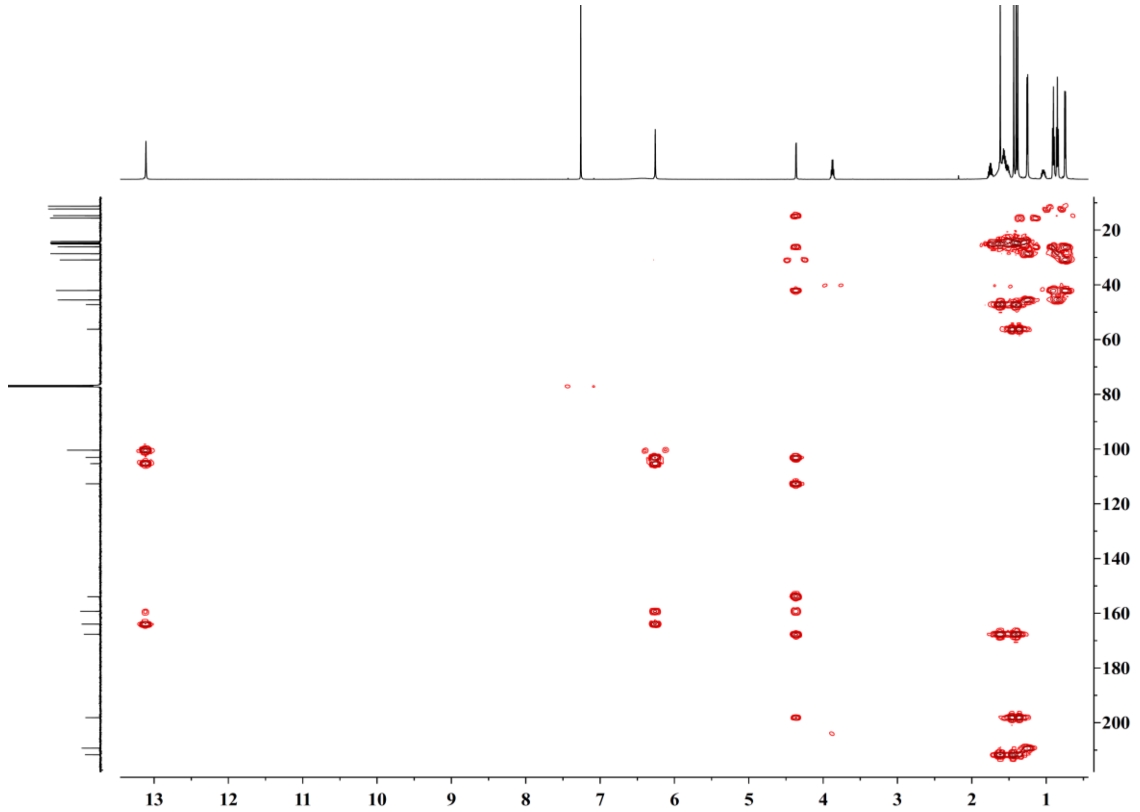

Figure S40. HMBC spectrum of **7**

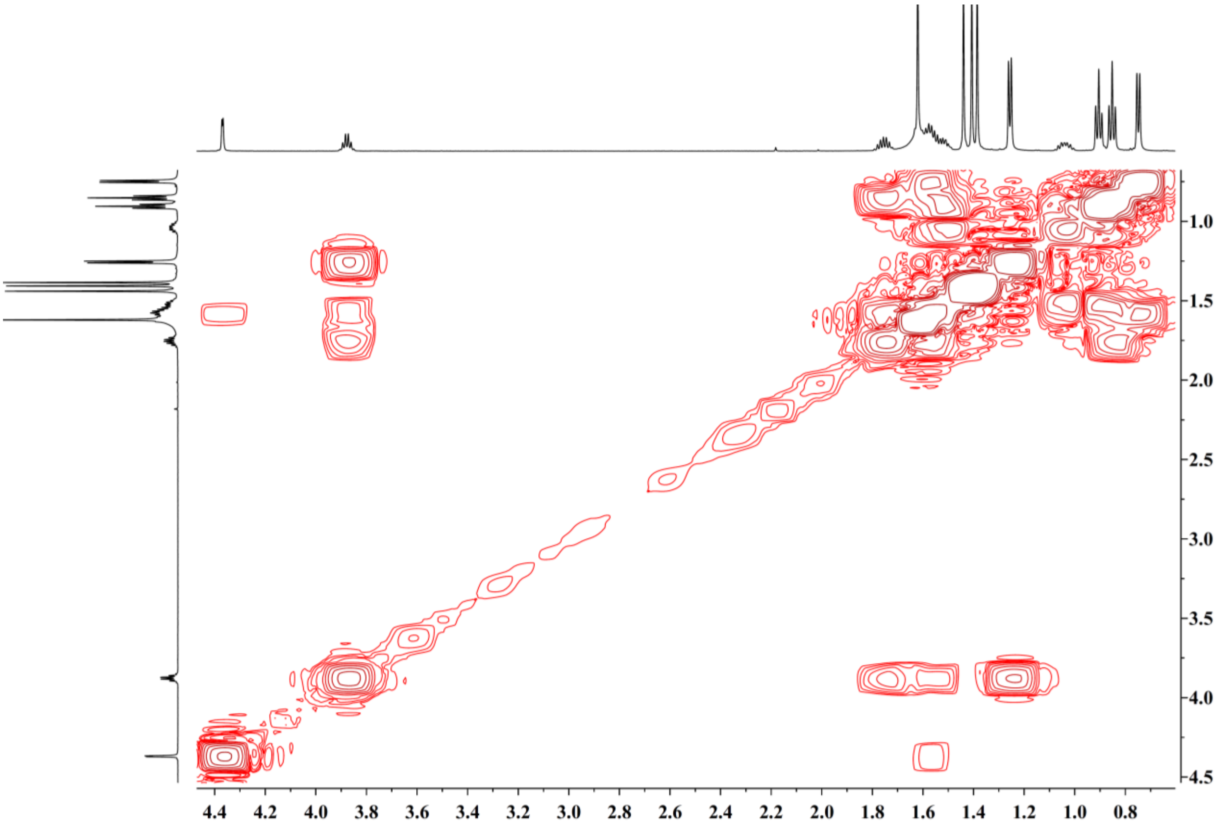

Figure S41. ^{1}H–^{1}H COSY spectrum of **7**

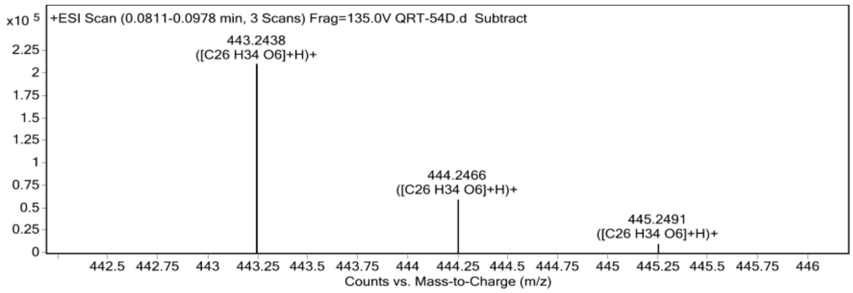

Peak List

m/z	z	Abund	Formula	Ion
274.2746	1	8106.99		
429.2278	1	10410.12		
443.2438	1	210787.17	C26 H34 O6	(M+H)+
444.2466	1	60485.69	C26 H34 O6	(M+H)+
445.2491	1	11365.38	C26 H34 O6	(M+H)+
510.2488	1	12617.42		
511.2525	1	4203.73		
579.2154	1	5704.34		
907.4615	1	16983.85		
908.4657	1	8562.82		

Formula Calculator Element Limits

Element	Min	Max
C	3	60
H	0	100
O	0	20

Formula Calculator Results

Formula	CalculatedMass	CalculatedMz	Mz	Diff. (mDa)	Diff. (ppm)	DBE
C26 H34 O6	442.2355	443.2428	443.2438	-1.00	-2.26	10.0000

Figure S42. HRESIMS spectrum of **7**

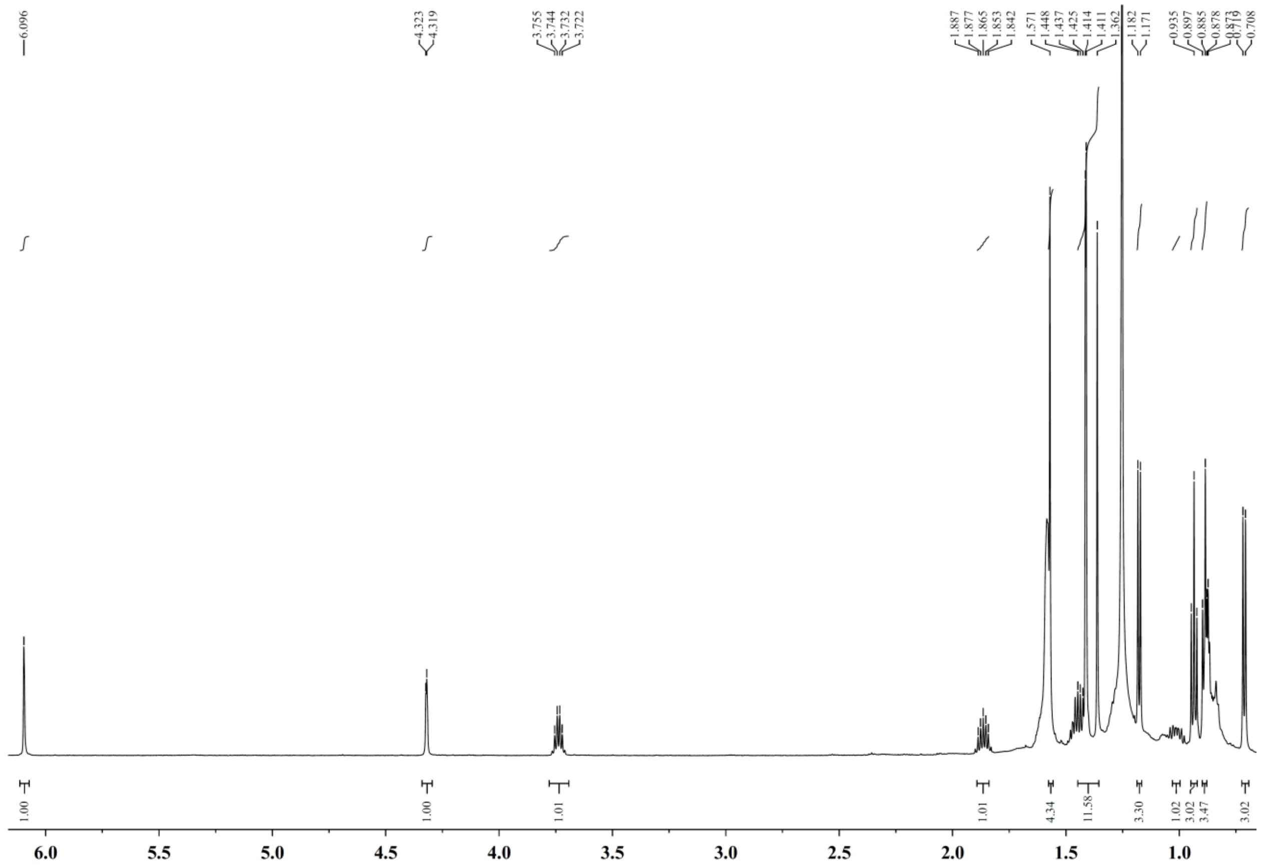

Figure S43. ^{1}H NMR spectrum of **8**

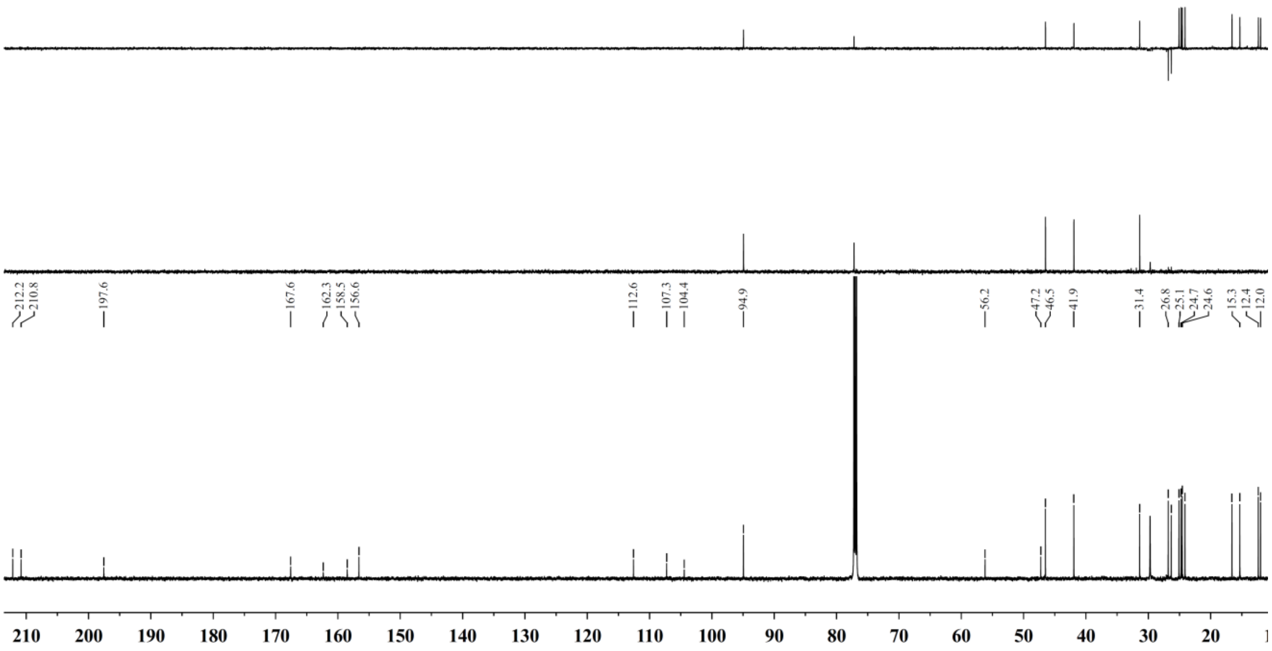

Figure S44. ^{13}C NMR spectrum of **8**

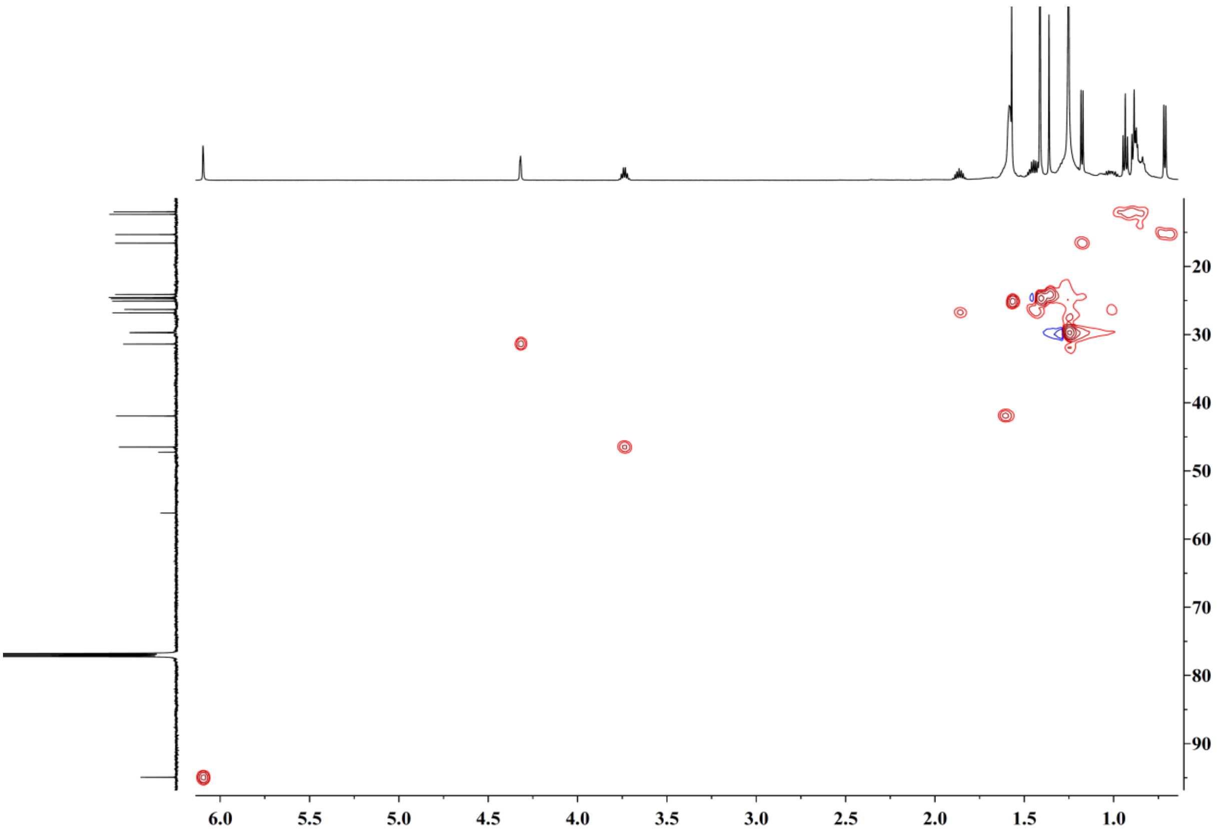

Figure S45. HSQC spectrum of **8**

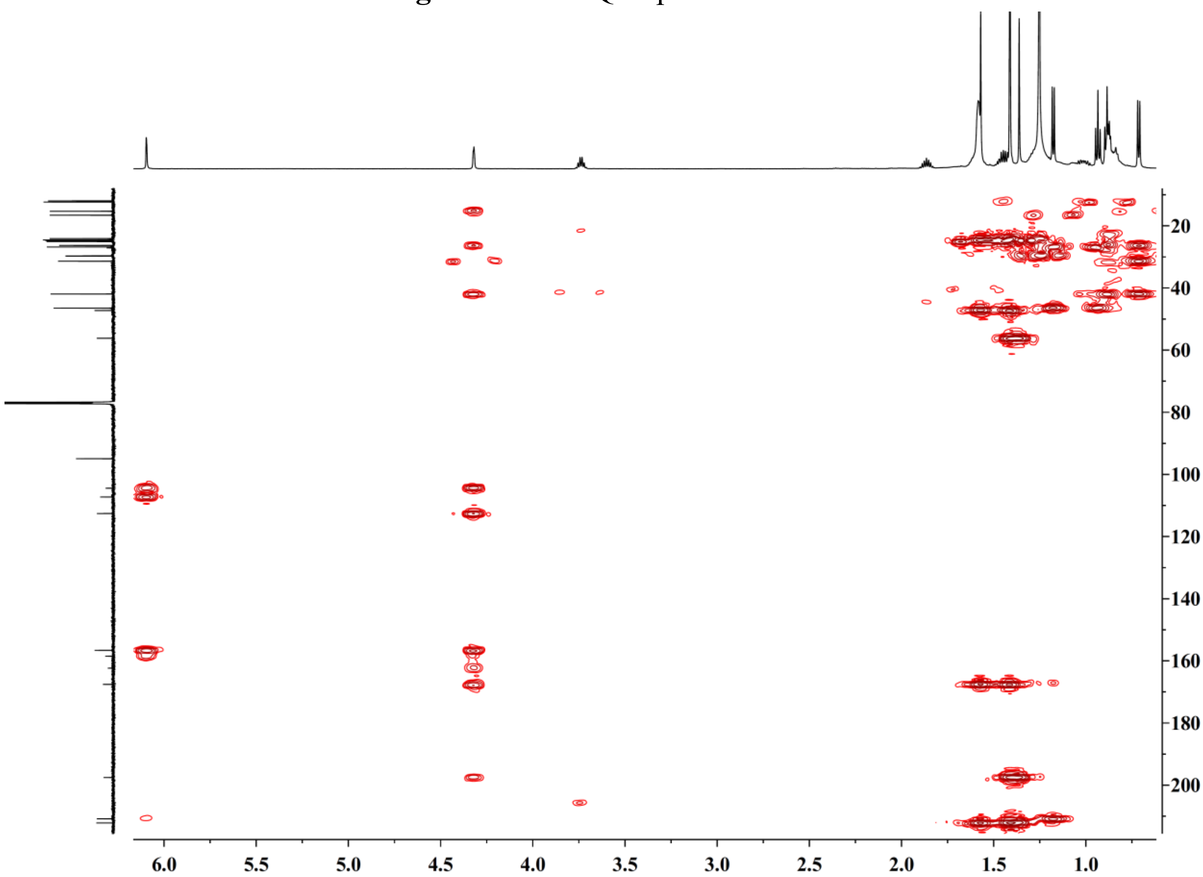

Figure S46. HMBC spectrum of **8**

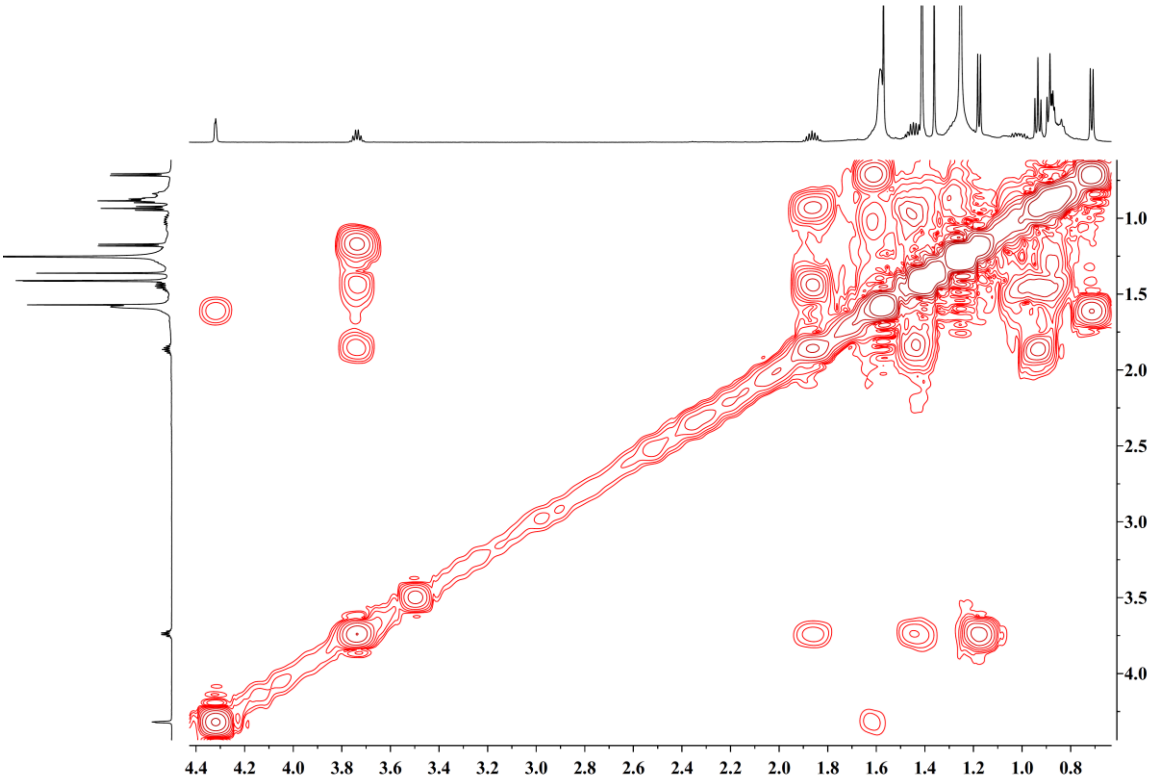

Figure S47. ^{1}H–^{1}H COSY spectrum of **8**

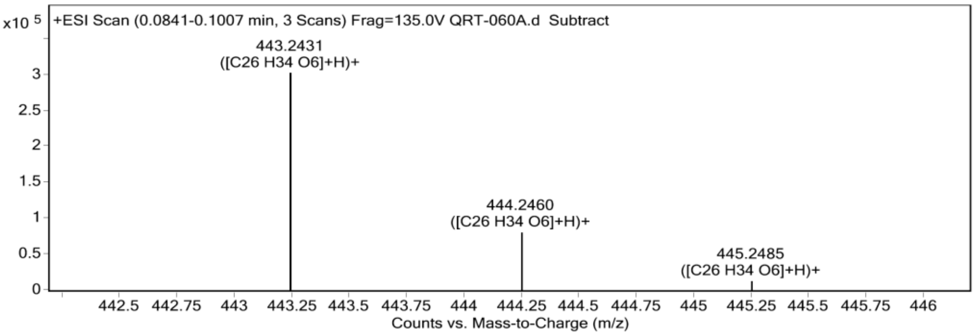

Peak List

m/z	z	Abund	Formula	Ion
443.2431	1	302851	C26 H34 O6	(M+H)+
444.246	1	81322.47	C26 H34 O6	(M+H)+
445.2485	1	13512.84	C26 H34 O6	(M+H)+
579.2146	1	12430.84		
883.4618	1	15276.2		
885.477	1	70696.3		
886.4803	1	38380.84		
887.4834	1	12835.74		
907.4592	1	57294.37		
908.4622	1	31922.51		

Formula Calculator Element Limits

Element	Min	Max
C	3	60
H	0	100
O	0	20

Formula Calculator Results

Formula	CalculatedMass	CalculatedMz	Mz	Diff. (mDa)	Diff. (ppm)	DBE
C26 H34 O6	442.2355	443.2428	443.2431	-0.30	-0.68	10.0000

Figure S48. HRESIMS spectrum of **8**

ECD calculated data for **1**

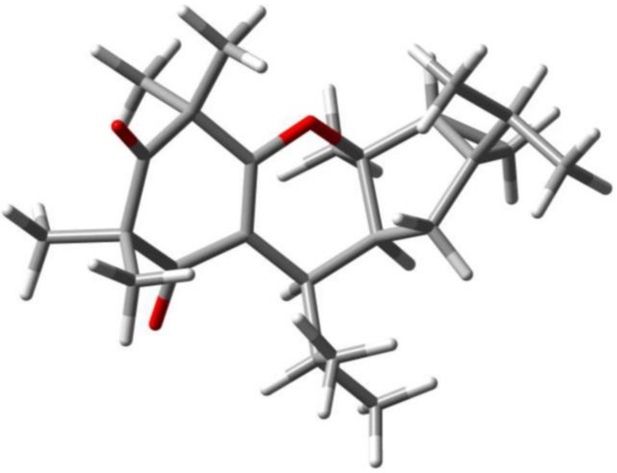

Conformer 1

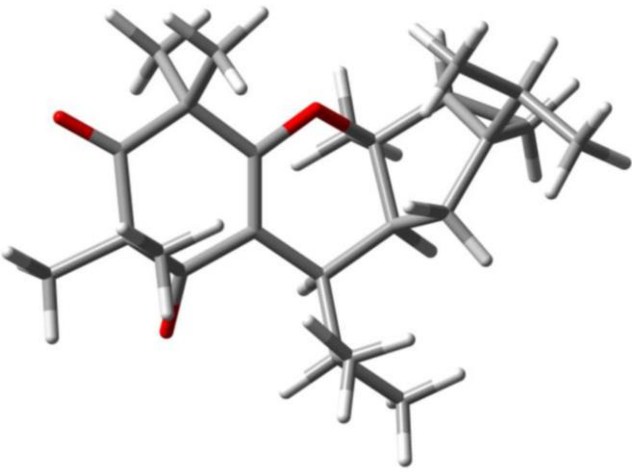

Conformer 2

Conformer No.	Distribution (%)	Relative Energy (kcal/mol)
1	65.4305	0.0000
2	31.1835	0.4391

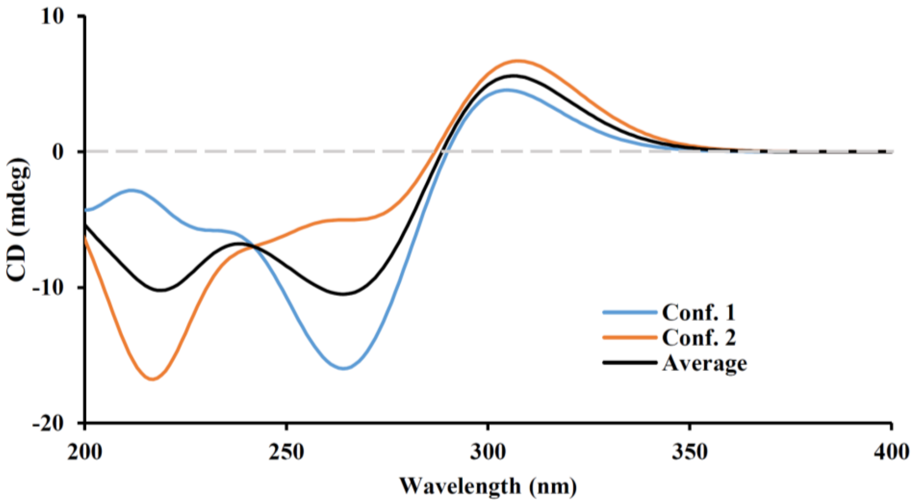

ECD calculated data for **2**

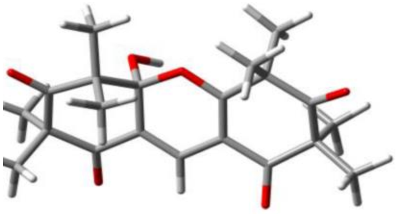

Conformer 1

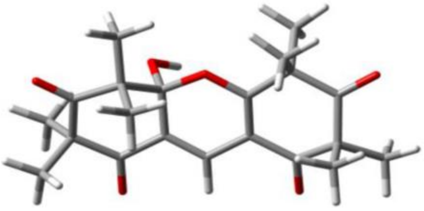

Conformer 2

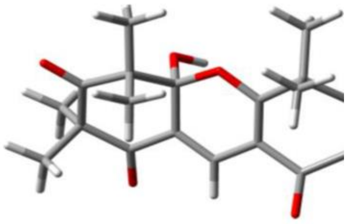

Conformer 3

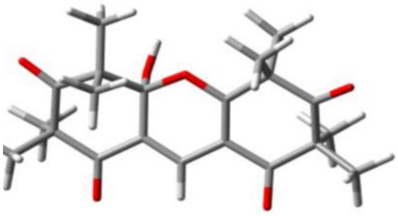

Conformer 4

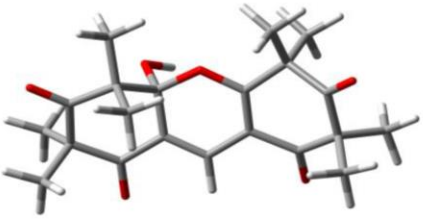

Conformer 5

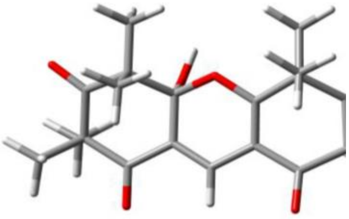

Conformer 6

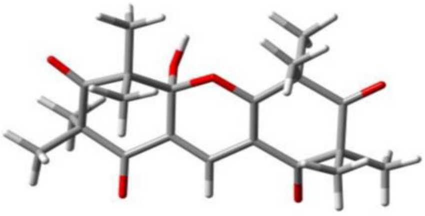

Conformer 7

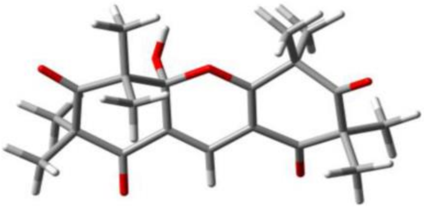

Conformer 8

Conformer No.	Distribution (%)	Relative Energy (kcal/mol)
1	19.2778	0.0000
2	17.5427	0.0559
3	14.7627	0.1581
4	13.2037	0.2242
5	11.4712	0.3076
6	10.8368	0.3413
7	8.0849	0.5148
8	4.8189	0.8214

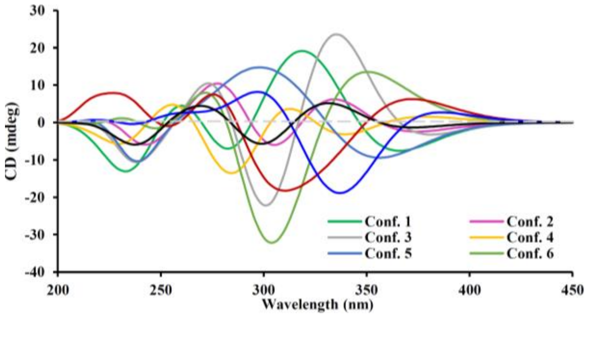

ECD calculated data for 7*S*,8*S*-**3**

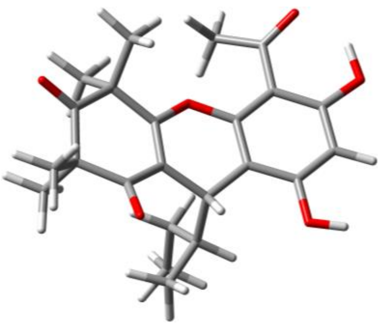

Conformer 1

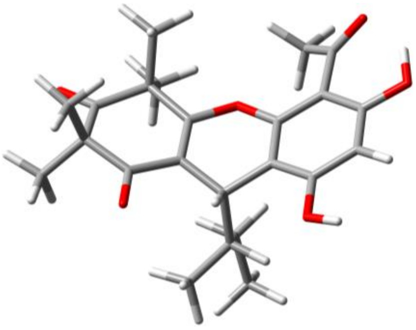

Conformer 2

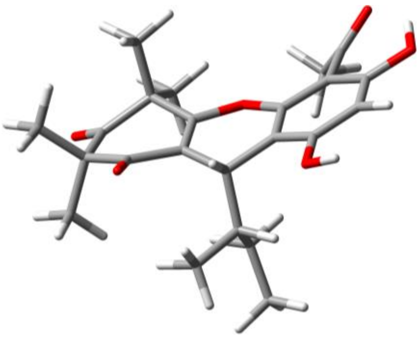

Conformer 3

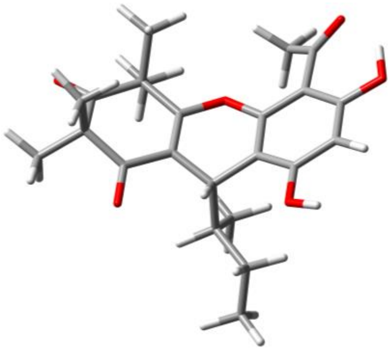

Conformer 4

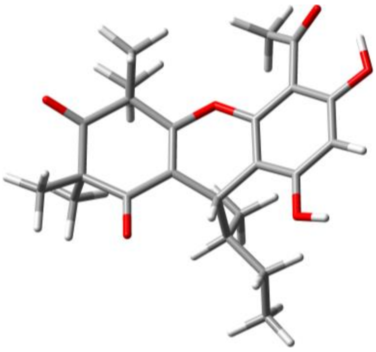

Conformer 5

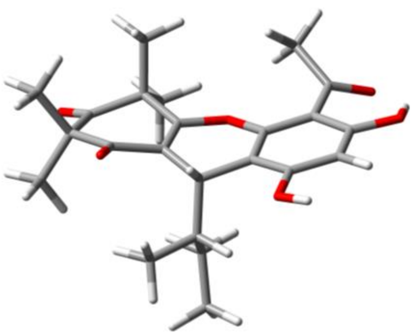

Conformer 6

Conformer No.	Distribution (%)	Relative Energy (kcal/mol)
1	33.4317	0.0000
2	13.9089	0.5196
3	13.4904	0.5377
4	9.2389	0.7620
5	7.6935	0.8704
6	6.5038	0.9700

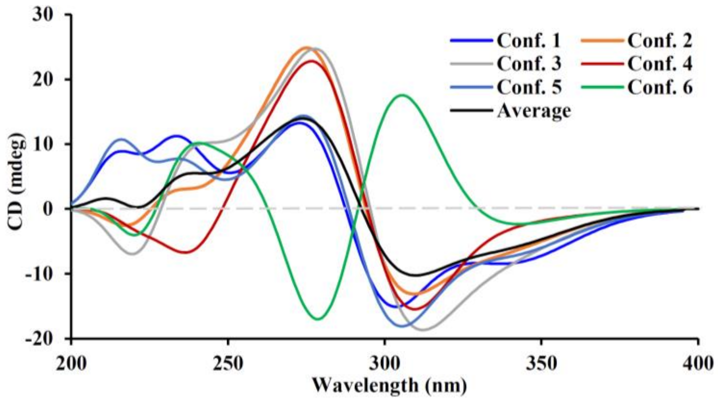

ECD calculated data for 7*R*,8′*S*-**4**

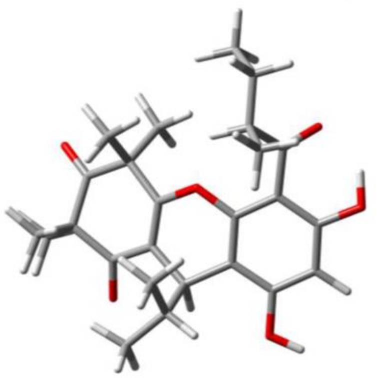

Conformer 1

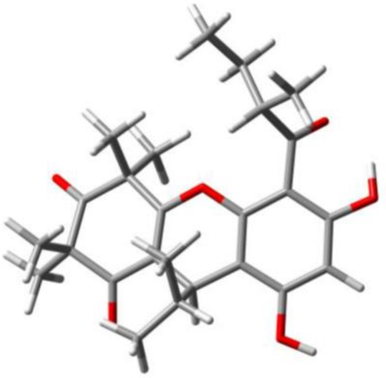

Conformer 2

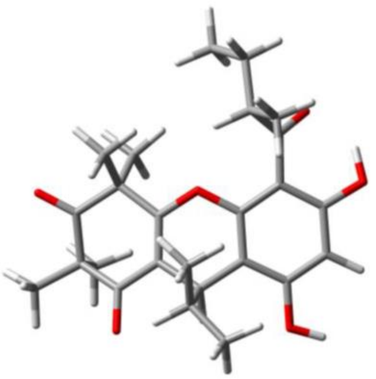

Conformer 3

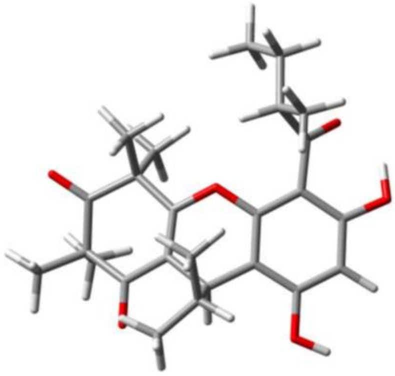

Conformer 4

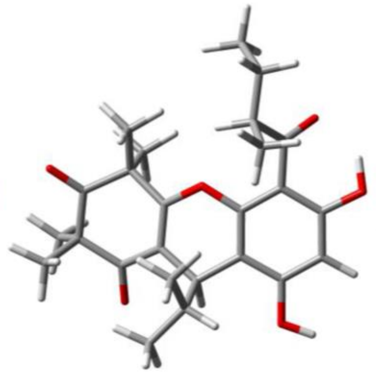

Conformer 5

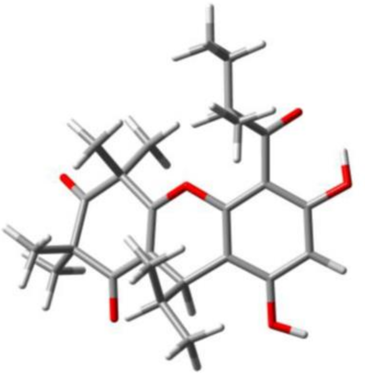

Conformer 6

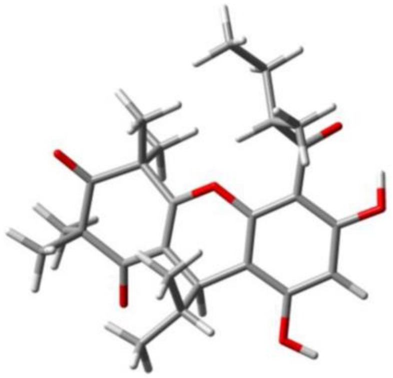

Conformer 7

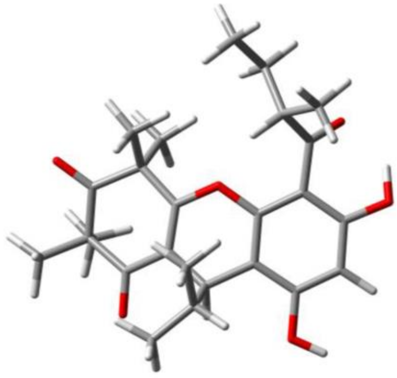

Conformer 8

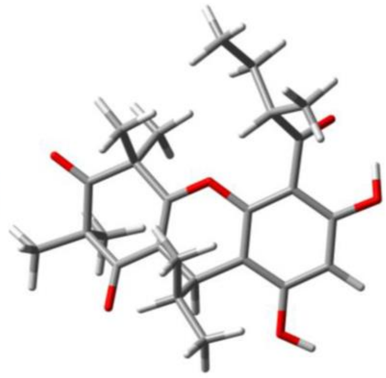

Conformer 9

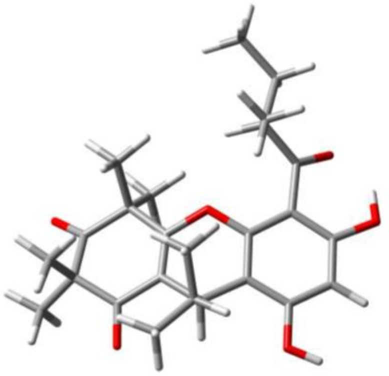

Conformer 10

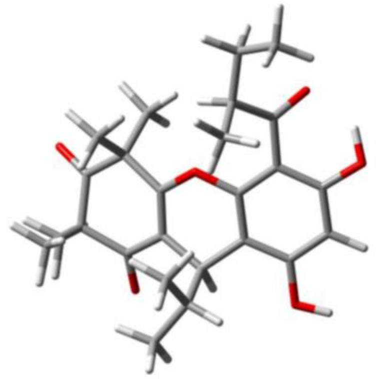

Conformer 11

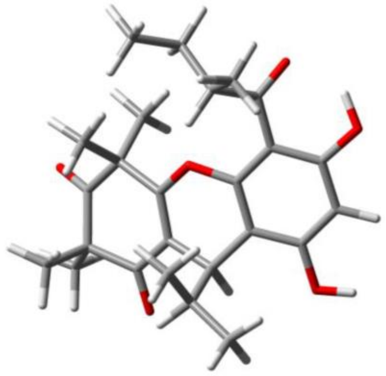

Conformer 12

Conformer No.	Distribution (%)	Relative Energy (kcal/mol)
1	16.5288	0.0000
2	8.3150	0.4071
3	7.9136	0.4364
4	7.5114	0.4673
5	7.2847	0.4854
6	6.5466	0.5487
7	7.8737	0.7236
8	4.0875	0.8278
9	4.0541	0.8327
10	3.5273	0.9151
11	3.4944	0.9207
12	3.3054	0.9536

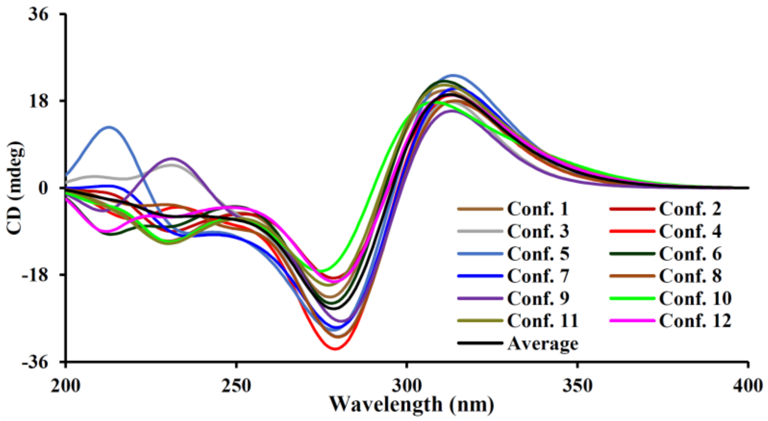

ECD calculated data for 7*R*,8*S*,8′*S*-**6**

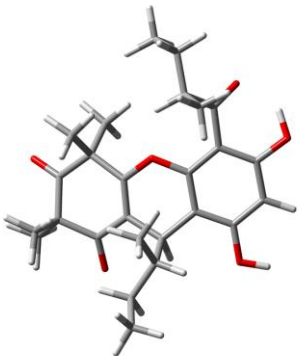
Conformer 1

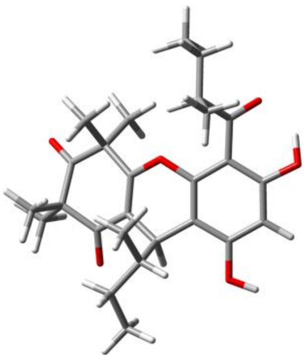
Conformer 2

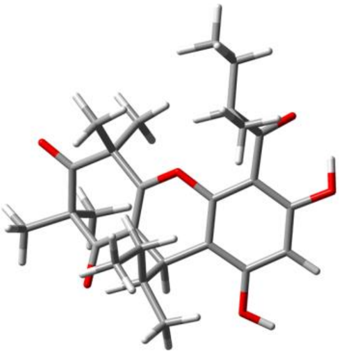
Conformer 3

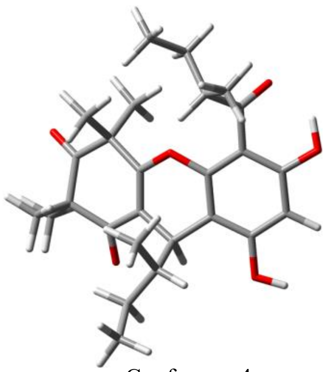
Conformer 4

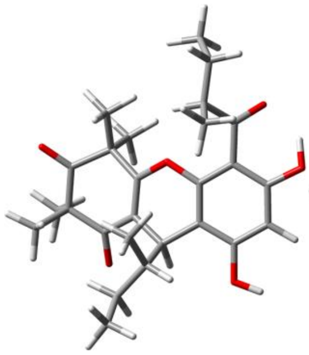
Conformer 5

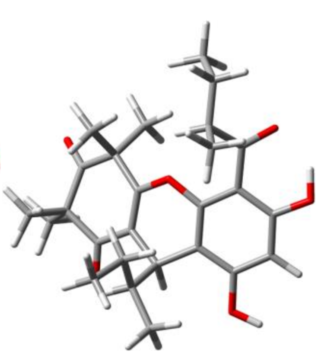
Conformer 6

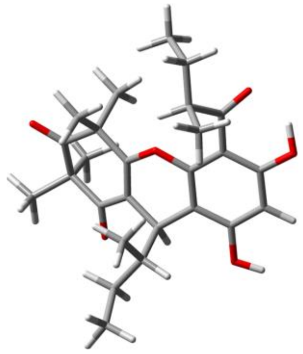
Conformer 7

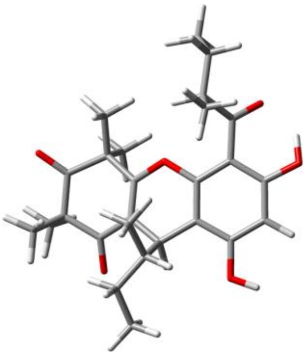
Conformer 8

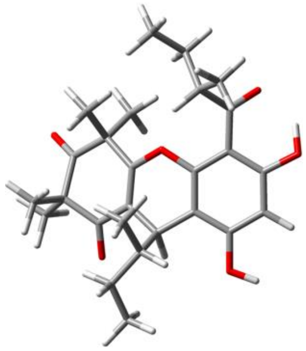
Conformer 9

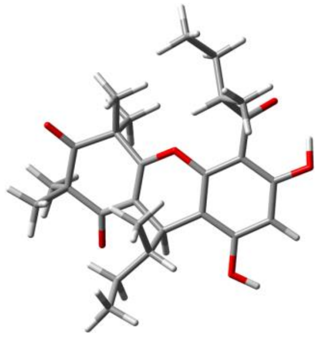
Conformer 10

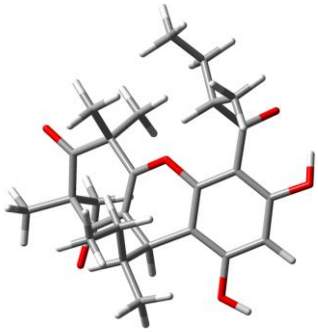
Conformer 11

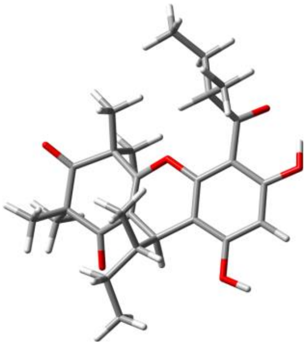
Conformer 12

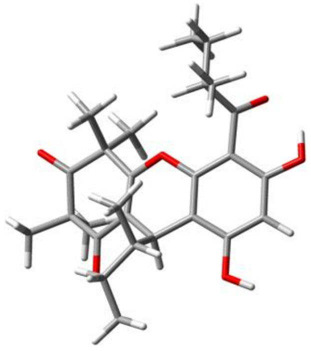
Conformer 13

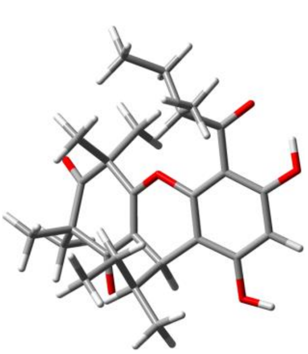
Conformer 14

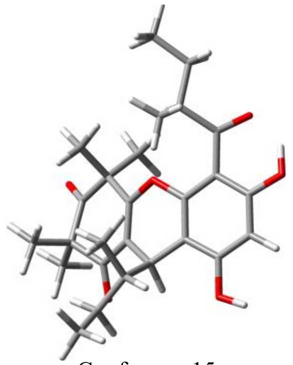
Conformer 15

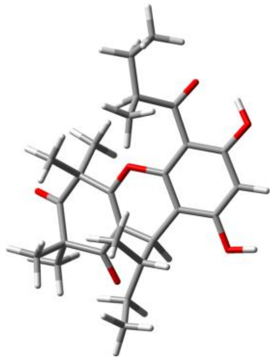
Conformer 16

Conformer No.	Distribution (%)	Relative Energy (kcal/mol)
1	11.5563	0.0000
2	7.6156	0.2471
3	6.0808	0.3804
4	5.8274	0.4078
5	5.700	0.4188
6	5.5229	0.4375
7	4.5920	0.5468
8	4.3374	0.5806
9	3.8532	0.6507
10	3.7964	0.6595
11	3.0728	0.7848
12	2.8581	0.8277
13	2.8104	0.8377
14	2.7643	0.8475
15	2.4543	0.9180
16	2.4050	0.9300

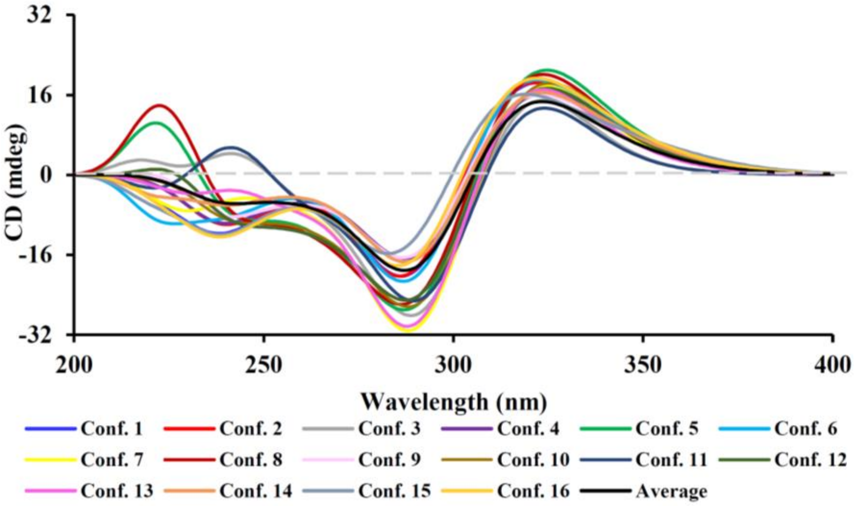

ECD calculated data for 7*S*,8*R*,8′*S*-**7**

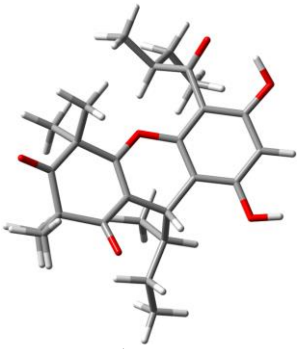

Conformer 1

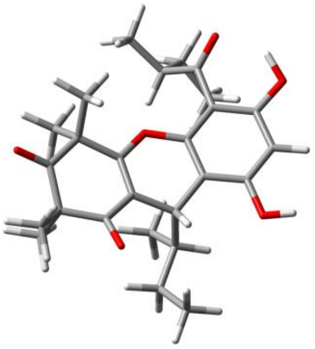

Conformer 2

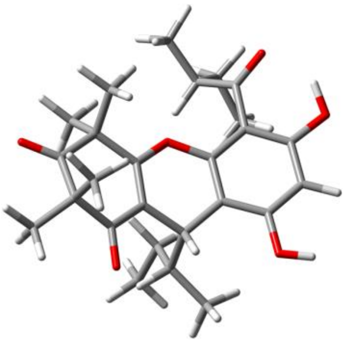

Conformer 3

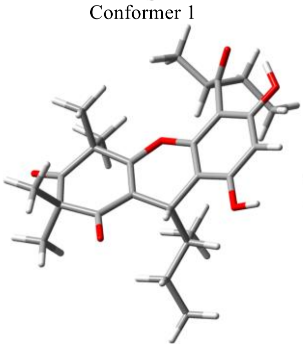

Conformer 4

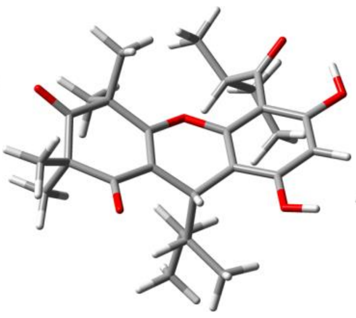

Conformer 5

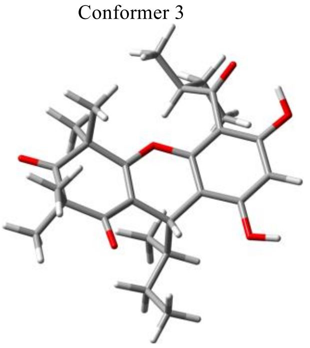

Conformer 6

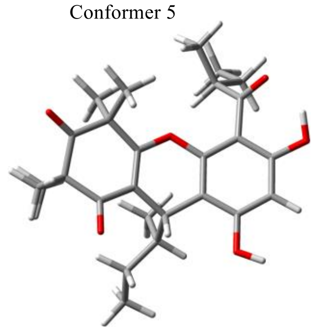

Conformer 7

Conformer No.	Distribution (%)	Relative Energy (kcal/mol)
1	18.2107	0.0000
2	12.0742	0.2435
3	10.1705	0.3451
4	9.1499	0.4078
5	9.0264	0.4158
6	5.6970	0.6885
7	3.3817	0.9975

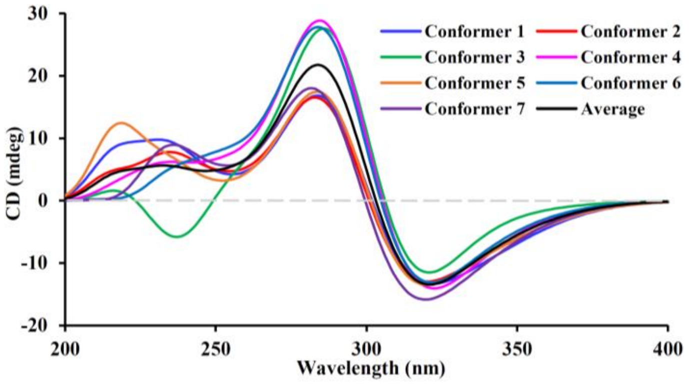

ECD calculated data for 7*S*,8*R*,8′*R*-**8**

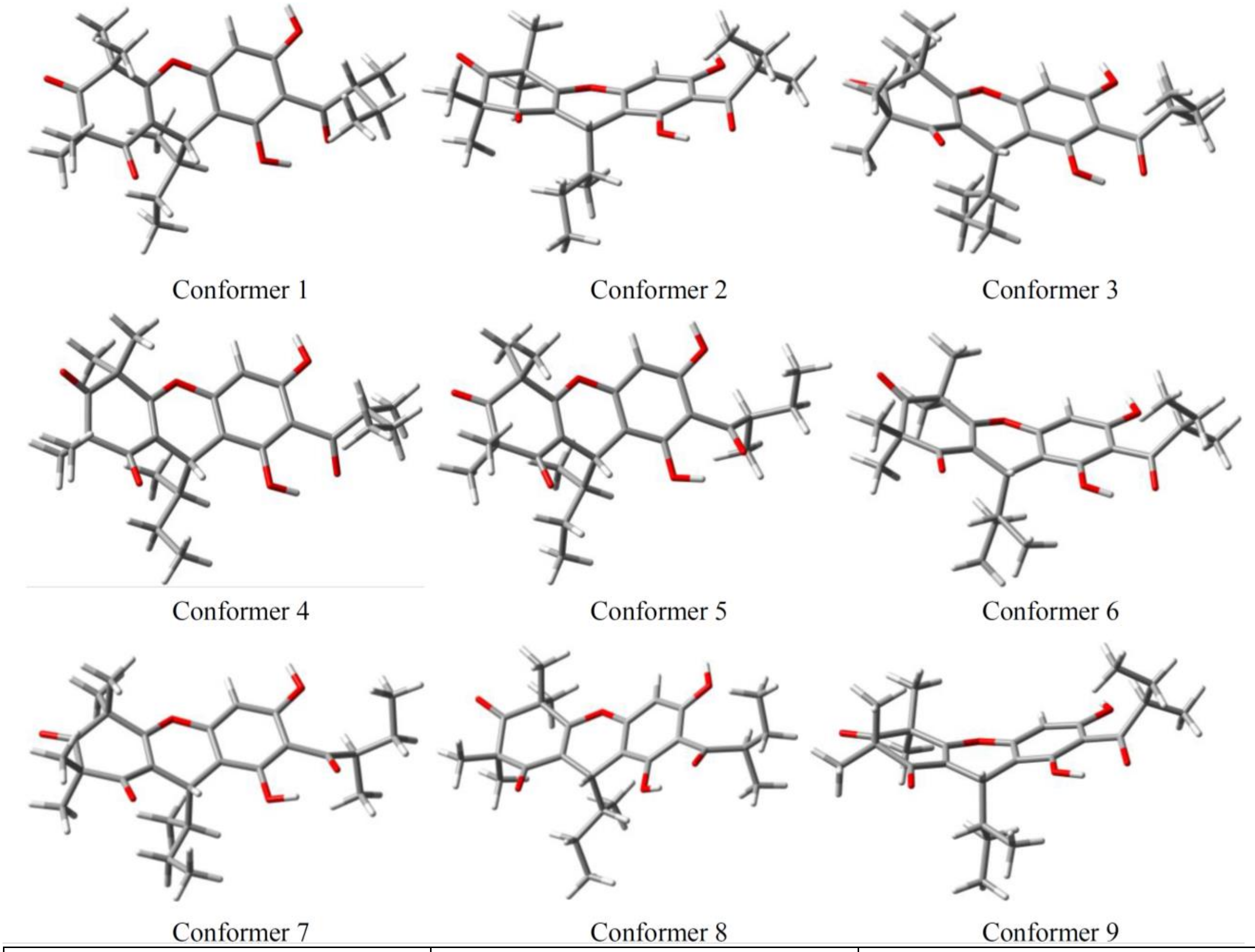

Conformer No.	Distribution (%)	Relative Energy (kcal/mol)
1	14.1897	0.0000
2	11.9192	0.1033
3	11.1017	0.1454
4	8.1426	0.3291
5	3.9477	0.7580
6	3.2785	0.8681
7	3.0370	0.9134
8	3.0324	0.9143
9	2.9531	0.9300

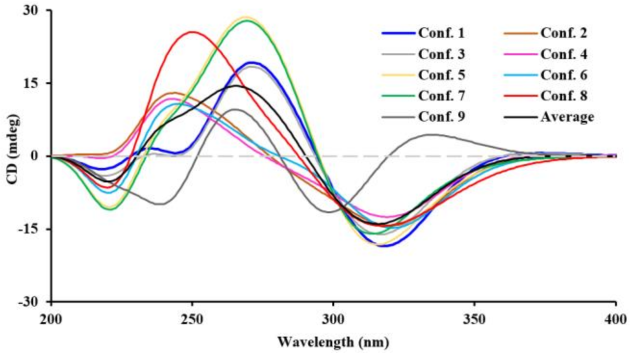

^{13}C NMR calculated data for 7*S*,8*R*-**3**

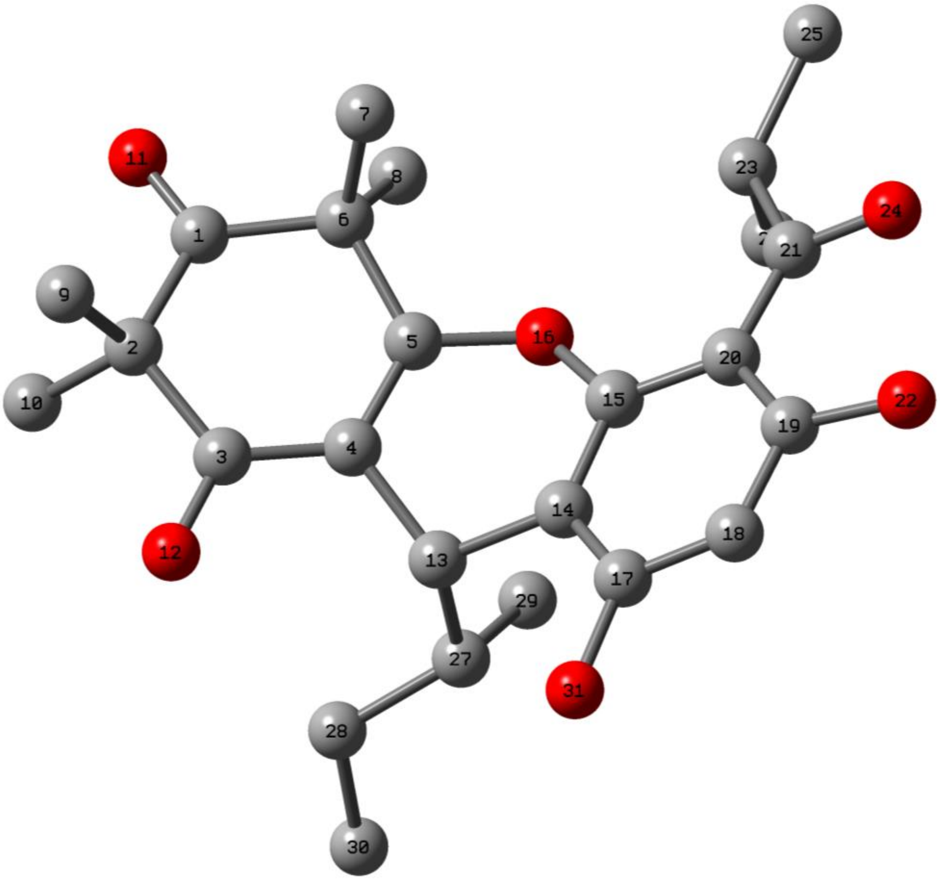

10-C	28.21672
13-C	35.3502
14-C	103.1963
15-C	153.0052
17-C	157.2542
18-C	97.72213
19-C	162.8936
1-C	213.535
20-C	104.1406
21-C	206.9101
23-C	43.73737
25-C	20.48321
26-C	22.68189
27-C	45.50072
28-C	29.53228
29-C	15.28046
2-C	61.08792
30-C	14.51577
3-C	195.9956
4-C	110.9265
5-C	167.7197
6-C	52.266
7-C	26.9991
8-C	26.53058
9-C	24.70381

^{13}C NMR calculated data for 7*S*,8*S*-**3**

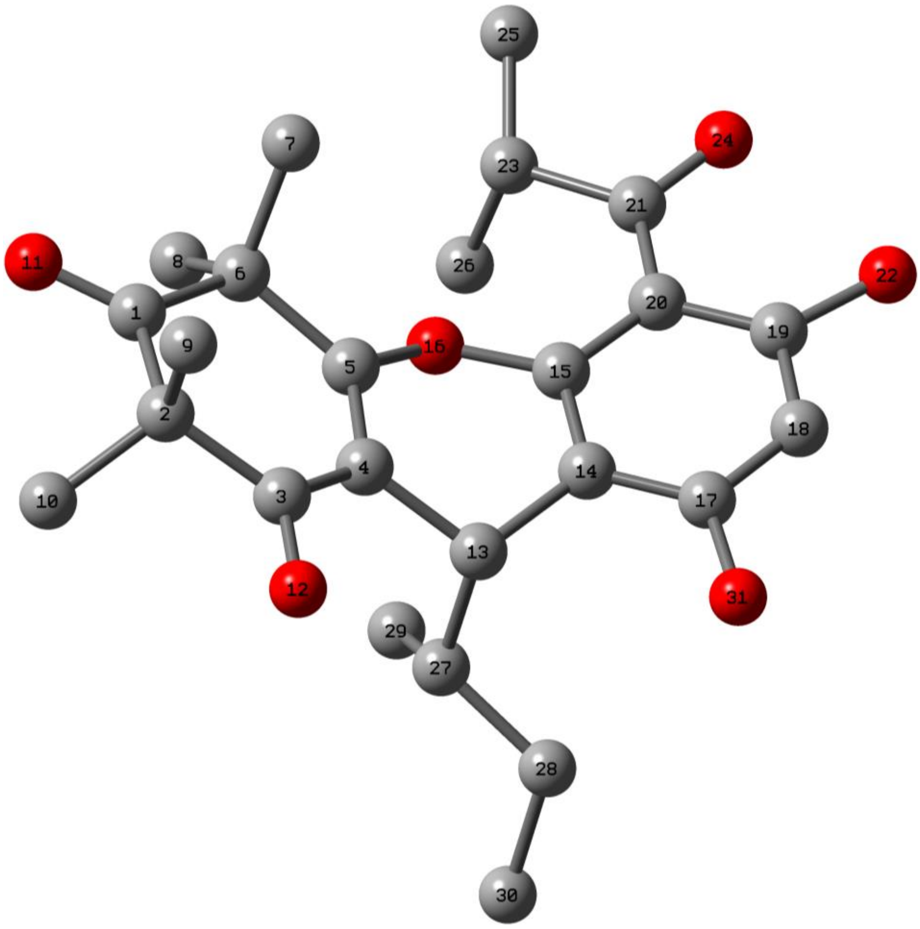

10-C	26.8485
13-C	36.43156
14-C	102.9621
15-C	153.2689
17-C	157.0433
18-C	97.66078
19-C	162.8808
1-C	213.5383
20-C	103.8688
21-C	207.053
23-C	43.73342
25-C	20.66383
26-C	22.39742
27-C	45.05057
28-C	26.70327
29-C	17.54089
2-C	61.09988
30-C	14.0581
3-C	195.9515
4-C	111.1131
5-C	167.6474
6-C	52.17666
7-C	27.09848
8-C	26.52503
9-C	25.98837

^{13}C NMR calculated data for 7*R*,8′*S*-**4**

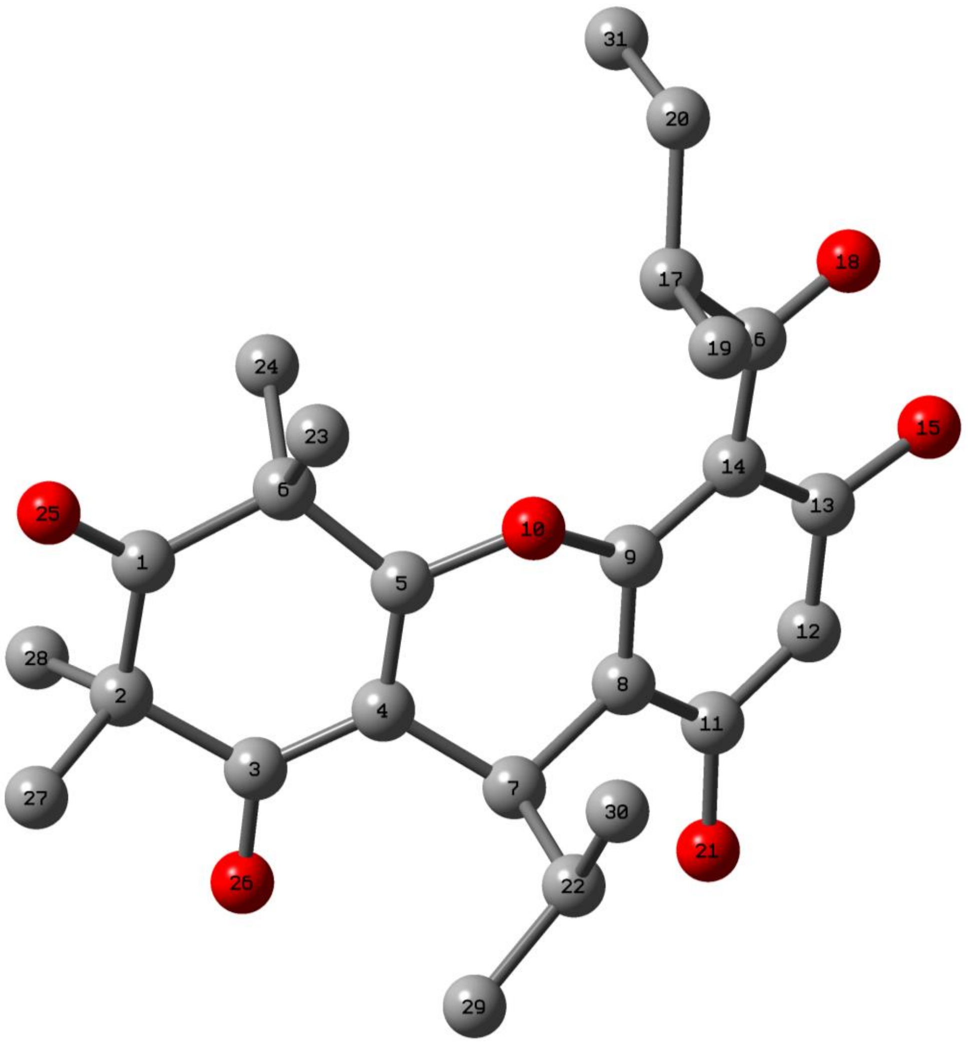

10-C	157.2508
11-C	97.77923
12-C	163.7472
13-C	103.815
14-C	206.1863
15-C	50.00081
16-C	21.42847
17-C	213.3728
18-C	28.2025
19-C	38.59927
1-C	27.57399
20-C	26.50472
21-C	27.20854
22-C	25.29117
23-C	20.76951
24-C	61.86066
25-C	18.46103
2-C	14.36473
3-C	195.6497
4-C	111.5438
5-C	168.0105
6-C	52.2493
7-C	35.90297
8-C	102.9245
9-C	153.0421

^{13}C NMR calculated data for 7*R*,8′*R*-**4**

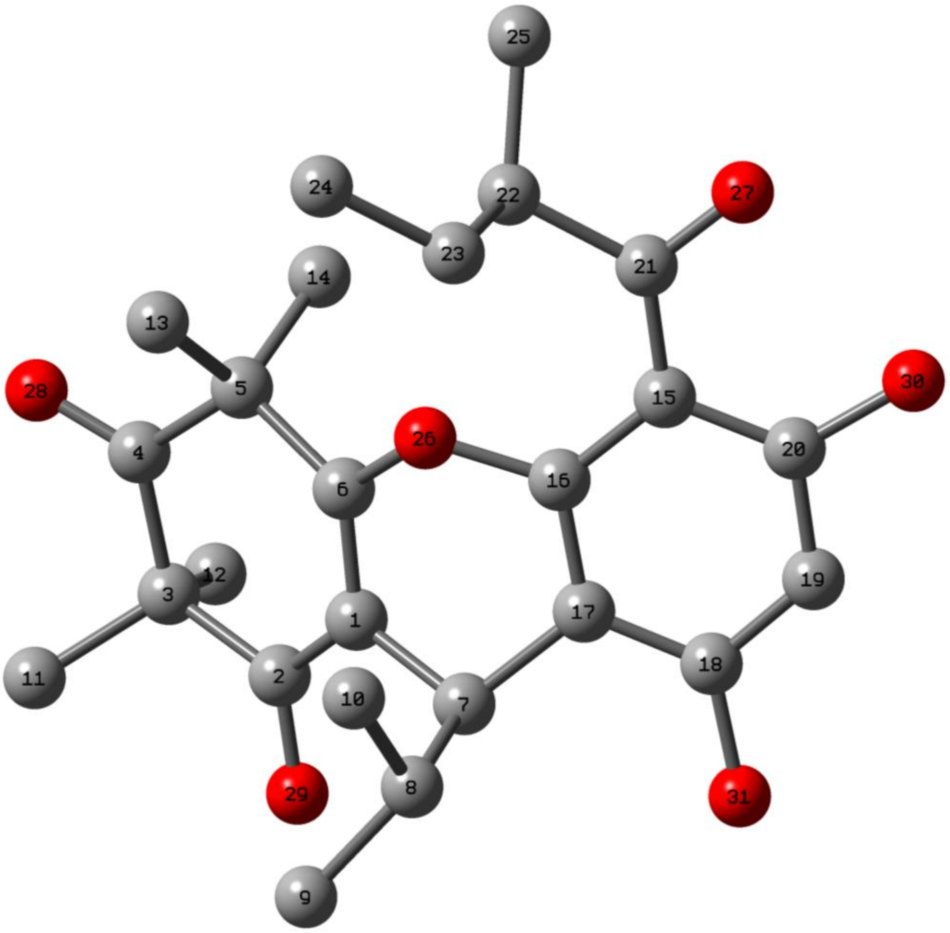

10-C	18.37493
11-C	27.44594
12-C	23.52023
13-C	25.93072
14-C	25.62015
15-C	106.1105
16-C	151.3129
17-C	101.6836
18-C	154.7077
19-C	95.4823
1-C	109.8229
20-C	157.491
21-C	206.3318
22-C	48.43534
23-C	33.51952
24-C	13.71115
25-C	20.22526
2-C	193.1172
3-C	59.87164
4-C	210.1792
5-C	50.26944
6-C	164.8006
7-C	35.45296
8-C	37.40633
9-C	19.9773

^{13}C NMR calculated data for 7*S*,8′*S*-**5**

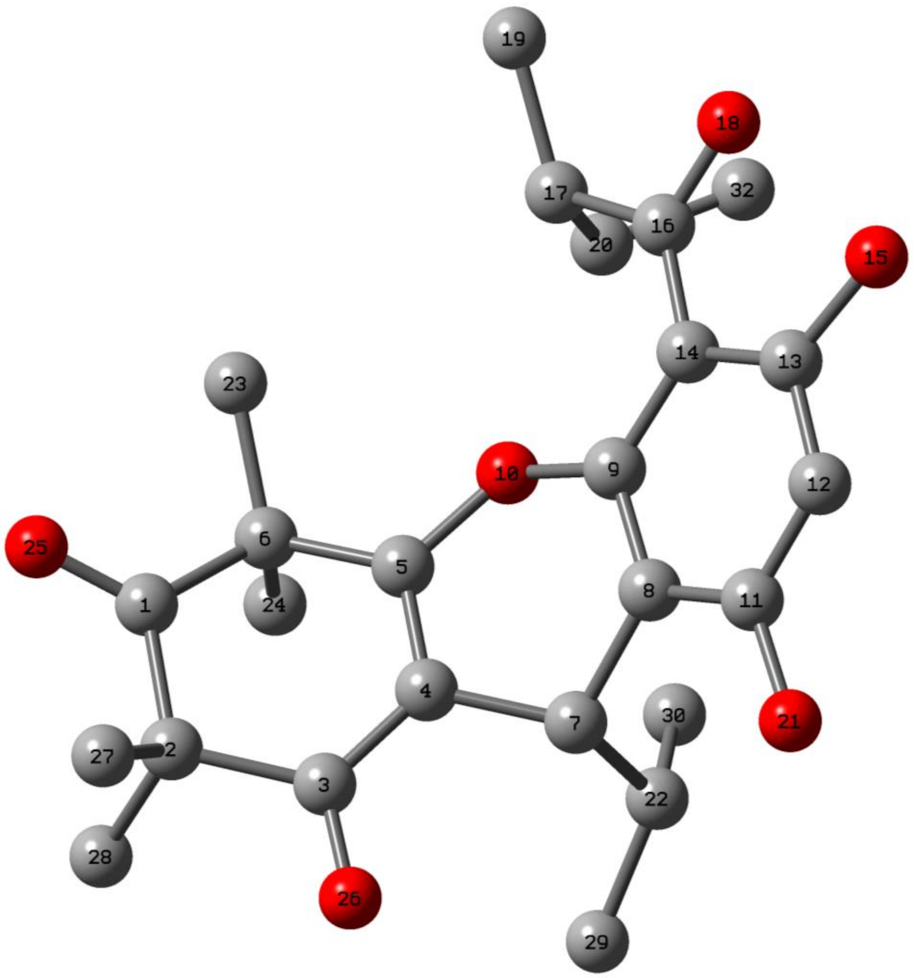

11-C	157.233
12-C	97.19004
13-C	161.3836
14-C	107.1109
16-C	207.8901
17-C	48.93515
19-C	20.88706
1-C	211.8138
20-C	34.66954
22-C	38.97636
23-C	26.52535
24-C	27.31239
27-C	24.54704
28-C	27.97954
29-C	20.85252
2-C	62.19649
30-C	19.21117
31-C	15.32869
3-C	194.964
4-C	110.8421
5-C	167.1772
6-C	52.17048
7-C	36.33127
8-C	102.7972
9-C	153.6282

^{13}C NMR calculated data for 7*S*,8′*R*-**5**

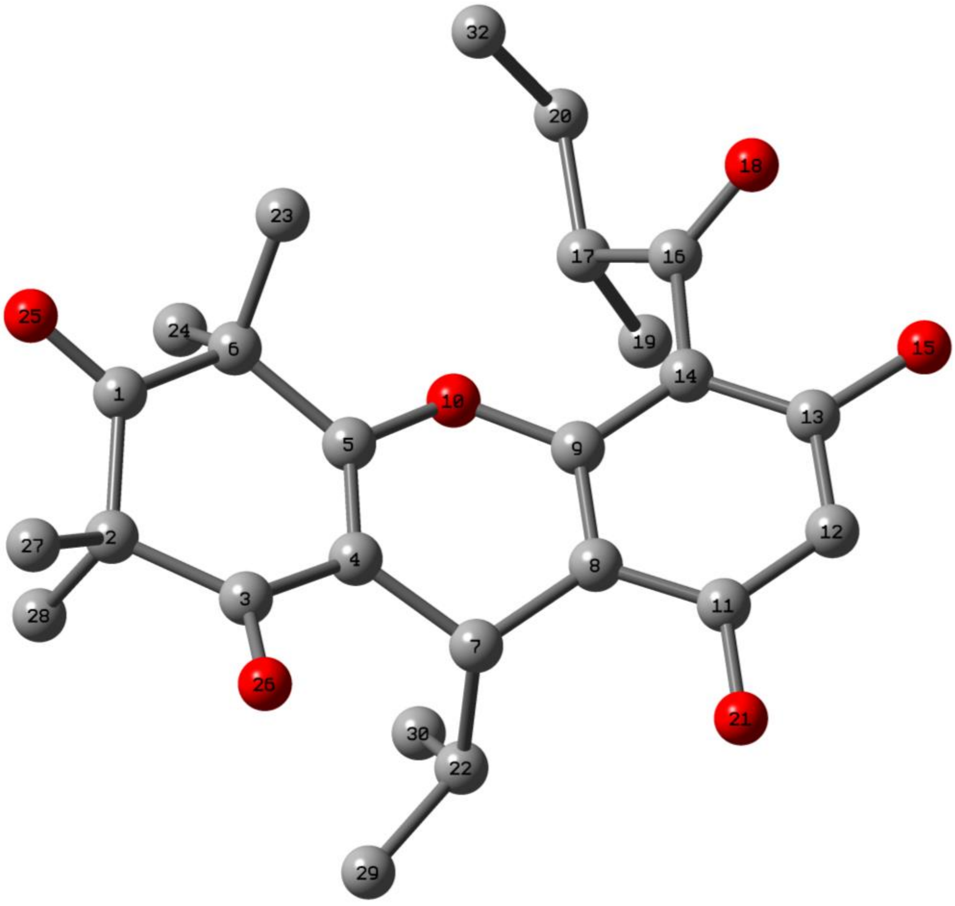

11-C	155.3126
12-C	98.61703
13-C	158.2543
14-C	106.5498
16-C	209.4963
17-C	50.42045
19-C	20.44624
1-C	216.8672
20-C	28.43564
22-C	38.69418
23-C	26.05078
24-C	26.79137
27-C	24.97717
28-C	27.456
29-C	21.7824
2-C	59.73431
30-C	19.08472
31-C	14.86686
3-C	197.4147
4-C	113.8119
5-C	168.0814
6-C	51.92476
7-C	36.99725
8-C	104.5143
9-C	152.0198

^{13}C NMR calculated data for 7*R*,8*S*,8′*S*-**6**

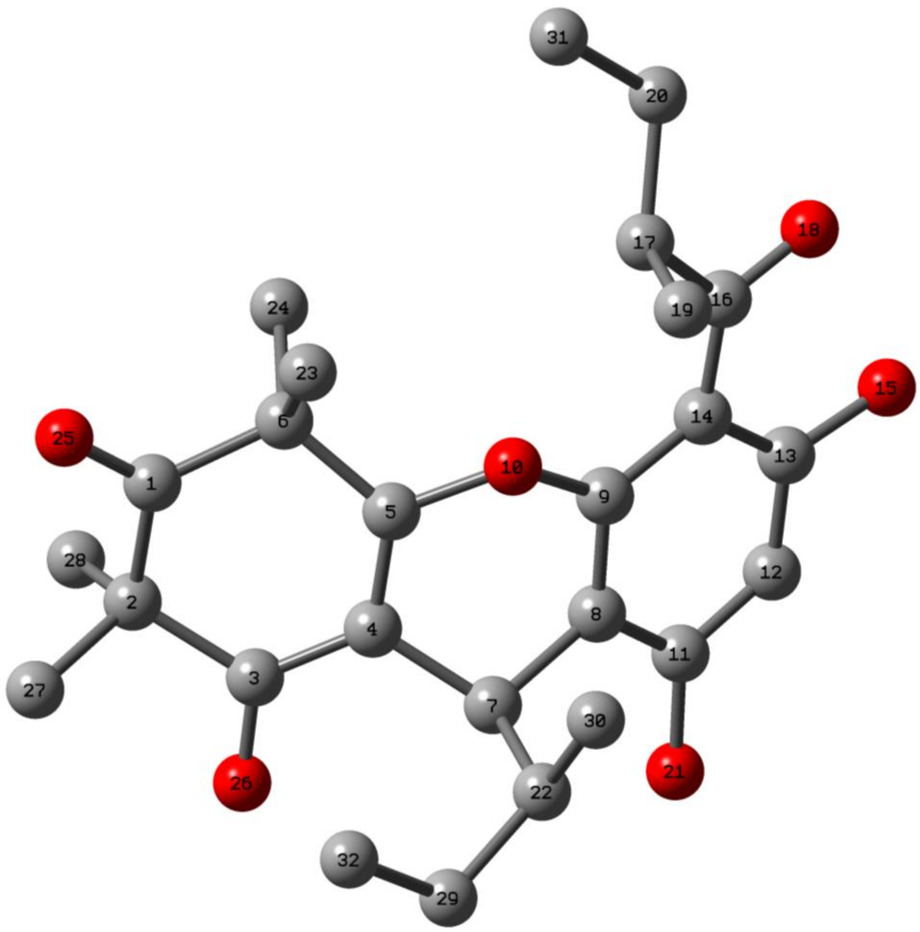

11-C	157.1575
12-C	97.86211
13-C	163.7533
14-C	104.06
16-C	206.0419
17-C	50.11034
19-C	21.56927
1-C	213.3237
20-C	28.13624
22-C	45.43291
23-C	27.44019
24-C	26.50452
27-C	27.51405
28-C	24.97622
29-C	29.47231
2-C	61.82155
30-C	15.5979
31-C	14.50225
32-C	14.58863
3-C	195.9257
4-C	111.5312
5-C	167.8879
6-C	52.1634
7-C	35.16154
8-C	103.4807
9-C	152.8972

^{13}C NMR calculated data for 7*R*,8*R*,8′*R*-**6**

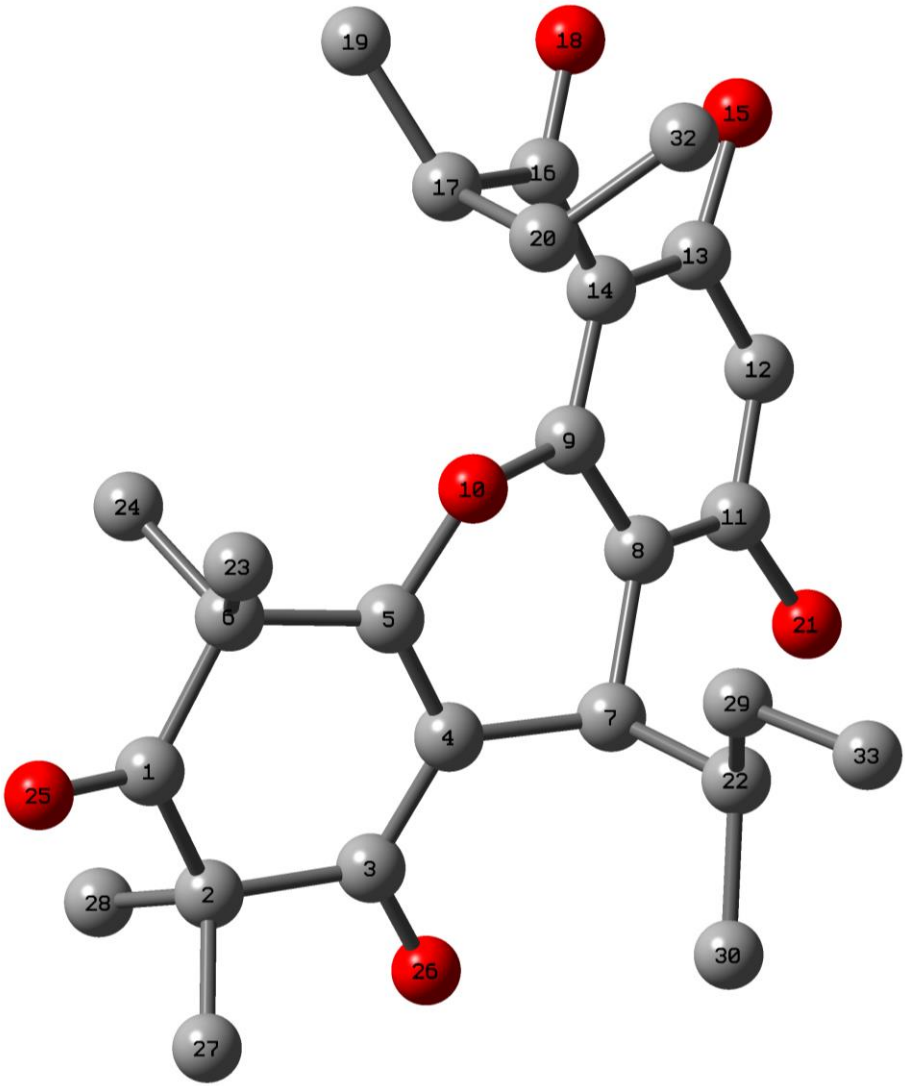

11-C	157.0719
12-C	97.53554
13-C	161.5322
14-C	106.6442
16-C	208.0736
17-C	48.80002
19-C	20.301
1-C	213.2497
20-C	34.12774
22-C	45.23473
23-C	27.20734
24-C	26.04898
27-C	28.57145
28-C	23.62807
29-C	26.94315
2-C	62.07863
30-C	17.318
31-C	14.47974
32-C	14.05299
3-C	195.9176
4-C	111.4093
5-C	166.7693
6-C	51.92355
7-C	36.65404
8-C	102.7849
9-C	153.4954

^{13}C NMR calculated data for 7*R*,8*S*,8′*R*-**6**

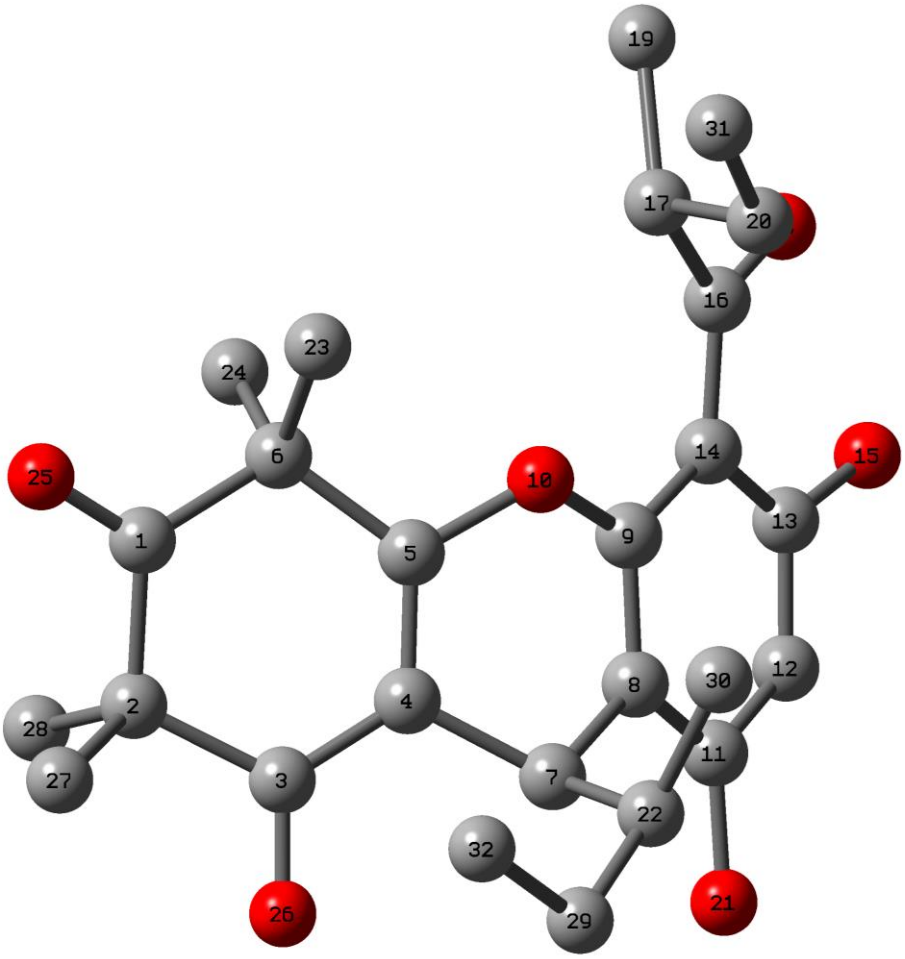

11-C	157.2841798
12-C	97.15117724
13-C	161.3323078
14-C	107.2212002
16-C	208.1431681
17-C	48.74099453
19-C	21.02386171
1-C	213.2690081
20-C	34.35368207
22-C	45.73008975
23-C	26.87352044
24-C	26.53385052
27-C	30.62982523
28-C	21.96103017
29-C	29.23668871
2-C	61.66902593
30-C	15.92121561
31-C	14.95933468
32-C	14.48651118
3-C	196.0068438
4-C	110.9768756
5-C	167.1439576
6-C	52.13890988
7-C	35.37884877
8-C	102.9157618
9-C	153.5550781

^{13}C NMR calculated data for 7*R*,8*R*,8′*S*-**6**

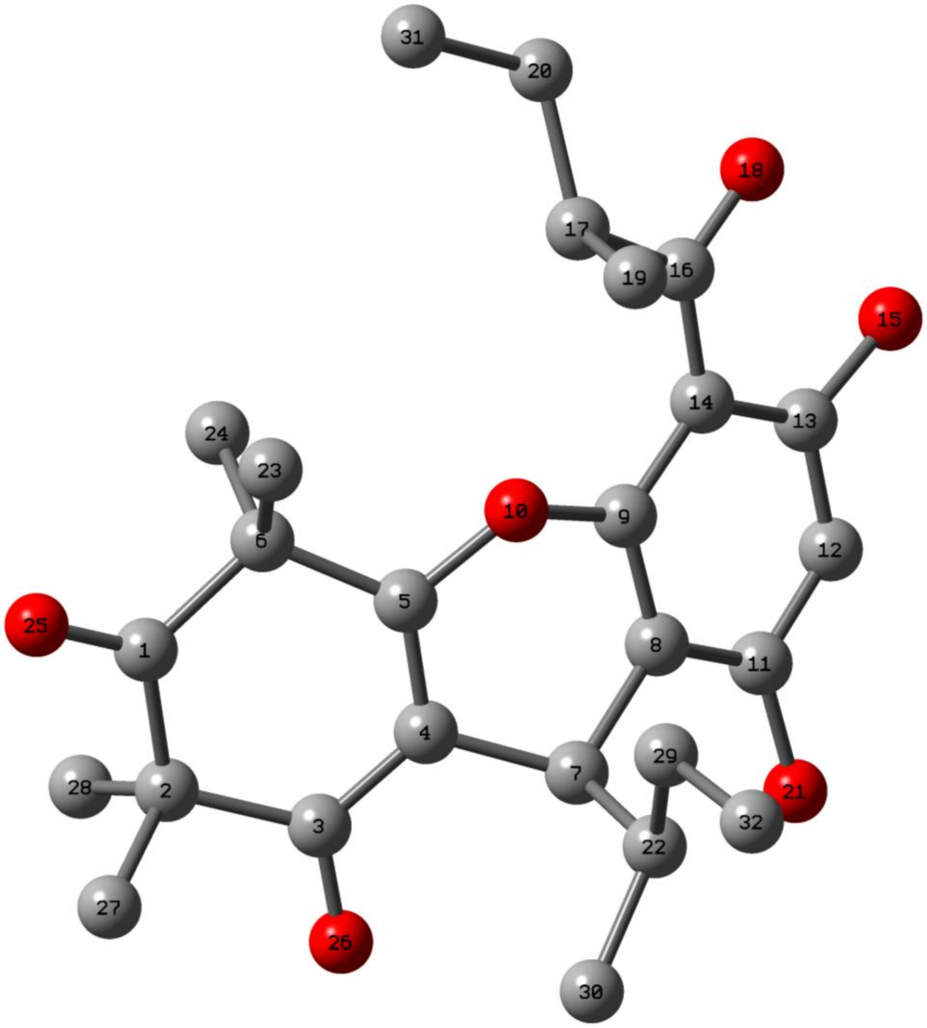

11-C	157.0988
12-C	97.96295
13-C	163.5198
14-C	104.0717
16-C	206.1368
17-C	50.37671
19-C	21.88576
1-C	213.3391
20-C	27.90118
22-C	44.92946
23-C	27.67901
24-C	26.50044
27-C	28.00238
28-C	24.52942
29-C	26.45478
2-C	61.78244
30-C	17.8031
31-C	14.53201
32-C	13.95134
3-C	195.8556
4-C	111.3665
5-C	167.499
6-C	52.19715
7-C	36.62597
8-C	103.1216
9-C	152.8622

^{13}C NMR calculated data for 7*S*,8*R*,8′*S*-**7**

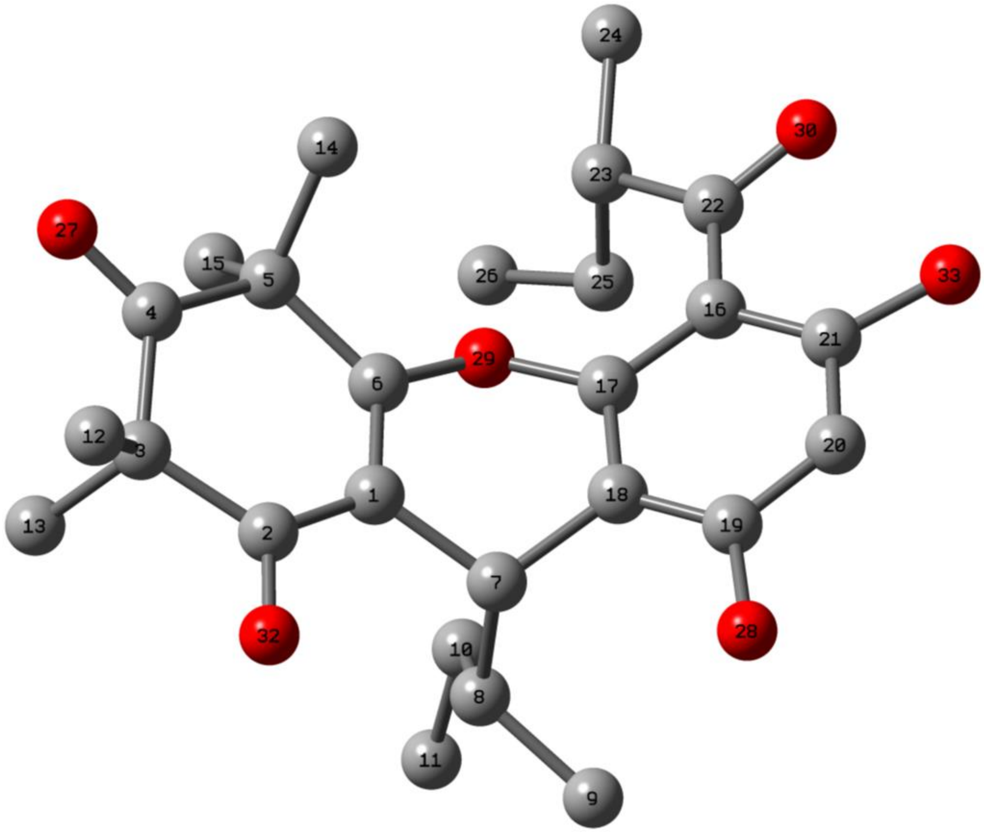

10-C	29.14327
11-C	14.43997
12-C	23.7169
13-C	28.80054
14-C	26.49076
15-C	26.9104
16-C	107.1212
17-C	153.5615
18-C	102.8584
19-C	157.3045
1-C	111.0285
20-C	97.13736
21-C	161.3763
22-C	208.1857
23-C	48.75015
24-C	20.8657
25-C	34.28857
26-C	14.91705
2-C	195.974
3-C	61.65191
4-C	213.4459
5-C	52.12451
6-C	167.028
7-C	35.38422
8-C	45.68521
9-C	15.93978

^{13}C NMR calculated data for 7*S*,8*R*,8′*R*-**7**

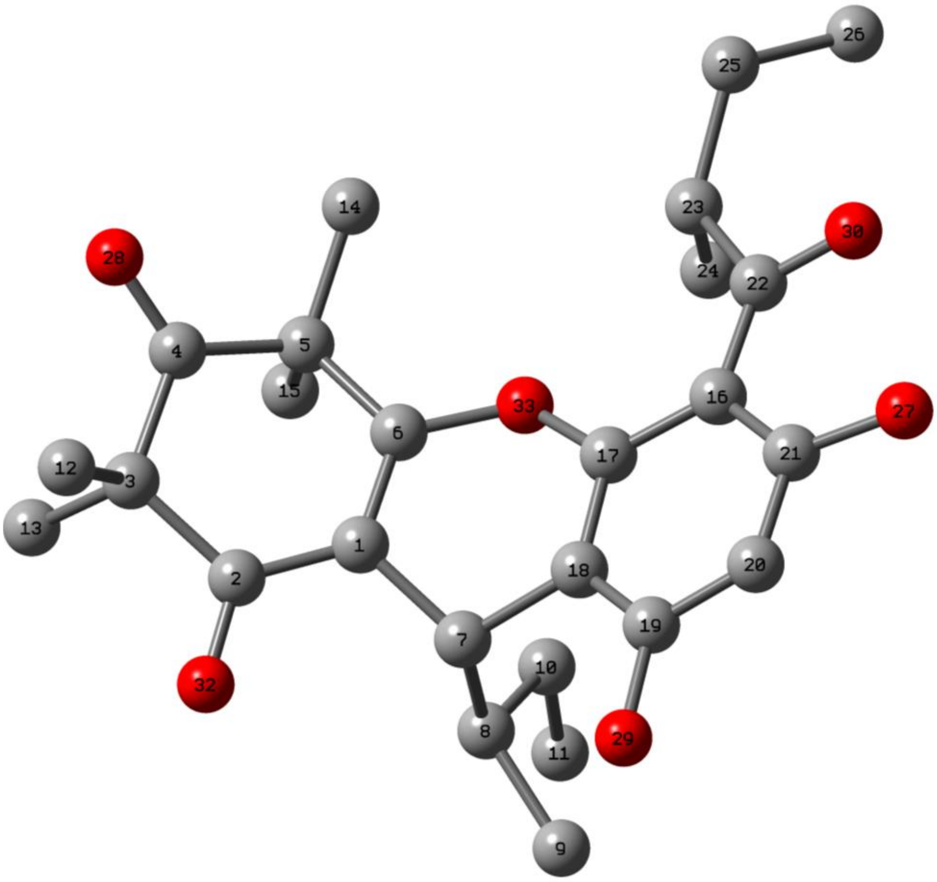

10-C	29.44778
11-C	14.59132
12-C	24.96635
13-C	27.51592
14-C	26.55163
15-C	27.36146
16-C	104.1093
17-C	152.9322
18-C	103.5028
19-C	157.1441
1-C	111.5321
20-C	97.80316
21-C	163.6181
22-C	205.9925
23-C	50.18871
24-C	21.59403
25-C	28.00103
26-C	14.49019
2-C	195.9403
3-C	61.80546
4-C	213.333
5-C	52.1458
6-C	167.9176
7-C	35.17646
8-C	45.42391
9-C	15.58464

^{13}C NMR calculated data for 7*S*,8*S*,8′*R*-**7**

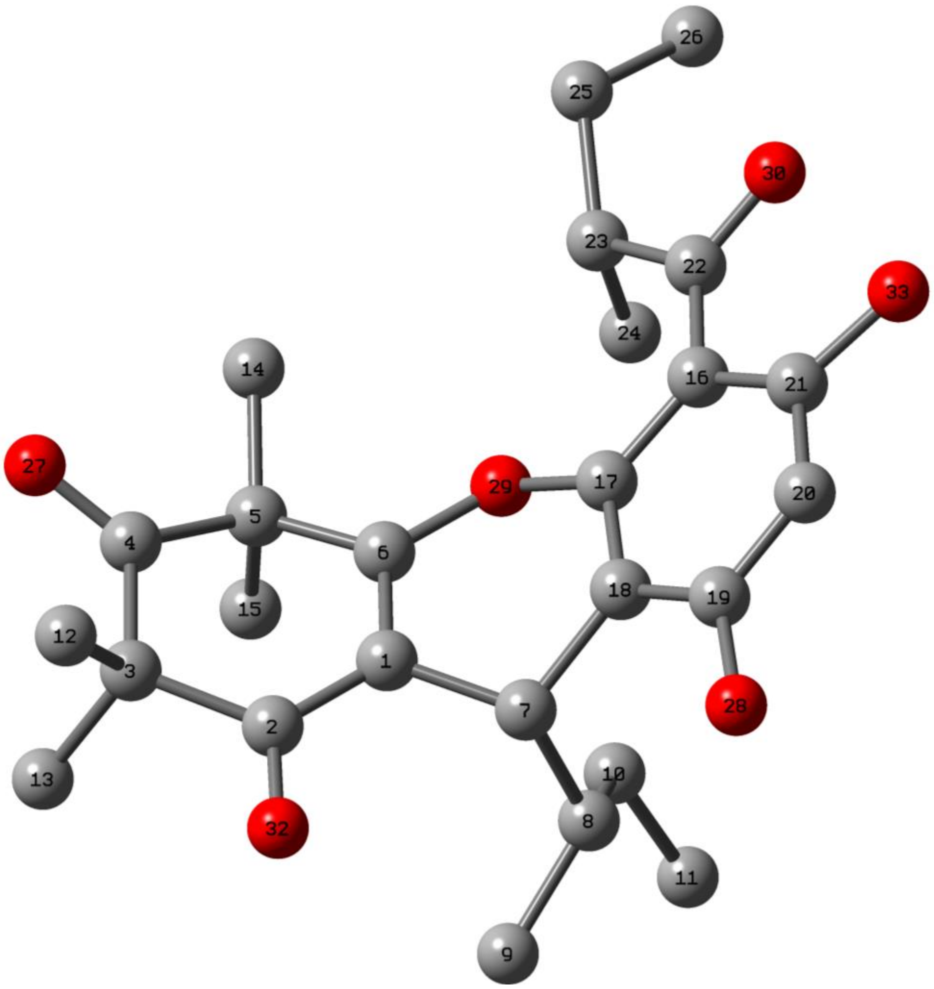

10-C	26.4671
11-C	13.94562
12-C	24.55567
13-C	28.05017
14-C	26.38398
15-C	27.74263
16-C	103.9353
17-C	152.8425
18-C	103.2016
19-C	157.1172
1-C	111.3504
20-C	97.99929
21-C	163.7837
22-C	205.9105
23-C	50.24086
24-C	21.8555
25-C	28.04134
26-C	14.49723
2-C	195.8516
3-C	61.72053
4-C	213.3345
5-C	52.24918
6-C	167.5736
7-C	36.61066
8-C	44.94472
9-C	17.8083

^{13}C NMR calculated data for 7*S*,8*S*,8′*S*-**7**

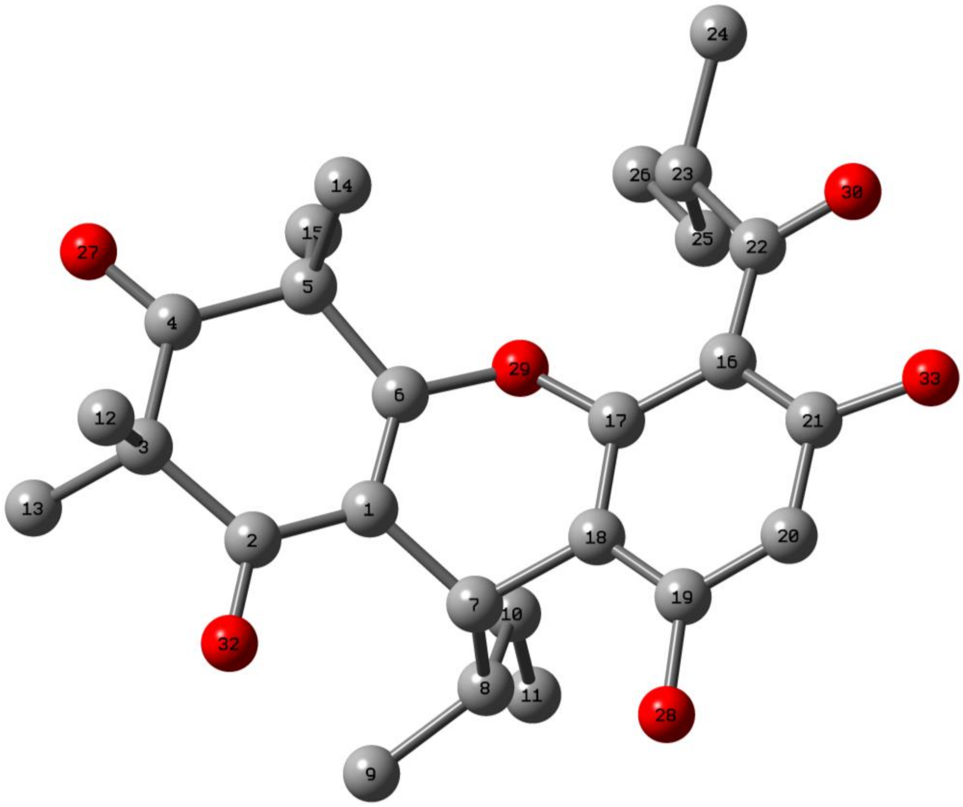

10-C	27.08682
11-C	14.08629
12-C	21.62565
13-C	30.59352
14-C	26.20797
15-C	27.0487
16-C	107.0701
17-C	153.5951
18-C	102.7417
19-C	157.1514
1-C	111.4068
20-C	97.46758
21-C	161.3542
22-C	208.4301
23-C	48.71808
24-C	21.0304
25-C	34.59863
26-C	15.03102
2-C	195.8579
3-C	62.00122
4-C	213.2589
5-C	51.91614
6-C	166.8602
7-C	36.63151
8-C	45.27681
9-C	17.1416

^{13}C NMR calculated data for 7*S*,8*R*,8′*S*-**8**

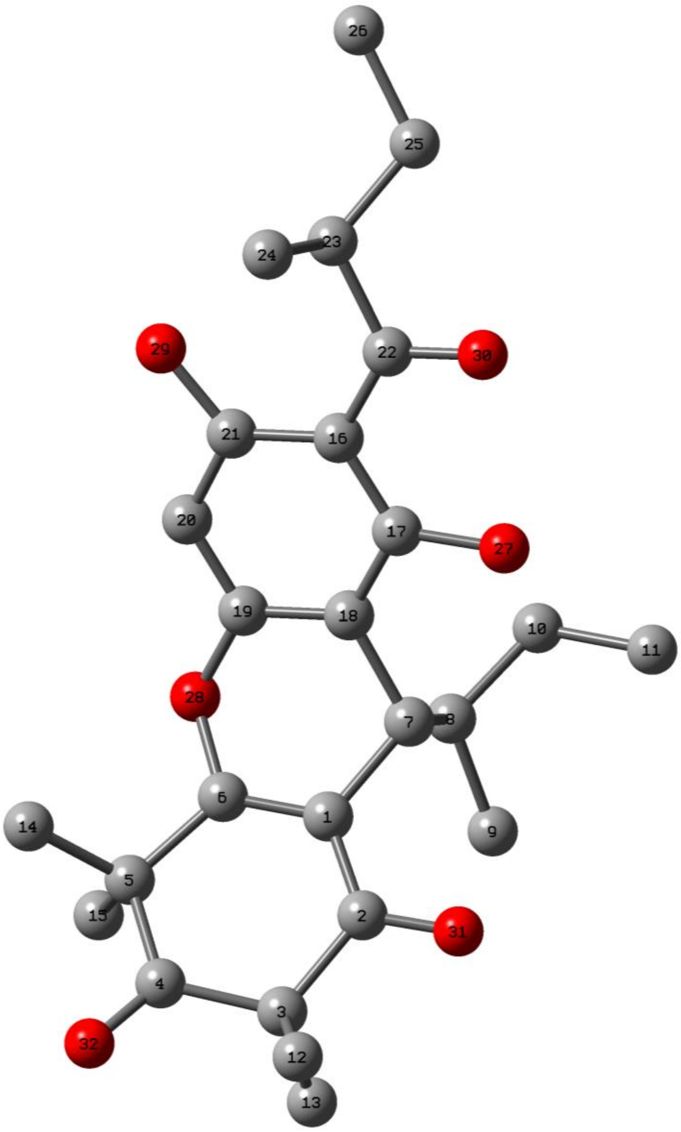

10-C	30.40533
11-C	14.66734
12-C	27.09164
13-C	25.522
14-C	26.24294
15-C	27.07757
16-C	106.8966
17-C	162.1918
18-C	106.3372
19-C	154.7621
1-C	111.0385
20-C	91.24709
21-C	155.8482
22-C	208.1614
23-C	50.22004
24-C	21.80232
25-C	30.36705
26-C	14.50958
2-C	195.803
3-C	61.7312
4-C	213.4698
5-C	51.9898
6-C	168.3561
7-C	35.08617
8-C	45.20469
9-C	15.37308

^{13}C NMR calculated data for 7*S*,8*R*,8′*R*-**8**

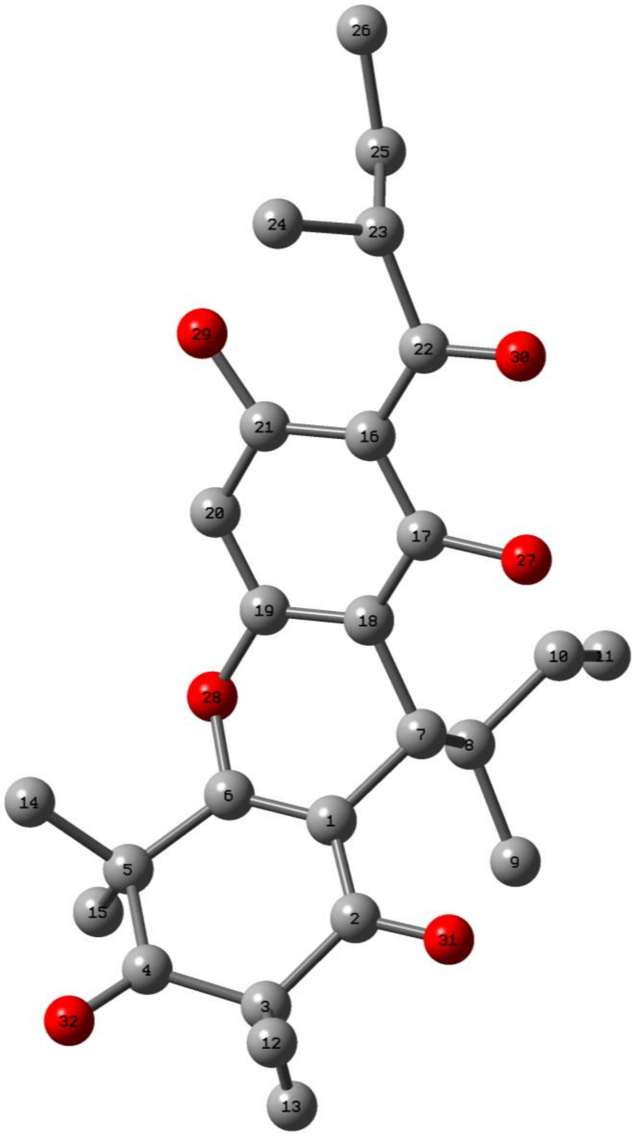

10-C	30.57553
11-C	14.68568
12-C	26.69147
13-C	25.85498
14-C	26.21329
15-C	27.04644
16-C	107.1328
17-C	162.4311
18-C	106.604
19-C	154.8415
1-C	111.1627
20-C	91.44664
21-C	155.8262
22-C	208.1739
23-C	50.24229
24-C	22.11776
25-C	30.12822
26-C	14.36743
2-C	195.9118
3-C	61.80446
4-C	213.4334
5-C	51.98144
6-C	168.4066
7-C	34.9717
8-C	45.05432
9-C	15.35563

^{13}C NMR calculated data for 7*S*,8*S*,8′*R*-**8**

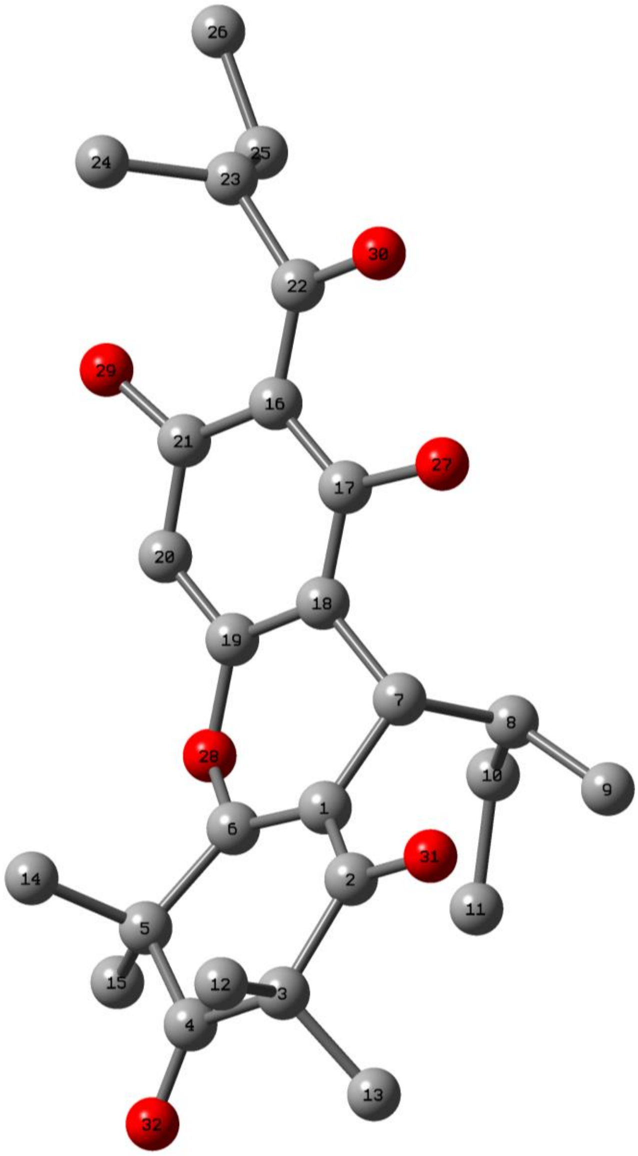

10-C	25.78274
11-C	13.91998
12-C	26.79872
13-C	25.93706
14-C	26.06204
15-C	27.03831
16-C	107.2378
17-C	162.3669
18-C	106.3491
19-C	154.6981
1-C	111.2449
20-C	91.67026
21-C	155.934
22-C	208.2517
23-C	50.14111
24-C	21.36
25-C	30.62879
26-C	14.32084
2-C	195.648
3-C	61.76771
4-C	213.4722
5-C	52.00723
6-C	168.1016
7-C	36.66979
8-C	44.1241
9-C	19.16233

^{13}C NMR calculated data for 7*S*,8*S*,8′*S*-**8**

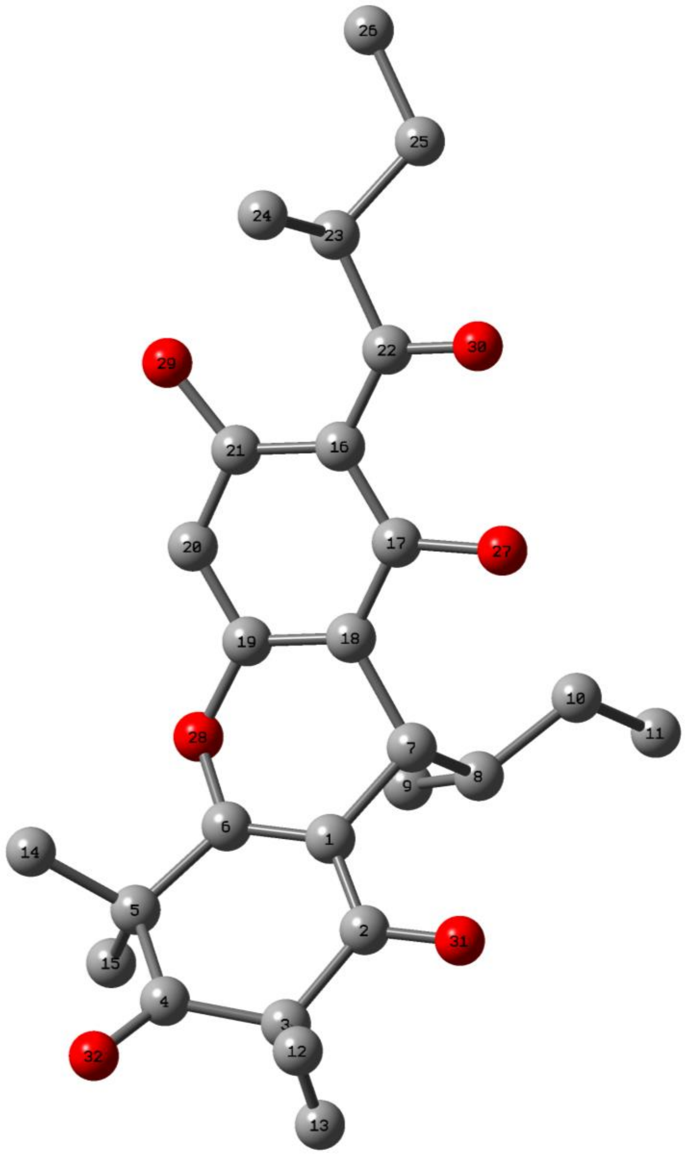

10-C	26.80428
11-C	14.4808
12-C	26.55809
13-C	26.05099
14-C	26.01086
15-C	27.29398
16-C	106.7797
17-C	161.9777
18-C	106.4449
19-C	154.9832
1-C	111.8702
20-C	91.4706
21-C	155.7963
22-C	207.6092
23-C	50.37046
24-C	22.51379
25-C	30.64487
26-C	14.65009
2-C	194.6491
3-C	62.20508
4-C	212.0067
5-C	51.99005
6-C	167.8862
7-C	36.94807
8-C	44.59746
9-C	18.6095

DP4+ possibility analysis of **3**

	A	B	C	D	E	F	G	H
1	Functional		Solvent?		Basis Set		Type of Data	
2	mP▼1P▼91		PC▮		6-31+G(d,p)		Unscaled Shifts	
3								
12			DP4+	98.28%	1.72%	-	-	-
14	Nuclei	sp2?	xperimenta	Isomer 1	Isomer 2	Isomer 3	Isomer 4	Isomer 5
15	C		24.4554	26.8	28.2			
16	C		30.9154	36.4	35.4			
17	C	x	102.9676	103.0	103.2			
18	C	x	153.8003	153.3	153.0			
19	C	x	159.2701	157.0	157.3			
20	C	x	100.5501	97.7	97.7			
21	C	x	164.6401	162.9	162.9			
22	C	x	211.7389	213.5	213.5			
23	C	x	104.0157	103.9	104.1			
24	C	x	208.9495	207.1	206.9			
25	C		39.703	43.7	43.7			
26	C		17.6811	20.66	20.48			
27	C		20.8186	22.40	22.68			
28	C		42.0729	45.05	45.50			
29	C		26.1378	26.70	29.53			
30	C		14.8527	17.54	15.28			
31	C		56.2015	61.10	61.09			
32	C		12.2906	14.06	14.52			
33	C	x	197.8759	195.95	196.00			
34	C	x	112.709	111.11	110.93			
35	C	x	167.5108	167.65	167.72			
36	C		47.1893	52.18	52.27			
37	C		25.0904	27.10	27.00			
38	C		24.9171	26.53	26.53			
39	C		24.3166	25.99	24.70			
40								
41	H		1.4218	1.4655	1.4371			
42	H		1.6064	1.6314	1.6357			
43	H		1.3856	1.3335	1.3181			
44	H		1.4309	1.4441	1.4382			
45	H		4.3457	4.4827	4.4946			
46	H	x	6.26	6.3691	6.3803			
47	H		13.3047	13.8873	13.9109			
48	H		3.8951	4.0105	3.9989			
49	H		1.2553	1.2664	1.3139			
50	H		1.2414	1.2225	1.2293			
51	H		1.56	1.7253	1.7029			
52	H		1.0261	1.0853	0.8434			
53	H		1.4958	1.3337	1.5009			
54	H		0.738	0.8033	0.8197			
55	H		0.896	0.9466	0.9514			

Functional	Solvent?		Basis Set		Type of Data	
mP▼1P▼91	PC▮		6-31+G(d,p)		Unscaled Shifts	
	Isomer 1	Isomer 2	Isomer 3	Isomer 4	Isomer 5	Isomer 6
sDP4+ (H data)	49.85%	50.15%	-	-	-	-
sDP4+ (C data)	84.64%	15.36%	-	-	-	-
sDP4+ (all data)	84.56%	15.44%	-	-	-	-
uDP4+ (H data)	51.76%	48.24%	-	-	-	-
uDP4+ (C data)	90.69%	9.31%	-	-	-	-
uDP4+ (all data)	91.27%	8.73%	-	-	-	-
DP4+ (H data)	51.61%	48.39%	-	-	-	-
DP4+ (C data)	98.17%	1.83%	-	-	-	-
DP4+ (all data)	98.28%	1.72%	-	-	-	-

DP4+ analysis of **4**

Functional		Solvent?		Basis Set		Type of Data	
mPW1PW91		PCM		6-31+G(d,p)		Unscaled Shifts	
		DP4+	0.00%	100.00%	–	–	–
Nuclei	sp2?	xperimenta	Isomer 1	Isomer 2	Isomer 3	Isomer 4	Isomer 5
C		18.6842	18.4	18.5			
C		25.1158	27.4	27.2			
C		24.1756	23.5	25.3			
C		24.9572	25.9	27.6			
C		24.8485	25.6	26.5			
C	x	104.1667	106.1	103.8			
C	x	153.475	151.3	153.0			
C	x	103.5873	101.7	102.9			
C	x	159.2874	154.7	157.3			
C	x	100.6641	95.5	97.8			
C	x	112.1428	109.8	111.5			
C	x	164.9532	157.49	163.75			
C	x	208.6712	206.33	206.19			
C		46.842	48.44	50.00			
C		24.8485	33.52	28.20			
C		12.1437	13.71	14.36			
C		18.8081	20.23	21.43			
C	x	198.3647	193.12	195.65			
C		56.1668	59.87	64.86			
C	x	211.7774	210.18	213.37			
C		47.285	50.27	52.25			
C	x	167.9925	164.80	168.01			
C		31.4748	35.45	35.90			
C		34.7982	37.41	38.60			
C		18.8173	19.98	20.77			
H		4.3478	4.2872	4.4945			
H	x	6.2916	6.1957	6.5146			
H		3.7572	3.6377	4.0868			
H		1.9103	1.6594	2.1023			
H		1.4993	1.3464	1.5576			
H		1.222	1.1977	1.2596			
H		1.9103	1.9359	2.1023			
H		0.8289	0.6902	0.804			
H		0.7861	0.6785	0.7726			
H		0.9821	0.5302	1.0549			
H		1.628	1.5425	1.7347			
H		1.4449	1.2743	1.5365			
H		1.4529	1.332	1.4785			
H		1.3929	1.163	1.3511			
H		13.54	11.79	14.5194			

Functional	Solvent?		Basis Set		Type of Data	
mPW1PW91	PCM		6-31+G(d,p)		Unscaled Shifts	
	Isomer 1	Isomer 2	Isomer 3	Isomer 4	Isomer 5	Isomer 6
sDP4+ (H data)	0.00%	100.00%	–	–	–	–
sDP4+ (C data)	0.02%	99.98%	–	–	–	–
sDP4+ (all data)	0.00%	100.00%	–	–	–	–
uDP4+ (H data)	0.13%	99.87%	–	–	–	–
uDP4+ (C data)	0.00%	100.00%	–	–	–	–
uDP4+ (all data)	0.00%	100.00%	–	–	–	–
DP4+ (H data)	0.00%	100.00%	–	–	–	–
DP4+ (C data)	0.00%	100.00%	–	–	–	–
DP4+ (all data)	0.00%	100.00%	–	–	–	–

DP4+ analysis of **5**

Functional		Solvent?	Basis Set		Type of Data		
mPW1PW91		PCM	6-31+G(d,p)		Unscaled Shifts		
		DP4+	100.00%	0.00%	-	-	-
Nuclei	sp2?	xperimenta	Isomer 1	Isomer 2	Isomer 3	Isomer 4	Isomer 5
C	x	159.4881	157.2	155.3			
C	x	100.4676	97.2	98.6			
C	x	164.087	161.4	158.3			
C	x	105.1294	107.1	106.5			
C	x	209.236	207.9	209.5			
C		45.486	48.9	50.4			
C		15.6046	20.9	20.4			
C	x	211.7512	211.8	216.9			
C		28.5984	34.7	28.4			
C		34.7629	39.0	38.7			
C		24.9412	26.5	26.1			
C		25.1267	27.31	26.79			
C		24.0097	24.55	24.98			
C		25.0556	27.98	27.46			
C		18.7517	20.85	21.78			
C		56.1684	62.20	59.73			
C		18.6433	19.21	19.08			
C		11.2281	15.33	14.87			
C	x	198.6514	194.96	197.41			
C	x	112.0547	110.84	113.81			
C	x	168.1225	167.18	168.08			
C		47.3415	52.17	51.92			
C		31.4763	36.33	37.00			
C	x	103.7295	102.80	104.51			
C	x	153.6001	153.63	152.02			
H		4.3077	4.4469	4.4668			
H	x	6.2659	6.3896	6.4314			
H		13.1266	13.2051	11.2919			
H		3.8851	4.0015	3.7898			
H		1.255	1.3798	1.1786			
H		1.5669	1.5311	2.0518			
H		1.7532	1.8515	1.5372			
H		1.9198	2.0785	2.1409			
H		1.4088	1.4785	1.4454			
H		1.6251	1.7149	1.6904			
H		1.3825	1.3513	1.3587			
H		1.4469	1.5082	1.4856			
H		0.8453	0.8709	0.8306			
H		0.7929	0.8658	0.8163			
H		0.8502	0.7522	1.034			

Functional	Solvent?		Basis Set		Type of Data	
mPW1PW91	PCM		6-31+G(d,p)		Unscaled Shifts	
	Isomer 1	Isomer 2	Isomer 3	Isomer 4	Isomer 5	Isomer 6
sDP4+ (H data)	100.00%	0.00%	-	-	-	-
sDP4+ (C data)	98.06%	1.94%	-	-	-	-
sDP4+ (all data)	100.00%	0.00%	-	-	-	-
uDP4+ (H data)	100.00%	0.00%	-	-	-	-
uDP4+ (C data)	99.81%	0.19%	-	-	-	-
uDP4+ (all data)	100.00%	0.00%	-	-	-	-
DP4+ (H data)	100.00%	0.00%	-	-	-	-
DP4+ (C data)	100.00%	0.00%	-	-	-	-
DP4+ (all data)	100.00%	0.00%	-	-	-	-

DP4+ analysis of **6**

Functional		Solvent?		Basis Set		Type of Data	
mPW1PW91		PCM		6-31+G(d,p)		Unscaled Shifts	
		DP4+	0.01%	0.00%	0.00%	99.99%	-
Nuclei	sp2?	xperimenta	Isomer 1	Isomer 2	Isomer 3	Isomer 4	Isomer 5
C	x	158.9984	157.0933	157.0719	157.2842	157.1575	
C	x	100.6576	97.9628	97.5355	97.1512	97.8621	
C	x	164.9396	163.52	161.5322	161.3323	163.7533	
C	x	104.6614	104.0488	106.6442	107.2212	104.06	
C	x	208.6843	206.1537	208.0736	208.1432	206.0419	
C		46.8313	50.3951	48.80002	48.741	50.1103	
C		18.7904	21.8453	20.30101	21.0239	21.5693	
C	x	211.8213	213.3455	213.2498	213.269	213.3239	
C		24.7667	27.8715	34.1277	34.3537	28.1362	
C		42.3146	44.9255	45.234	45.7301	45.4329	
C		24.9834	27.6488	27.2073	26.8735	27.4402	
C		23.6692	26.5312	26.049	26.5339	26.5045	
C		25.3896	27.8607	28.5715	30.6298	27.5141	
C		25.1336	24.6935	23.6281	21.961	24.9762	
C		26.5792	26.4377	26.9432	29.2367	29.4723	
C		56.1822	61.7548	62.0786	61.669	61.8216	
C		14.6257	17.8143	17.318	15.9212	15.5979	
C		12.1255	14.5157	14.4797	14.9593	14.5023	
C		12.386	13.9482	14.053	14.4865	14.5886	
C	x	198.4293	195.8403	195.9176	196.0069	195.9257	
C	x	111.6174	111.3478	111.4093	110.9769	111.5312	
C	x	167.9279	167.5439	166.7693	167.144	167.8879	
C		47.3587	52.2	51.9236	52.1389	52.1634	
C		30.5377	36.6083	36.654	35.3789	35.1615	
C	x	104.1118	103.1375	102.7849	102.9158	103.4807	
C	x	153.2086	152.8551	153.4954	153.5551	152.8972	
H		4.4142	4.4367	4.4999	4.5123	4.5651	
H	x	6.2648	6.3742	6.3084	6.3458	6.4429	
H		3.7589	4.0022	3.9963	3.9107	4.0269	
H		1.2235	1.2203	1.3139	1.3742	1.2443	
H		1.4985	1.4713	1.5671	1.4789	1.4962	
H		1.9599	2.0567	1.8078	1.8512	2.0918	
H		1.6098	1.7523	1.6534	1.6597	1.7433	
H		1.6296	1.6286	1.7606	1.6944	1.7033	
H		1.4381	1.4729	1.4152	1.4143	1.5142	
H		1.449	1.4603	1.4839	1.4842	1.4671	
H		1.3912	1.2918	1.3151	1.3094	1.3365	
H		0.9198	1.054	1.0718	0.912	0.8682	
H		1.4333	1.3384	1.4722	1.454	1.48	
H		0.7622	0.8382	0.8321	0.8396	0.7808	
H		0.9785	1.0101	0.6885	0.7031	1.0256	
H		0.9166	0.7664	0.8042	0.977	0.9787	

Functional	Solvent?		Basis Set		Type of Data	
mPW1PW91	PCM		6-31+G(d,p)		Unscaled Shifts	
	Isomer 1	Isomer 2	Isomer 3	Isomer 4	Isomer 5	Isomer 6
sDP4+ (H data)	1.32%	0.06%	2.00%	96.62%	-	-
sDP4+ (C data)	7.68%	0.00%	0.00%	92.32%	-	-
sDP4+ (all data)	0.11%	0.00%	0.00%	99.89%	-	-
uDP4+ (H data)	34.43%	0.52%	22.80%	42.26%	-	-
uDP4+ (C data)	8.73%	0.00%	0.00%	91.27%	-	-
uDP4+ (all data)	7.23%	0.00%	0.00%	92.77%	-	-
DP4+ (H data)	1.09%	0.00%	1.09%	97.81%	-	-
DP4+ (C data)	0.79%	0.00%	0.00%	99.21%	-	-
DP4+ (all data)	0.01%	0.00%	0.00%	99.99%	-	-

DP4+ analysis of **7**

Functional	Solvent?	Basis Set	Type of Data
mPW1PW91	PCM	6-31+G(d,p)	Unscaled Shifts

		DP4+	100.00%	0.00%	0.00%	0.00%	-
**Nuclei**	**sp2?**	**xperimenta**	**Isomer 1**	**Isomer 2**	**Isomer 3**	**Isomer 4**	**Isomer 5**
C		26.1347	29.1995	26.4671	27.0868	29.4478	
C		12.2872	14.4487	13.9456	14.0863	14.5913	
C		24.1606	23.8123	24.5557	21.6257	24.9664	
C		24.8978	28.7304	28.0502	30.5935	27.5159	
C		24.6414	26.5176	26.384	26.2078	26.5516	
C		25.0996	26.8586	27.7426	27.0487	27.3615	
C	x	105.327	107.1984	103.9353	107.0701	104.1094	
C	x	153.9657	153.5454	152.8425	153.5952	152.9322	
C	x	103.0569	102.9011	103.2016	102.7417	103.5028	
C	x	159.3046	157.3056	157.1172	157.1514	157.1441	
C	x	112.7012	110.9591	111.3504	111.4068	111.5321	
C	x	100.4173	97.1271	97.9993	97.4676	97.8032	
C	x	163.9792	161.355	163.7837	161.3542	163.6181	
C	x	209.2878	208.2236	205.9105	208.43	205.9925	
C		45.5364	48.7176	50.2409	48.71818	50.1887	
C		15.6183	20.995	21.8555	21.03047	21.594	
C		28.6378	34.3164	28.0413	34.5986	28.0012	
C		11.2638	14.9184	14.4972	15.031	14.4902	
C	x	198.1376	195.992	195.8516	195.85792	195.9403	
C		56.2201	61.6472	61.7205	62.0012	61.8055	
C	x	211.6933	213.4558	213.3345	213.2589	213.333	
C		47.2447	52.1253	52.2492	51.9161	52.14589	
C	x	167.6697	167.0239	167.5736	166.8602	167.9176	
C		30.907	35.3568	36.6107	36.6315	35.1765	
C		42.0655	45.7038	44.9447	45.2768	45.4239	
C		14.7305	15.8776	17.8083	17.1416	15.5846	
H		4.3691	4.5091	4.4516	4.4835	4.5175	
H	x	6.2625	6.3423	6.3919	6.3035	6.3713	
H		13.1157	13.5582	14.2941	13.55	14.3014	
H		3.8772	3.9045	3.9681	3.9737	3.9433	
H		1.2563	1.3363	1.2354	1.3289	1.2267	
H		1.7506	1.8474	2.0383	1.4881	2.0732	
H		1.5593	1.4738	1.4658	1.8368	1.4875	
H		1.5712	1.6904	1.7608	1.7522	1.7204	
H		1.407	1.4738	1.4774	1.4233	1.4869	
H		1.6206	1.6541	1.6731	1.6578	1.6853	
H		1.3855	1.3091	1.31	1.3264	1.3242	
H		1.4399	1.4692	1.4617	1.4663	1.452	
H		1.0409	0.9134	1.05	1.064	0.8615	
H		1.51	1.4693	1.339	1.5007	1.4585	
H		0.7482	0.8333	0.8387	0.824	0.7757	
H		0.8521	0.6937	1.0114	0.6896	1.0112	
H		0.905	0.9709	0.7704	0.8071	0.9698	

Functional	Solvent?	Basis Set	Type of Data
mPW1PW91	PCM	6-31+G(d,p)	Unscaled Shifts

	Isomer 1	Isomer 2	Isomer 3	Isomer 4	Isomer 5	Isomer 6
sDP4+ (H data)	88.34%	0.04%	11.50%	0.12%	-	-
sDP4+ (C data)	38.17%	5.88%	0.25%	55.70%	-	-
sDP4+ (all data)	99.71%	0.01%	0.09%	0.19%	-	-
uDP4+ (H data)	94.08%	0.56%	4.54%	0.82%	-	-
uDP4+ (C data)	31.13%	7.54%	0.33%	61.01%	-	-
uDP4+ (all data)	98.13%	0.14%	0.05%	1.68%	-	-
DP4+ (H data)	99.37%	0.00%	0.62%	0.00%	-	-
DP4+ (C data)	25.66%	0.96%	0.00%	73.39%	-	-
DP4+ (all data)	100.00%	0.00%	0.00%	0.00%	-	-

DP4+ analysis of **8**

Functional		Solvent?	Basis Set		Type of Data	
mPW1PW91		PCM	6-31+G(d,p)		Unscaled Shifts	
		DP4+	0.03%	0.02%	0.06%	99.89%	-
Nuclei	sp2?	xperimenta	Isomer 1	Isomer 2	Isomer 3	Isomer 4	Isomer 5
C		26.1229	25.8	30.6	26.8	30.4	
C		11.9819	13.9	14.7	14.5	14.7	
C		24.7363	26.8	26.7	26.6	27.1	
C		24.1094	25.9	25.9	26.1	25.5	
C		24.5505	26.1	26.2	26.0	26.2	
C		25.0801	27.0	27.0	27.3	27.1	
C	x	107.2631	107.2	107.1	106.8	106.9	
C	x	162.3484	162.4	162.4	162.0	162.2	
C	x	104.4438	106.3	106.6	106.4	106.3	
C	x	156.6284	154.7	154.8	155.0	154.8	
C	x	112.5871	111.24	111.16	111.87	111.04	
C	x	94.9435	91.67	91.45	91.47	91.25	
C	x	158.5051	155.93	155.83	155.80	155.85	
C	x	210.8252	208.25	208.17	207.61	208.16	
C		46.497	50.14	50.24	50.37	50.22	
C		16.5786	21.36	22.12	22.51	21.80	
C		26.8017	30.63	30.13	30.64	30.37	
C		12.3502	14.32	14.37	14.65	14.51	
C	x	197.5523	195.65	195.91	194.65	195.80	
C		56.1802	61.77	61.80	62.21	61.73	
C	x	212.1576	213.47	213.43	212.01	213.47	
C		47.2468	52.01	51.98	51.99	51.99	
C	x	167.5727	168.10	168.41	167.89	168.36	
C		31.389	36.67	34.97	36.95	35.09	
C		41.9396	44.12	45.05	44.59	45.21	
C		15.32	19.16	15.36	18.61	15.37	
H		4.3192	4.4141	4.4717	4.4067	4.4834	
H	x	6.0961	6.0327	6.0505	6.068	6.0587	
H		1.6	1.9711	1.8654	1.9	1.8058	
H		0.7135	0.8501	0.7634	0.6562	0.6436	
H		1.009	0.9293	1.1088	0.8253	0.844	
H		1.441	1.0957	1.0713	1.7029	1.5086	
H		0.8852	0.7323	0.9773	0.8309	0.9514	
H		12.2966	15.2246	15.2223	16.2038	15.1784	
H		3.738	3.9234	3.903	3.9278	3.8839	
H		1.4413	1.3365	1.3308	1.3408	1.3211	
H		1.8648	2.1346	2.1779	2.1593	2.1623	
H		1.1767	1.2622	1.2737	1.2689	1.2439	
H		0.9348	0.8922	0.87	0.8844	0.8846	
H		1.3618	1.4222	1.4309	1.4444	1.4426	
H		1.4141	1.5796	1.587	1.6077	1.5966	
H		1.4107	1.3059	1.3181	1.3236	1.3117	
H		1.5706	1.4175	1.4164	1.4277	1.4098	

Functional	Solvent?		Basis Set		Type of Data	
mPW1PW91	PCM		6-31+G(d,p)		Unscaled Shifts	
	Isomer 1	Isomer 2	Isomer 3	Isomer 4	Isomer 5	Isomer 6
sDP4+ (H data)	4.04%	0.72%	0.78%	94.46%	-	-
sDP4+ (C data)	20.47%	17.55%	41.58%	20.41%	-	-
sDP4+ (all data)	4.02%	0.61%	1.58%	93.79%	-	-
uDP4+ (H data)	3.07%	3.70%	6.87%	86.37%	-	-
uDP4+ (C data)	8.88%	27.44%	19.63%	44.04%	-	-
uDP4+ (all data)	0.67%	2.49%	3.32%	93.52%	-	-
DP4+ (H data)	0.15%	0.03%	0.07%	99.75%	-	-
DP4+ (C data)	7.64%	20.24%	34.32%	37.79%	-	-
DP4+ (all data)	0.03%	0.02%	0.06%	99.89%	-	-
